# Human IRF1 governs macrophagic IFN-γ immunity to mycobacteria

**DOI:** 10.1016/j.cell.2022.12.038

**Published:** 2023-02-02

**Authors:** Jérémie Rosain, Anna-Lena Neehus, Jeremy Manry, Rui Yang, Jérémie Le Pen, Wassim Daher, Zhiyong Liu, Yi-Hao Chan, Natalia Tahuil, Özden Türel, Mathieu Bourgey, Masato Ogishi, Jean-Marc Doisne, Helena M. Izquierdo, Takayoshi Shirasaki, Tom Le Voyer, Antoine Guérin, Paul Bastard, Marcela Moncada-Velez, Ji Eun Han, Taushif Khan, Franck Rapaport, Seon-Hui Hong, Andrew Cheung, Kathrin Haake, Barbara C. Mindt, Laura Perez, Quentin Philippot, Danyel Lee, Peng Zhang, Darawan Rinchai, Fatima Al Ali, Manar Mahmoud Ahmad Ata, Mahbuba Rahman, Jessica N. Peel, Søren Heissel, Henrik Molina, Yasemin Kendir-Demirkol, Rasheed Bailey, Shuxiang Zhao, Jonathan Bohlen, Mathieu Mancini, Yoann Seeleuthner, Marie Roelens, Lazaro Lorenzo, Camille Soudée, María Elvira Josefina Paz, Maria Laura Gonzalez, Mohamed Jeljeli, Jean Soulier, Serge Romana, Anne-Sophie L’Honneur, Marie Materna, Rubén Martínez-Barricarte, Mathieu Pochon, Carmen Oleaga-Quintas, Alexandre Michev, Mélanie Migaud, Romain Lévy, Marie-Alexandra Alyanakian, Flore Rozenberg, Carys A. Croft, Guillaume Vogt, Jean-François Emile, Laurent Kremer, Cindy S. Ma, Jörg H. Fritz, Stanley M. Lemon, András N. Spaan, Nicolas Manel, Laurent Abel, Margaret R. MacDonald, Stéphanie Boisson-Dupuis, Nico Marr, Stuart G. Tangye, James P. Di Santo, Qian Zhang, Shen-Ying Zhang, Charles M. Rice, Vivien Béziat, Nico Lachmann, David Langlais, Jean-Laurent Casanova, Philippe Gros, Jacinta Bustamante

**Affiliations:** 1 Laboratory of Human Genetics of Infectious Diseases, Inserm U1163, 75015 Paris, France; 2 Paris Cité University, Imagine Institute, 75015 Paris, France; 3 Institute of Experimental Hematology, REBIRTH Center for Regenerative and Translational Medicine, Hannover Medical School, 30625 Hannover, Germany; 4 St. Giles Laboratory of Human Genetics of Infectious Diseases, The Rockefeller University, New York, NY 10065, USA; 5 Laboratory of Virology and Infectious Disease, The Rockefeller University, New York, NY 10065, USA; 6 Infectious Disease Research Institute of Montpellier (IRIM), Montpellier University, 34000 Montpellier, France; 7 Inserm, IRIM, 34293 Montpellier, France; 8 Department of Immunology, Del Niño Jesus Hospital, T4000 San Miguel de Tucuman, Tucuman, Argentina; 9 Department of Pediatric Infectious Disease, Bezmialem Vakif University Faculty of Medicine, 34093 İstanbul, Turkey; 10 Dahdaleh Institute of Genomic Medicine, McGill University, Montreal, QC H3A 0G1, Canada; 11 Canadian Centre for Computation Genomics, Montreal, QC H3A 0G1, Canada; 12 Innate Immunity Unit, Institut Pasteur, 75015 Paris, France; 13 Inserm U1223, 75015 Paris, France; 14 Institut Curie, PSL Research University, Inserm U932, 75005 Paris, France; 15 Department of Medicine, Lineberger Comprehensive Cancer Center, University of North Carolina at Chapel Hill, Chapel Hill, NC 27599-7292, USA; 16 Garvan Institute of Medical Research, Darlinghurst, NSW 2010, Australia; 17 St. Vincent’s Clinical School, Faculty of Medicine, University of NSW, Sydney, NSW 2052, Australia; 18 Pediatric Hematology-Immunology and Rheumatology Unit, Necker Hospital for Sick Children, AP-HP, 75015 Paris, France; 19 Department of Immunology, Sidra Medicine, Doha, Qatar; 20 Department of Microbiology and Immunology, McGill University, Montreal, QC H3A 0G1, Canada; 21 McGill University Research Centre on Complex Traits, McGill University, Montreal, QC H3A 0G1, Canada; 22 FOCiS Centre of Excellence in Translational Immunology, McGill University, Montreal, QC H3A 0G1, Canada; 23 Department of Immunology and Rheumatology, “J. P. Garrahan” National Hospital of Pediatrics, C1245 CABA, Buenos Aires, Argentina; 24 Proteomics Resource Center, The Rockefeller University, New York, NY 10065, USA; 25 Umraniye Education and Research Hospital, Department of Pediatric Genetics, 34764 İstanbul, Turkey; 26 Study Center for Primary Immunodeficiencies, Necker Hospital for Sick Children, AP-HP, 75015 Paris, France; 27 Paris Cité University, 75006 Paris, France; 28 Department of Pediatric Pathology, Del Niño Jesus Hospital, T4000 San Miguel de Tucuman, Tucuman, Argentina; 29 Central Laboratory, Del Niño Jesus Hospital, T4000 San Miguel de Tucuman, Tucuman, Argentina; 30 Cochin University Hospital, Biological Immunology Unit, AP-HP, 75014 Paris, France; 31 Inserm/CNRS U944/7212, Paris Cité University, 75006 Paris, France; 32 Hematology Laboratory, Saint-Louis Hospital, AP-HP, 75010 Paris, France,; 33 National Reference Center for Bone Marrow Failures, Saint-Louis and Robert Debré Hospitals, 75010 Paris, France; 34 Rare Disease Genomic Medicine Department, Paris Cité University, Necker Hospital for Sick Children, 75015 Paris, France; 35 Department of Virology, Paris Cité University, Cochin Hospital, 75014 Paris, France; 36 Division of Genetic Medicine, Department of Medicine, Vanderbilt Genetics Institute, Vanderbilt University Medical Center, Nashville, TN 37232, USA; 37 Department of Pathology, Microbiology, and Immunology, Vanderbilt Center for Immunobiology, Vanderbilt Institute for Infection, Immunology, and Inflammation, Vanderbilt University Medical Center, Nashville, TN 37232, USA; 38 Immunology Laboratory, Necker Hospital for Sick Children, AP-HP, 75015 Paris, France; 39 Inserm UMR1283, CNRS UMR8199, European Genomic Institute for Diabetes, Lille University, Lille Pasteur Institute, Lille University Hospital, 59000 Lille, France; 40 Neglected Human Genetics Laboratory, Paris Cité University, 75006 Paris, France; 41 Pathology Department, Ambroise-Paré Hospital, AP-HP, 92100 Boulogne-Billancourt, France; 42 Department of Physiology, McGill University, Montreal, QC H3A 0G1, Canada; 43 Department of Medical Microbiology, University Medical Center Utrecht, Utrecht University, 3584CX Utrecht, The Netherlands; 44 College of Health and Life Sciences, Hamad Bin Khalifa University, Doha, Qatar; 45 Department of Pediatric Pulmonology, Allergology and Neonatology and Biomedical Research in Endstage and Obstructive Lung Disease, German Center for Lung Research, Hannover Medical School, 30625 Hannover, Germany, EU; 46 Cluster of Excellence RESIST (EXC 2155), Hannover Medical School, 30625 Hannover, Germany; 47 Department of Human Genetics, McGill University, Montreal, QC H3A 0G1, Canada; 48 Department of Pediatrics, Necker Hospital for Sick Children, Assistance Publique Hôpitaux de Paris (AP-HP), 75015 Paris, France; 49 Howard Hughes Medical Institute, New York, NY 10065, USA; 50 Department of Biochemistry, McGill University, Montreal, QC H3A 0G1, Canada; 51 Lead contact

**Keywords:** Inborn errors of immunity, *Mycobacterium*, interferon-γ, interferon-stimulated gene, IRF1, viruses

## Abstract

Inborn errors of human IFN-γ-dependent macrophagic immunity underlie mycobacterial diseases, whereas inborn errors of IFN-α/β-dependent intrinsic immunity underlie viral diseases. Both types of IFNs induce the transcription factor IRF1. We describe unrelated children with inherited complete IRF1 deficiency and early-onset, multiple, life-threatening diseases caused by weakly virulent mycobacteria and related intramacrophagic pathogens. These children have no history of severe viral disease, despite exposure to many viruses, including SARS-CoV-2, which is life-threatening in individuals with impaired IFN-α/β immunity. In leukocytes or fibroblasts stimulated *in vitro*, IRF1-dependent responses to IFN-γ are, both quantitatively and qualitatively, much stronger than those to IFN-α/β. Moreover, IRF1-deficient mononuclear phagocytes do not control mycobacteria and related pathogens normally when stimulated with IFN-γ. By contrast, IFN-α/β-dependent intrinsic immunity to nine viruses, including SARS-CoV-2, is almost normal in IRF1-deficient fibroblasts. Human IRF1 is essential for IFN-γ-dependent macrophagic immunity to mycobacteria, but largely redundant for IFN-α/β-dependent antiviral immunity.

## In Brief

Studies in humans with interferon regulatory factor 1 (IRF1) deficiency reveal differences in how by type I and type II interferon immune responses protect humans against different types of pathogens.

## Graphical Abstract

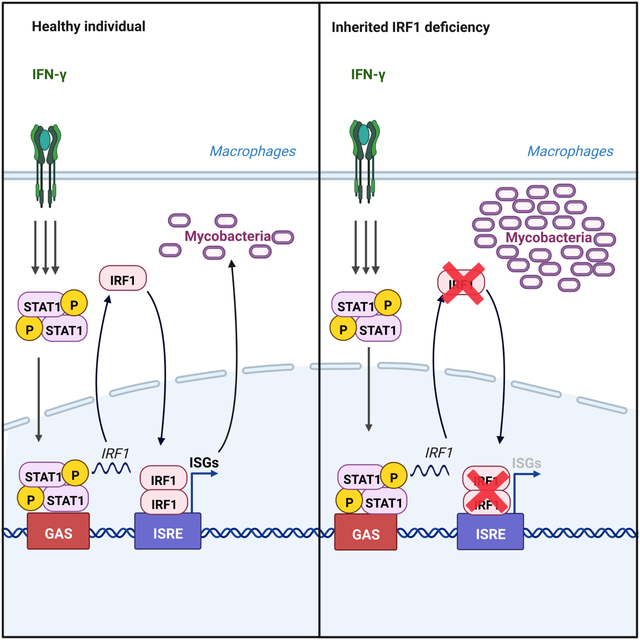

## Introduction

The discovery of inborn errors of immunity (IEI) underlying severe infectious diseases delineates the essential *versus* redundant functions of the corresponding human genes in host defense *in natura*, while clarifying the pathogenesis of these infections^1–3^. Mendelian susceptibility to mycobacterial disease (MSMD) is the most extensively studied monogenic susceptibility to a single type of infection in otherwise healthy individuals with apparently normal resistance to most other infections. Patients with MSMD are selectively vulnerable to weakly virulent mycobacteria — bacillus Calmette-Guérin (BCG) vaccines and environmental mycobacteria (EM) — and, in some cases, *Mycobacterium tuberculosis* and other intramacrophagic microorganisms^4–9^. MSMD is typically “isolated”, but can occasionally be “syndromic”, if associated with at least one other key infectious or non-infectious clinical phenotype^4^. Mutations of 19 different genes can account for MSMD: *CYBB*, *IFNG, IFNGR1, IFNGR2, IL12B*, *IL12RB1*, *IL12RB2*, *IL23R*, *NEMO, SPPL2A*, and *TBX21* for isolated MSMD; *ISG15*, *JAK1*, *RORC, TYK2, USP18* and *ZNFX1* for syndromic MSMD; and *IRF8*, *STAT1*, and *TYK2* for isolated or syndromic MSMD depending on the mutation^4,6–8,10^. Allelic forms at these 19 loci define 35 genetic etiologies of MSMD. Eighteen of the 19 known MSMD-causing genes encode products involved in the production of interferon-γ (IFN-γ) (*IFNG, IL12B*, *IL12RB1*, *IL12RB2*, *IL23R*, *ISG15*, *RORC, TBX21, TYK2*), cellular responses to IFN-γ (*CYBB*, *JAK1, IFNGR1*, *IFNGR2*, *STAT1, USP18*), or both (*IRF8, NEMO, SPPL2A*) (Figure S1A). *ZNFX1* is the only gene for which the mechanism of MSMD is not yet understood^6^. Thus, MSMD is typically caused by IEI of IFN-γ immunity. These conditions display a high level of genetic and allelic heterogeneity, but striking physiological homogeneity^11^.

However, only three etiologies of isolated MSMD are truly Mendelian, *i.e*. with complete clinical penetrance^1^: autosomal recessive (AR) complete IFN-γ, IFN-γR1, and IFN-γR2 deficiencies (Figure S1B)^4,5,12–15^. These three disorders abolish IFN-γ activity. The corresponding patients have early-onset, disseminated, recurrent, multiple, and life-threatening mycobacterial infections^4,5,12^. The other etiologies of isolated MSMD have both incomplete clinical penetrance and less severe clinical features, with a later onset, narrower range of pathogens, lower rate of recurrence, and better outcome. Overall, lower penetrance and lesser severity are associated with higher levels of residual IFN-γ activity (Figure S1B). For instance, AR partial IFN-γR1 or IFN-γR2 deficiencies, which impair but do not abolish cellular responses to IFN-γ, are less severe than the complete forms^12,16^. AR complete IL-12Rβ1 and IL-12p40 deficiencies are also milder conditions; they abolish cellular responses to IL-12 and IL-23, reducing IFN-γ production to 1–10% of normal levels^17–19^. AR complete IL-12Rβ2 and IL-23R deficiencies are even milder and less penetrant, as there is a selective defect of the response to IL-12 or IL-23^20,21^ (Philippot *et al*., under revision). Despite being recessively inherited complete deficiencies, these four conditions are less penetrant and severe than recessive defects of IFN-γ or its receptor^12,19,20,22^. Finally, homozygosity for *TYK2* p.P1104A, which impairs but does not abolish IFN-γ induction in response to IL-23 only, is associated with the lowest known penetrance of MSMD (below 0.5%)^23^. Thus, studies of MSMD have revealed that human antimycobacterial immunity is a genetically controlled quantitative trait: the lower the level of IFN-γ activity, the more severe the disease and the higher its penetrance^16^.

Less is known about the cellular basis of human immunity to mycobacteria^1^. Studies of peripheral leukocytes from MSMD patients have suggested that some IFN-γ-producing lymphocyte subsets, either alone or in combination, are essential for antimycobacterial immunity^8,24–26,20^. These essential subsets include the combination of natural killer (NK) cells, γδ T cells, and type 1 and type 2 innate lymphoid cells (ILC1 and ILC2) impaired in IL-12Rβ2 deficiency^20^; the invariant NK (iNKT) and mucosal-associated invariant T (MAIT) cells impaired in IL-23R^20,21^ (Philippot *et al*., under revision), RORγ/RORγT^24^, IL-12Rβ1, and TYK2 deficiencies^20,23^; the T_H_1* cells impaired in RORγ/RORγT, IRF8 and SPPL2a deficiencies^24,25^; the γδ T cells impaired in RORγ/RORγT^24^; the γδ2^+^ T cells impaired in IL-12Rβ1, and IL-23R deficiencies^20^ (Philippot *et al*., under revision); and the combination of NK, iNKT, MAIT and Vδ2^+^ γδ T cells impaired in T-bet deficiency^8^. These studies also suggested that IL-12- and IL-23-producing type 2 dendritic cells (DCs), which are impaired in both AR SPPL2a^25^ and autosomal dominant (AD) IRF8 deficiencies^25,27^, are essential for antimycobacterial immunity. Myeloid cells induce IFN-γ in lymphoid cells via at least ISG15^26^, IL-12^20,23^, and IL-23^20,23^, promoting the development of IFN-γ-producing cells, such as T_H_1* cells^25^. They are also activated by IFN-γ, which is commonly considered more of a macrophage-activating factor^28^ than an antiviral IFN. IFN-γ controls mycobacterial growth within macrophages via *JAK1*, STAT1 and the NAPDH oxidase complex, as revealed by studies of MSMD-causing *JAK1*, *STAT1* and *CYBB* mutations^9,29–34^. We investigated the cellular and molecular basis of human immunity to mycobacteria further, by studying two unrelated children with a severe and unexplained form of isolated MSMD, combining diseases due to both BCG and *Mycobacterium avium* in early childhood.

## Results

### Two unrelated children with severe mycobacterial diseases

**Figure 1 –:**
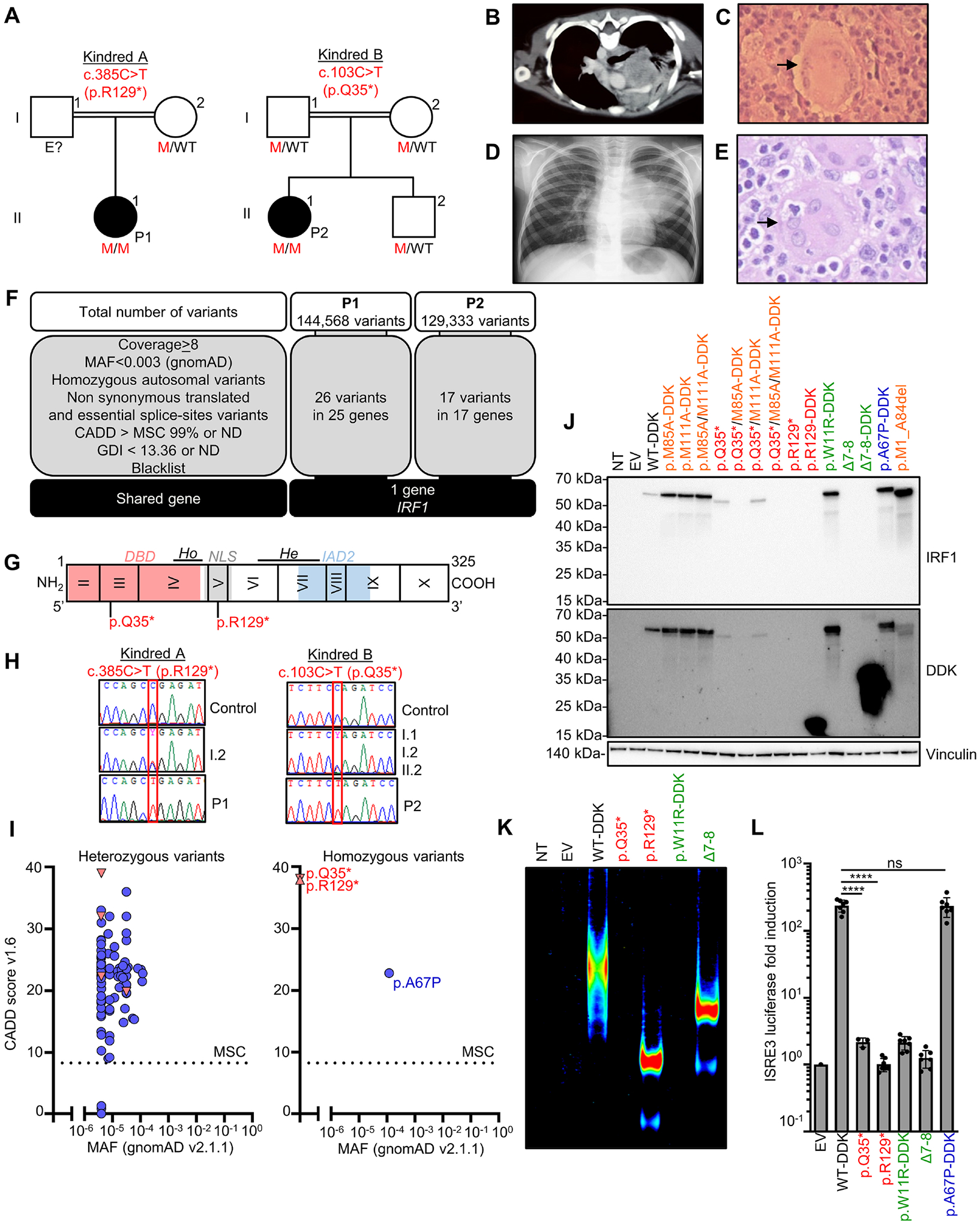
Homozygous complete loss-of-function *IRF1* variant in patients with severe MSMD. **(A)** Pedigrees of the two consanguineous kindreds. M=mutated; WT= wild-type; **(B)** Chest CT scan (P1) showing pulmonary infection. **(C)** Hematoxylin and eosin staining of a lymph node (P1) during *M. avium* and *H. capsulatum* infections, showing a multinucleated giant cell. **(D)** Chest X ray for P2 showing pulmonary *M. avium* infection. **(E)** Hematoxylin-eosin-saffron stain of a lung biopsy (P2) showing a giant cell engulfing another cell by phagocytosis (arrow). **(F)** WES analysis of P1 and P2. **(G)** IRF1 protein with the DNA-binding domain (DBD), nuclear localization sequence (NLS), and IRF-associated domain type 2 in blue (IAD2). Ho = homodimerization domain; He = heterodimerization domain. **(H)** Electropherograms of representative *IRF1* nucleotide sequences. **(I)** CADD score vs. minor allele frequency (MAF) for the heterozygous state in gnomAD for variants *IRF1* (left), and (right) for a homozygous variant from BRAVO/TOPmed (p.A67P), and the variants present in patients. Missense variants are indicated by blue circles and predicted loss-of-function variants are indicated by red triangles. **(J)** Western blotof total lysate from HEK293T cells with and without transfection with various C-DDK-tagged *IRF1* cDNAs or with empty vector (EV). NT = not transfected. Representative data from two independent experiments. **(K)** EMSA on nuclear extract of HEK293T cells transfected with EV, WT or mutant *IRF1* cDNAs, incubated with an ISRE probe. Representative data from two independent experiments. **(L)** Dual luciferase ISRE3 reporter activity of HEK293T cells transfected with EV or mutant *IRF1* cDNAs. Data from 3–7 independent experiments performed in triplicate. Bars represent the mean and standard deviation (SD). Statistical analysis by Student’s *t*-test. ns = not significant, *p* > 0.05; ****p < 0.0001.

We studied two unrelated children, P1 and P2. Both of them displayed severe forms of MSMD, with not only BCG disease, but also *M. avium* complex disease at a very young age, with four episodes of mycobacterial disease in P1, and two episodes in P2, before the age of six years in both cases (Figures 1A–E). Phenotypes of this severity are seen almost exclusively in patients with recessive complete defects of IFN-γ, either of the receptor chains, or STAT1. Both P1 and P2 had no history of unusually severe viral illness and the only other infection observed, histoplasmosis, is caused by an intramacrophagic fungus that has already been reported in other patients with MSMD^4–6,8^ (see Supplemental case report). We performed whole-exome sequencing (WES) on the two patients. The ethnicity of the two patients was confirmed by principal component analysis (PCA) on the WES data^35^ (Figure S1C). The homozygosity rates for P1 and P2 were 2.74% and 4%, respectively, suggesting that the parents were probably first- or second-degree cousins^36^. The parents being healthy, we tested the hypothesis of an AR disorder and considered homozygous variants on autosomes. The prevalence of MSMD is about 10^−5^. We therefore considered variants with a minor allele frequency (MAF) below 0.003 in gnomAD v2.1.1. We then selected non-synonymous and essential splice-sites variants predicted to have a combined annotation depletion-dependent (CADD) score above the 99% mutation significance cut-off (MSC)^37^, for genes with a gene damage index (GDI) below the cutoff of 13.36 for inborn errors with AR inheritance^38^ (Figure 1F). We also filtered out false-positive rare variants, which were absent or rare in public databases but had a frequency above 1% in our in-house cohort (the ‘blacklist’)^39^. We found no homozygous candidate copy number variants (CNVs) in known MSMD genes^40^. We identified 26 rare homozygous single-nucleotide variants (SNVs) in 25 genes in P1, and 17 homozygous SNVs in 17 genes in P2 (Figure 1F and Table S1). In tests of the hypothesis of genetic homogeneity, we found only one common gene: *IRF1*. Both patients displayed homozygous transitions predicted to be nonsense in the *IRF1* canonical transcript (NM_002198.2^41^, GRCh37): c.385C>T (p.R129*) in P1, and c.103C>T (p.Q35*) in P2 (Figure 1G). Sanger sequencing confirmed that P1 was homozygous for p.R129* and P2 was homozygous for p.Q35*, whereas asymptomatic relatives were heterozygous (Figures 1A and 1H). These findings suggest that MSMD in P1 and P2 resulted from homozygosity for these rare nonsense *IRF1* variants.

### The overexpressed IRF1 p.Q35* and p.R129* mutants are loss-of-function

*IRF1* encodes interferon regulatory factor 1 protein (IRF1), a transcription factor induced by IFN-γ, the deficiency of which in mice underlies susceptibility to various pathogens, including mycobacteria^43,49,51,53,55,57,62,83,84,96–98,109,110,147^ (Table S2). IRF1 is a 325-amino acid transcription factor composed of an amino-terminal DNA-binding domain (DBD) that can bind to DNA interferon-stimulated response elements (ISRE) or positive regulatory domain I (PRDI) motifs^64^, an intermediate segment containing a putative nuclear localization sequence (NLS), and a carboxyterminal IRF association domain 2 (IAD2), which is crucial for transcriptional activity^65,66,68,70^ (Figure 1G). Both the c.385C>T and c.103C>T variants are predicted to be loss-of-function (pLOF) and are rare or private in public databases (Figure 1E–I and Supplemental information). We studied the impact of the *IRF1* variants by transiently transfecting human embryonic kidney (HEK)293T cells with plasmids encoding WT *IRF1* (NM_002198.2), mutants p.R129* and p.Q35*, or p.A67P cDNAs. We used two previously described LOF mutants isolated from human tumors as negative controls: (i) the missense mutant p.W11R^42^, and (ii) a frameshift mutant resulting in the deletion of exons 7 and 8^44^ (hereafter referred to as Δ7–8). The proteins encoded by the constructs were left untagged or were tagged with DDK at the carboxy-terminus (Figure S1E). Immunoblotting of cell extracts showed that the WT-DDK, p.W11R-DDK, and p.A67P-DDK proteins were produced at a molecular weight (MW) slightly above 50 kDa, as expected, whereas the p.R129-DDK and the Δ7–8-DDK proteins had a lower MW (Figure 1J). These results indicate that the p.R129-DDK cDNA encodes a truncated protein. Both p.R129-DDK and Δ7–8-DDK gave bands of higher intensity than the WT protein, consistent with an absence of the carboxyterminal degradation domain^45^. Immunoblotting with a monoclonal antibody (mAb) directed against the C-terminus of IRF1 showed no re-initiation of translation with the p.R129* cDNA (Figure 1J). However, immunoblotting of the p.Q35* protein showed this protein to have a slightly lower MW than the WT protein when probed with either the anti-IRF1 or anti-DDK mAb, suggesting that a re-initiation of translation had occurred. Two ATG codons downstream from p.Q35 and upstream from p.R129 (p.M85 and p.M111) were predicted to be potential translation re-initiation sites in analyses *in silico*. We mutated the corresponding methionine (ATG) codon to an alanine codon (GCG). With the WT-DDK cDNA template, the mutation of p.M85 (WT/M85A-DDK), p.M111 (WT/M111A-DDK), or both (WT/M85A/M111A-DDK) had no effect on the MW of the protein produced (Figure 1J). Conversely, with the p.Q35* cDNA template, the mutation of p.M85 (p.Q35*/M85A-DDK) abolished protein detection, whereas the mutation of p.M111 (p.Q35*/M111A-DDK) did not. The mutation of both methionine (p.Q35*/M85A/M111A-DDK) residues abolished protein production (Figure 1J). Consistently, the deletion of all amino acids upstream from M85A (p.M1_A84del) resulted in the production of a protein of the same MW as p.Q35* (Figure 1J). Overall, these results suggest that translation is re-initiated for the p.Q35* variant, resulting in the production of a protein lacking the first 84 amino-terminal amino acids of the DBD. Both the mutant proteins from the patients localized to the nucleus (Figure S1G). An EMSA with an ISRE probe found that the p.Q35* protein, like the previously described p.W11R mutant protein^42^, did not bind DNA, whereas the p.R129-DDK mutant, which retained the DBD, was able to bind DNA, resulting in a band of higher mobility than was observed for the WT, consistent with the lower MW of the mutant protein (Figure 1K and S1H). We then assessed the transcriptional activity of IRF1 in a dual luciferase assay with two different plasmids containing two different ISRE repeats. The p.A67P-DDK cDNA induced luciferase to WT levels, whereas the p.R129* and p.Q35* mutants and two negative controls (p.W11R-DDK^42^ and Δ7–8^44^) did not (Figures 1L and S1I). Overall, these results suggest that the p.R129* (P1) and p.Q35* (P2) variants impair IRF1 production and abolish its transcriptional activity.

### IRF1 protein expression is impaired in cells of the patients

**Figure 2 –:**
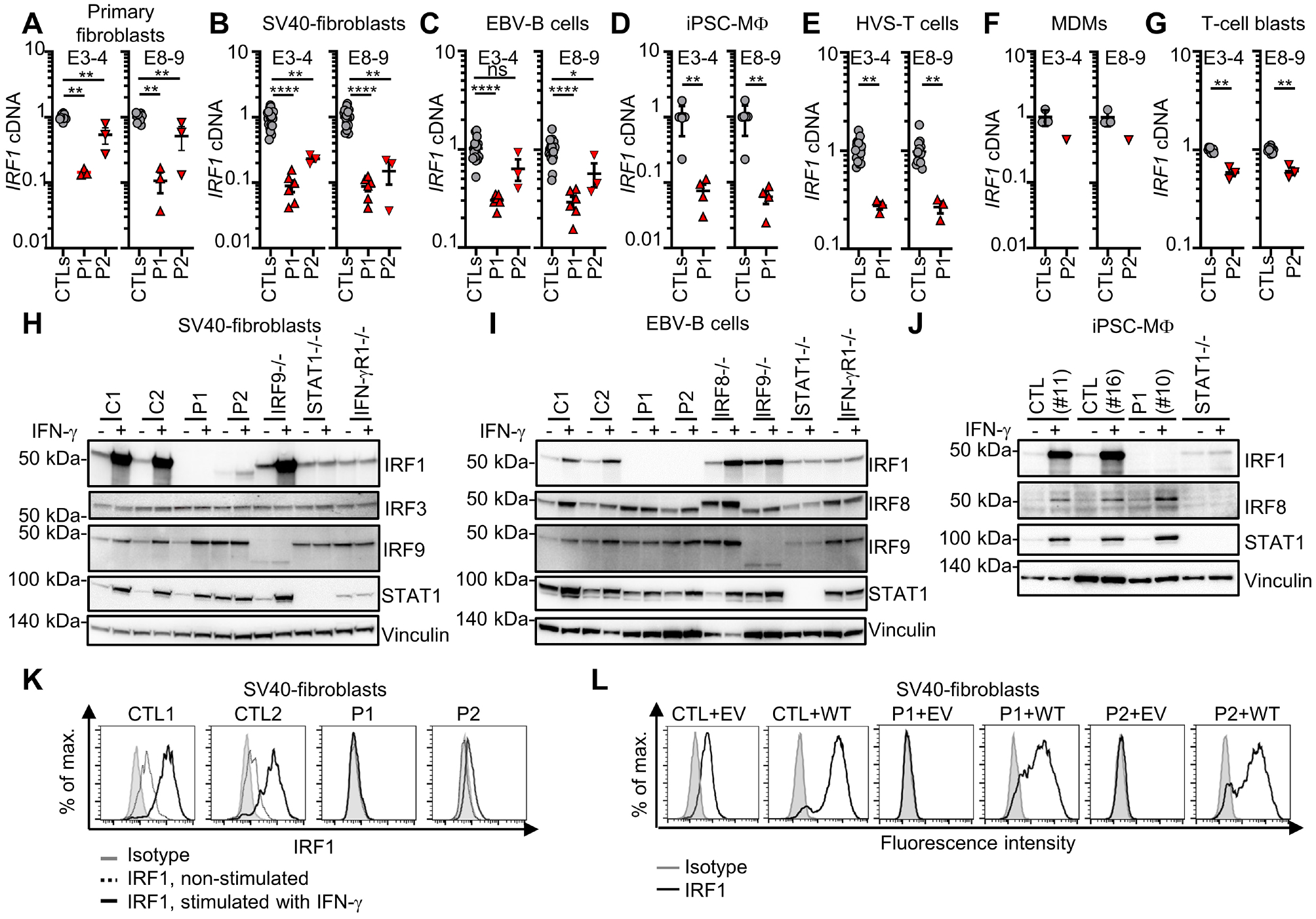
IRF1 mRNA and protein levels in cells from the two patients. Quantitative PCR for *IRF1* normalized against *GUSB* and the mean value for controls (CTLs) for cDNA from **(A)** primary fibroblasts, **(B)** SV40-fibroblasts, **(C)** EBV-B cells, **(D)** iPSC-derived macrophages (iPSC-MΦ cells;), **(E)** HSV-T cells, **(F)** Monocytes-derived macrophages (MDMs), and **(G)** T-cell blasts. Bars represent the mean and SD. Western blot for indicated protein in total lysate from **(H)** SV40-fibroblasts, **(I)** EBV-B cells, or **(J)** iPSC-derived MΦ, with and without IFN-γ stimulation. Data from 2–3 independent experiments are shown. **(K)** IRF1 staining and intracellular flow cytometry on SV40-fibroblasts with and without IFN-γ stimulation. **(L)** Flow cytometry with intracellular IRF1 staining on SV40-fibroblasts retrotransduced with an empty vector (EV) or WT) -*IRF1* cDNA. The data shown are representative of 2–3 independent experiments. Statistical analysis by Mann-Whitney tests. ns = not significant, *p* > 0.05; *p < 0.05; **p < 0.01; ****p < 0.0001.

IRF1 is produced ubiquitously in humans^46^, but its levels are highest in hematopoietic cells^46,47^. We used non-hematopoietic (primary fibroblasts, and simian virus 40 (SV40)-immortalized fibroblasts) and hematopoietic (Epstein-Barr virus-immortalized B lymphocytes (EBV-B cells), T-cell blasts, *Herpesvirus saimiri*-transformed T (HVS-T) cells, monocyte-derived macrophages (MDMs), and tissue macrophages derived from an induced pluripotent stem cell (iPSC) line^48,50^ (iPSC-MΦ)) to assess the impact of the p.R129* and p.Q35* *IRF1* variants on endogenous IRF1 mRNA and protein levels. We performed quantitative RT-PCR (RT-qPCR) with two sets of probes and found much lower levels of *IRF1* mRNA in the cells of P1 than in the corresponding cells from healthy controls, primary fibroblasts (6-fold lower; Figure 2A), SV40-fibroblasts (10-fold lower; Figure 2B), EBV-B cells (3-fold lower; Figure 2C), iPSC-MΦ (>10-fold lower; Figure 2D), and HVS-T cells (4-fold lower; Figure 2E), suggesting that the *IRF1* transcript underwent nonsense-mediated mRNA decay in the cells of P1. Conversely, *IRF1* mRNA levels in the cells of P2 (primary fibroblasts, SV40-fibroblasts, EBV-B cells, MDMs, and T-cell blasts) were only slightly lower than those in control cells (Figures 2A–C, and 2F–G). We then assessed IRF1 protein levels by immunoblotting and flow cytometry, with a mAb specific for the carboxy-terminus of IRF1. Primary and SV40-fibroblasts, EBV-B cells, HVS-T cells, and iPSC-MΦ from P1 contained no detectable endogenous IRF1 protein (Figures 2H–L and S2A–F). In addition, no truncated IRF1 protein was detected in SV40-fibroblasts from P1 after pretreatment with IFN-γ, even with a polyclonal antibody and prolonged exposure of the immunoblot (Figure S2F). The protein was barely detectable in primary and SV40-fibroblasts from P2 following pretreatment with IFN-γ (Figures 2H, 2K, and S2B), either on flow cytometry or after prolonged exposure of the immunoblot. The protein detected in the cells of P2 had a MW corresponding to that expected following a re-initiation of translation. Other transcription factors, such as IRF3, IRF8, IRF9, and STAT1, were produced by the cells of P1 and P2 (Figures 2H–J and S2A–F). The impaired basal levels of IRF1 protein production in the SV40-fibroblasts from both patients were corrected by stable transduction with the WT *IRF1* cDNA (Figure 2L). Together, these findings suggest that homozygosity for the *IRF1* p.R129* variant results in nonsense-mediated decay of the *IRF1* transcript and a lack of detectable protein, whereas homozygosity for the *IRF1* p.Q35* variant leads to a partial escape of mRNA nonsense-mediated decay, due to a re-initiation of translation, with low levels of production of an N-terminally truncated protein. P1 and P2 thus display an absence of IRF1 protein (P1) or the production of an abnormal IRF protein (P2) in hematopoietic and non-hematopoietic cells. These results further suggest that both patients have AR complete IRF1 deficiency.

### IRF1 deficiency alters the development of some innate or innate-like leukocyte subsets

**Figure 3 –:**
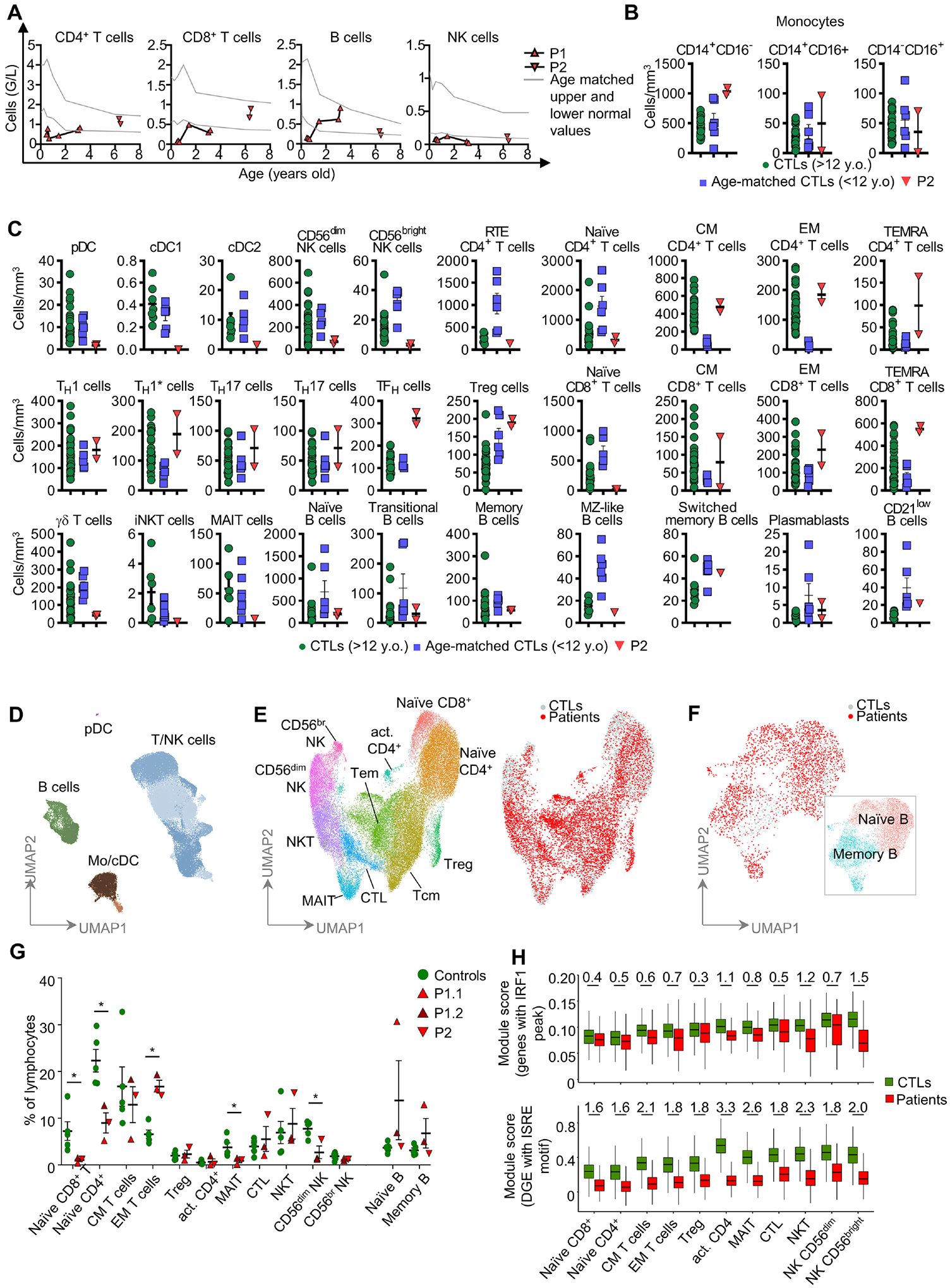
Phenotyping of peripheral blood leukocytes and single-cell PBMCs from IRF1-deficient patients. **(A)** Monitoring of lymphoid cell numbers in the whole blood or PBMCs of patients. **(B)** Counts for monocyte subsets by mass cytometry on fresh whole-blood cells. **(C)** Counts of myeloid and lymphoid subsets in fresh whole blood. **(D)** UMAP clustering for CTLs and samples from the IRF1-deficient patients (2x P1 and 1x P2) profiled by scRNA-seq or CITE-seq. General lineage populations are annotated. **(E)** Subclustering of T and NK cells to define cell subtypes. Overlay of 10,000 PBMCs from CTLs (gray) and 10,000 PBMCs from an IRF1-deficient patient (red) **(F)** As in (**E**), B-cell subclustering and cell-type annotation, together with an overlay of the patients’ and CTLs cells. **(G)** Proportions of each cell type expressed as a percentage of total lymphocytes. P1.1 corresponds to the first scRNA-seq analysis for P1 and P1.2 corresponds to the CITE-seq performed later on. **(H)** Module score analysis comparing the expression of genes with IRF1-binding sites within 10 kb of their TSS (top panel), and genes differentially expressed between patients and controls and predicted to have ISRE motifs in their promoters (bottom panel). Cohen’s *d* effect size estimates are shown for every significant variation of module expression. They were obtained by comparing the module score distribution in every cell subset in Wilcoxon signed-rank tests (*q*-value <= 0.05).

We investigated the role of human IRF1 in leukocyte development, by analyzing blood cells from P1 and P2. A complete blood count (CBC) showed that both P1 and P2 had counts of polymorphonuclear neutrophils (PMN), basophils (PMB), eosinophils (PME) and peripheral blood mononuclear cells (PBMCs) that were normal for age (Figures 3A and S3A). Conventional flow cytometry also showed normal counts of monocyte subsets in P2 (Figure 3B). Both patients had low percentages and counts of circulating conventional type 1 dendritic cells (cDC1) (>10-fold decrease) and cDC2 (~5-fold decrease), whereas the counts and percentages of plasmacytoid DCs (pDCs) were at the lower end of the normal range for both patients (Figures 3C and S3B–C). NK cell counts in both patients were 80% lower than those in age-matched controls. The NK cytopenia was explained by a depletion of both CD56^dim^ and NK CD56^bright^ NK cells (Figures 3A, 3C and S3B). The innate lymphoid cell compartment was also affected, with a decrease in the percentages of innate lymphoid cell precursors (ILCP) (~10-fold) and type 2 innate lymphoid cells (ILC2, ~5-fold) in both patients (Figures 3C and S3D). The frequencies and counts of iNKT cells were normal in both patients, whereas the percentage of MAIT cells was normal in P1 and slightly low in P2 (Figures 3C and S3B). Counts of γδ T cells were slightly lower in P2 than in age-matched controls (Figure 3C). The frequencies of Vδ1^+^ and Vδ2^+^ T cells as a proportion of total T cells were normal and low, respectively, in both patients (Figure S3B).

### IRF1 deficiency alters the development of certain adaptive leukocyte subsets

Both patients also had ~95% fewer naïve CD8^+^ T cells than controls, and an excess of memory and T EMRA cells (Figure 3C and Table S3), consistent with the homeostatic expansion of CD8^+^ T cells following exposure to infectious agents. CD4^+^ T-cell counts were normal in both patients, but the numbers of recent thymic emigrant CD4^+^ T cells were only a third those in controls (Figures 3A, 3C and Table S3). Counts and percentages of T_H_1, T_H_2, T_H_17, T_H_1*, and Treg cells were also normal in both patients, whereas TF_H_ cell counts were high in P2 (Figures 3C and S3B). B-cell counts were in the normal range in both patients, with normal frequencies of transitional B cells, memory B cells, and plasmablast cells (Figures 3A, 3C and S3B, and Table S3). The percentage of IgA^+^ memory B cells, was, however, higher than that in healthy controls, consistent with the abnormally high levels of plasma IgA detected in P1 (Figure S3B). However, both plasma IgA levels and IgA^+^ cell proportion were normal in P2. Both P1 and P2 had detectable antibodies against protein antigens and selected pneumococcal polysaccharide antigens in the serum, and normal serum levels of total IgG, and IgM (Table S4). Both patients displayed impaired development of myeloid dendritic cells (cDC1 and, to a much lesser extent, cDC2), innate lymphoid cells (ILC2 and ILCP), and IFN-γ-producing lymphoid cells (NK cells, naïve CD8^+^ T cells and, to a much lesser extent, naïve CD4^+^ T cells). Impaired development of cDC1, ILC2, NK, and CD8^+^ T cells were confirmed in IRF1-knockout mice (see Supplemental informations, Table S5, and Figures S3E–F).

### Impairment of the lymphoid cell transcriptomes of IRF1-deficient patients

We analyzed the leukocyte development by performing single-cell RNA-seq and/or cellular indexing of transcriptomes and epitopes by sequencing (CITE-seq) on cryopreserved PBMCs. Integrated sample clustering of the various immune subsets identified 13 different major lymphoid subsets: mature (CD56^dim^) and immature (CD56^bright^) NK cells, naïve CD4^+^ and CD8^+^ T cells, activated CD4^+^ T cells, central memory T cells, effector memory T cells, cytotoxic T cells, MAIT and NKT cells, Treg, and naïve and memory B cells (Figures 3D–F). Consistent with the results of mass and flow cytometry, quantification of these subsets revealed low percentages of CD56^dim^ NK cells, naïve CD4^+^ and CD8^+^ T cells, and an expansion of the population of effector memory T cells (Figures 3E–G). P1 also had an abnormally large fraction of IgA^+^ B cells, this feature being most pronounced at the age of three years (Figure 3G, Table S6). We performed differential gene expression analysis for each lymphoid subset, to detect changes in the transcriptional profiles of the cells of both patients. Interestingly, some genes were found to be differentially expressed in specific cells, but a sizable proportion of genes were differentially expressed in multiple subsets (Table S6). Gene ontology and pathway enrichment analysis indicated lower levels of expression for genes associated with terms such as leukocyte activation, defense response, cytokine signaling, cytotoxicity, and response to IFN in cells from the patients (Table S6). Moreover, an enrichment in genes with ISRE motifs in their promoters was observed for the genes underexpressed in patients, but not for those overexpressed in patients. We investigated the possible dysregulation of ISRE-containing gene expression, by performing a module score analysis in which we compared the expression of sets of genes between control and patient cells, and calculated effect size. We began the analysis by performing ChIP-seq on IFN-γ-stimulated mouse bone marrow-derived macrophages for the list of human orthologous genes binding IRF1. We found that IRF1-binding genes were significantly underexpressed in all subsets, this underexpression being strongest in memory and cytotoxic T cells and NK cells (Figure 3H). Underexpression was also more pronounced for differentially expressed genes with ISRE motifs in their promoters. However, *IFNG* expression was normal across lymphoid subsets (Table S6). Overall, these results suggest that IRF1 deficiency impairs the development of T and NK cells and the expression of target genes involved in immune activation.

### Mild impairment of IFN-γ production in IRF1-deficient lymphoid cells

**Figure 4 –:**
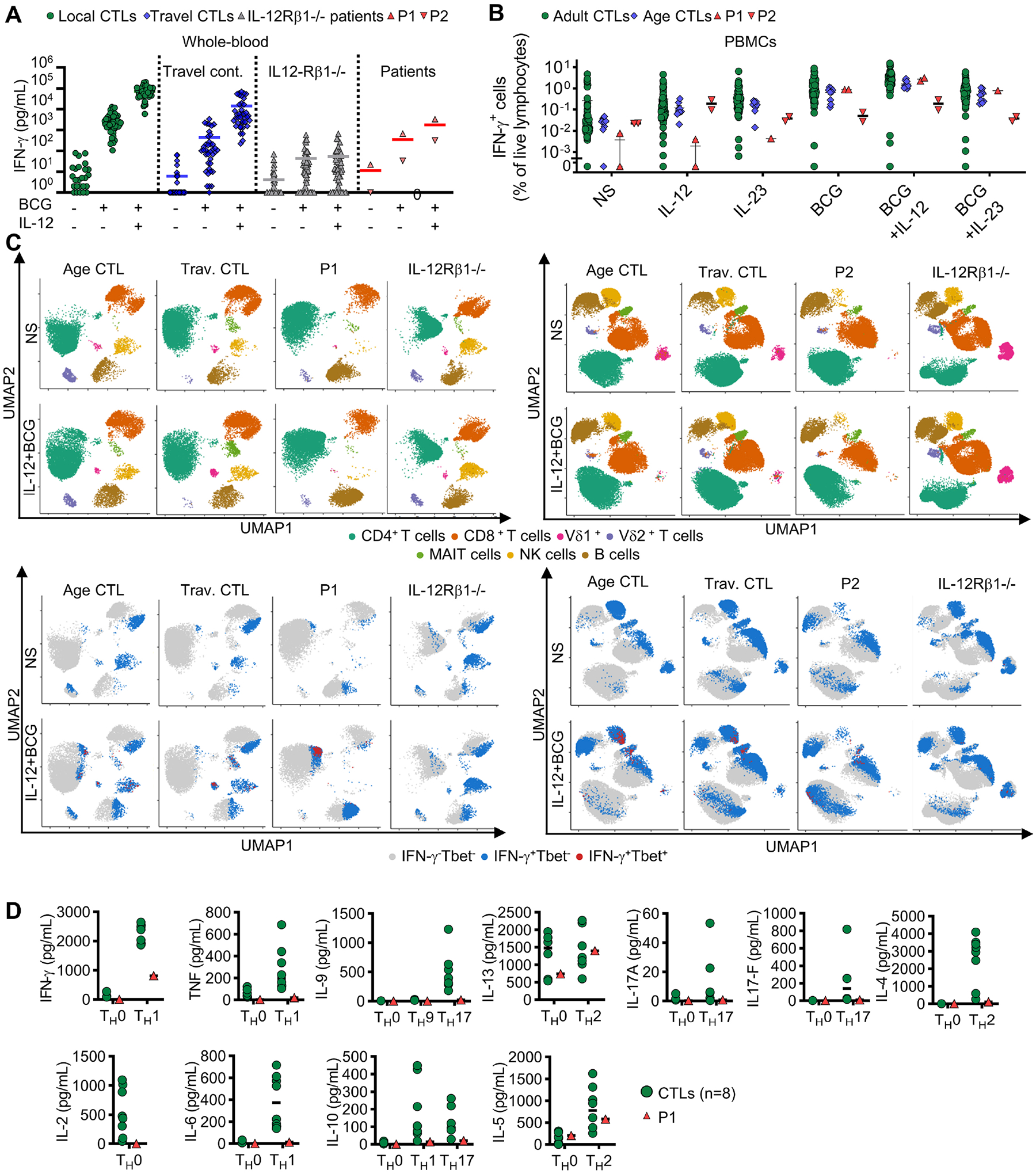
Production of IFN-γ by the lymphoid cells of IRF1-deficient patients. **(A)** Induction of IFN-γ secretion in a whole-blood assay, for controls (CTLs), IL-12Rβ1-deficient patients and patients. Bars represent the mean. **(B)** Intracellular flow cytometry on IFN-γ^+^ PBMCs after stimulation with IL-12, IL-23, or BCG. Bars represent the mean. Technical duplicates of the same experiment are shown for P1 and P2. **(C)** UMAP analysis of intracellular T-bet and IFN-γ expression by intracellular spectral flow cytometry across various PBMC subsets. Lymphoid subsets based on surface marker expression (upper panel), and their levels of IFN-γ and T-bet expression (lower panel). **(D)** Cytokine levels in the supernatant of naïve CD4^+^ T cells in polarizing conditions.

Given the impaired development of IFN-γ-producing lymphocytes and antigen-presenting DCs, we hypothesized that impaired IFN-γ production underlies mycobacterial disease in both these patients. We first investigated the IFN-γ secretion pathway in cell lines derived from patient cells. ISG15, the secretion of which is essential for IFN-γ production by NK cells^26^, was induced to a similar extent in the SV40-fibroblasts of both patients and those of healthy donors (Figure S4A). The early response to IL-12 — an inducer of IFN-γ production — which can be assessed by evaluating STAT4 phosphorylation, was also similar in the HVS-T cells of P1 and controls (Figure S4B). Moreover, IFN-γ production by HVS-T cells following stimulation with CD3/CD2/CD28 beads or PMA-ionomycin was similar for cells from P1 and controls (Figure S4C). We then studied the response to BCG of whole-blood samples from patients. IFN-γ was secreted by the peripheral leukocytes of both patients in response to BCG alone or BCG plus IL-12, and its levels in whole blood were similar to those of healthy local and travel controls (Figure 4A). Consistent with the results for whole blood, the stimulation of PBMCs from both patients with BCG in the presence or absence of IL-12 or IL-23 resulted in normal total intracellular IFN-γ production (Figure 4B). The clustering of IFN-γ-producing cells indicated that most of these cells were CD4^+^ and CD8^+^ T cells. However, the Vδ2^+^ cell and NK cell subsets of innate or innate-like adaptive lymphoid cells also displayed low levels of IFN-γ production in both patients (Figures 4C and S4D) consistent with their reduced circulating number. We then analyzed the *in vitro* differentiation of naïve CD4^+^ T cells from P1 after initial expansion with anti-CD2/CD3/CD28 mAb-coated beads and IL-2, followed by culture under T_H_0, T_H_1, T_H_2, T_H_9, or T_H_17 polarizing conditions. The production of IL-2 under T_H_0-polarizing conditions and the production of IFN-γ and TNF under T_H_1-polarizing conditions were strongly impaired in the IRF1-deficient naïve CD4^+^ T cells of P1 (Figures 4D and S4E). As a control, the production of IL-5 and IL-13 by IRF1-deficient naïve CD4^+^ T cells under T_H_2-polarizing conditions was normal (Figures 4D and S4E). Overall, IFN-γ production was impaired in NK and Vδ2^+^ cells, mostly due to their quantitative defects, and in T_H_1 cells upon non-mycobacterial stimulation, but the levels of production for this cytokine remained normal in CD4^+^ T cells, CD8^+^ T cells and whole blood cells stimulated with BCG.

### Impaired response to IFN-γ of IRF1-deficient fibroblasts

**Figure 5 –:**
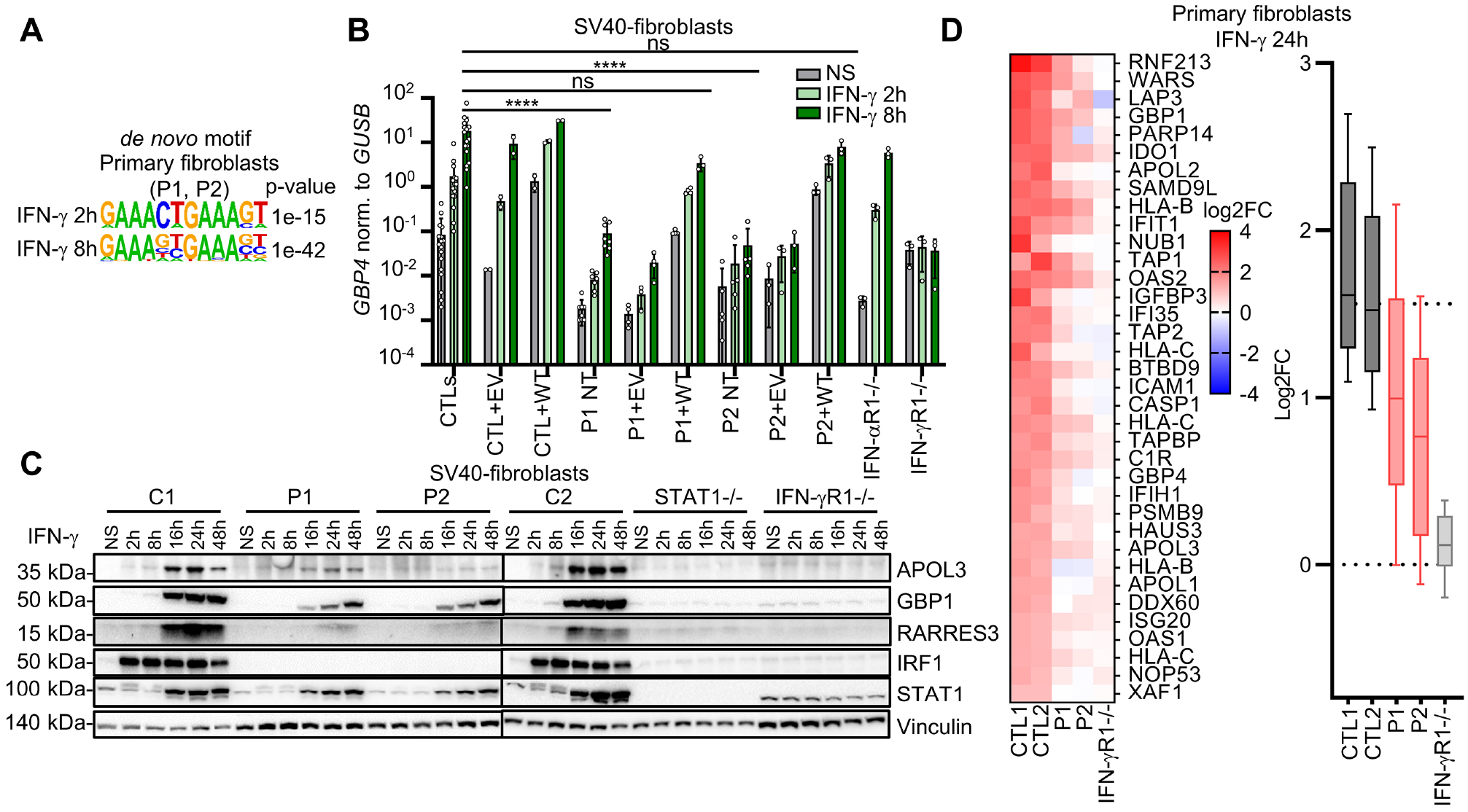
Response to IFN-γ of IRF1-deficient fibroblasts. **(A)** HOMER *de novo* motif analysis of the genes differentially expressed in the primary fibroblasts after IFN-γ stimulation. **(B)** RT-qPCR for *GBP4* (normalized against *GUSB*) in SV40-fibroblasts with or without retrotransduction with EV or WT *IRF1* cDNA and with or without stimulation IFN-γ. Data from 2–6 independent experiments are shown. Bars represent the mean and SD. **(C)** Immunoblots in SV40-fibroblasts with and without stimulation with IFN-γ. **(D)** Mass spectrometry on lysates of primary fibroblasts with and without IFN-γ stimulation. On the right, heatmaps for proteins (i) positively induced after stimulation with a log_2_FC>1 over the mean in the non-stimulated state for controls (ii) and a log_2_FC<0.5 over the mean in the non-stimulated state for patients. On the left, 10^th^-90^th^ percentiles for all proteins positively induced after stimulation with a log_2_FC>1 over the mean value in the non-stimulated state for controls.

IRF1 is a protein found predominantly in the nucleus in the resting state^52,54^. The levels of production of this protein regulate its activity^56,58–60^. IRF1 can bind as a monomer, homodimer, or heterodimer to ISRE motifs in target genes, inducing their expression, as shown in human^30,56,61,63^ and mouse cells^58^. IFN-γ is the strongest known stimulus increasing IRF1 protein levels, through the direct induction of *IRF1* mRNA synthesis^30,65^ and translation^56,58–60^, in a STAT1-dependent manner, within two to three hours^61,67^. We confirmed these findings for mRNA induction, translation and increases in gene expression following IFN-γ stimulation in SV40-fibroblasts (Figures S5A–C). We then investigated whether IRF1 deficiency impaired cellular responses to IFN-γ. As expected, the phosphorylation of STAT1 after 20 minutes of stimulation with IFN-γ, as determined by flow cytometry, was normal in EBV-B cells and SV40-fibroblasts from both patients (Figures S5D–E). We then comprehensively profiled the transcriptional response of the primary fibroblasts of patients after 30 minutes, 2 hours, and 8 hours of stimulation with IFN-γ, by RNA sequencing (RNA-seq). Primary fibroblasts from healthy donors displayed differential expression relative to non-stimulated conditions for 7 genes at 30 minutes, 343 genes at 2 hours, and 1,484 genes at 8 hours of stimulation, with *IRF1* among the differentially regulated genes at all these timepoints (Figures S5F–G, and Table S7). We found that 20% of these genes were expressed differently between patients and controls after 8 hours. However, this percentage was significantly lower at 2 hours (13%, *p*-value 0.0012), and after 30 minutes of stimulation (0%). We also found that, relative to controls, the cells of both patients produced 30% and 40% less RNA for IFN-γ inducible genes at 2 hours and 8 hours, respectively (Figure S5H). Among genes differentially expressed in cells of both patients, an enrichment in the expression of genes with ISRE motifs in their proximal promoters was observed at both 2 and 8 hours (Figure 5A), but it was stronger at the 8-hour timepoint (*p*-value 1e-42) than at 2 hours (*p*-value 1e-15) (Figure 5A). The impaired induction of *GBP4* transcription in response to IFN-γ was confirmed in SV40-fibroblasts from both patients (Figure 5B) and was rescued by stable transduction with a retroviral vector overexpressing WT *IRF1* (Figure 5B). The amounts of protein generated for four IRF1 target genes — as demonstrated by RNA-seq (APOL3, GBP1, RARRES3, and CD274), immunoblotting or flow cytometry of SV40-fibroblasts or primary fibroblasts from patients — were also lower than those for control cells (Figures 5C and S5I–K). The impaired induction of CD274, the ligand of PD1^69^, was corrected in patients’ SV40-fibroblasts by transduction of WT *IRF1* cDNA (Figures S5J–K). We also confirmed the impaired induction of several proteins, including GBP1, GBP4, and APOL3, in the primary fibroblasts of both patients, after 24 hours of stimulation with IFN-γ, as shown by mass spectrometry (Figure 5D). The mass of inducible protein was found to be 30% smaller in the patients’ cells (Figure 5D). These results suggest that IRF1 controls the enhancement of the second wave of response to IFN-γ, downstream from STAT1.

### Impaired response to IFN-γ in IRF1-deficient myeloid cells

**Figure 6 –:**
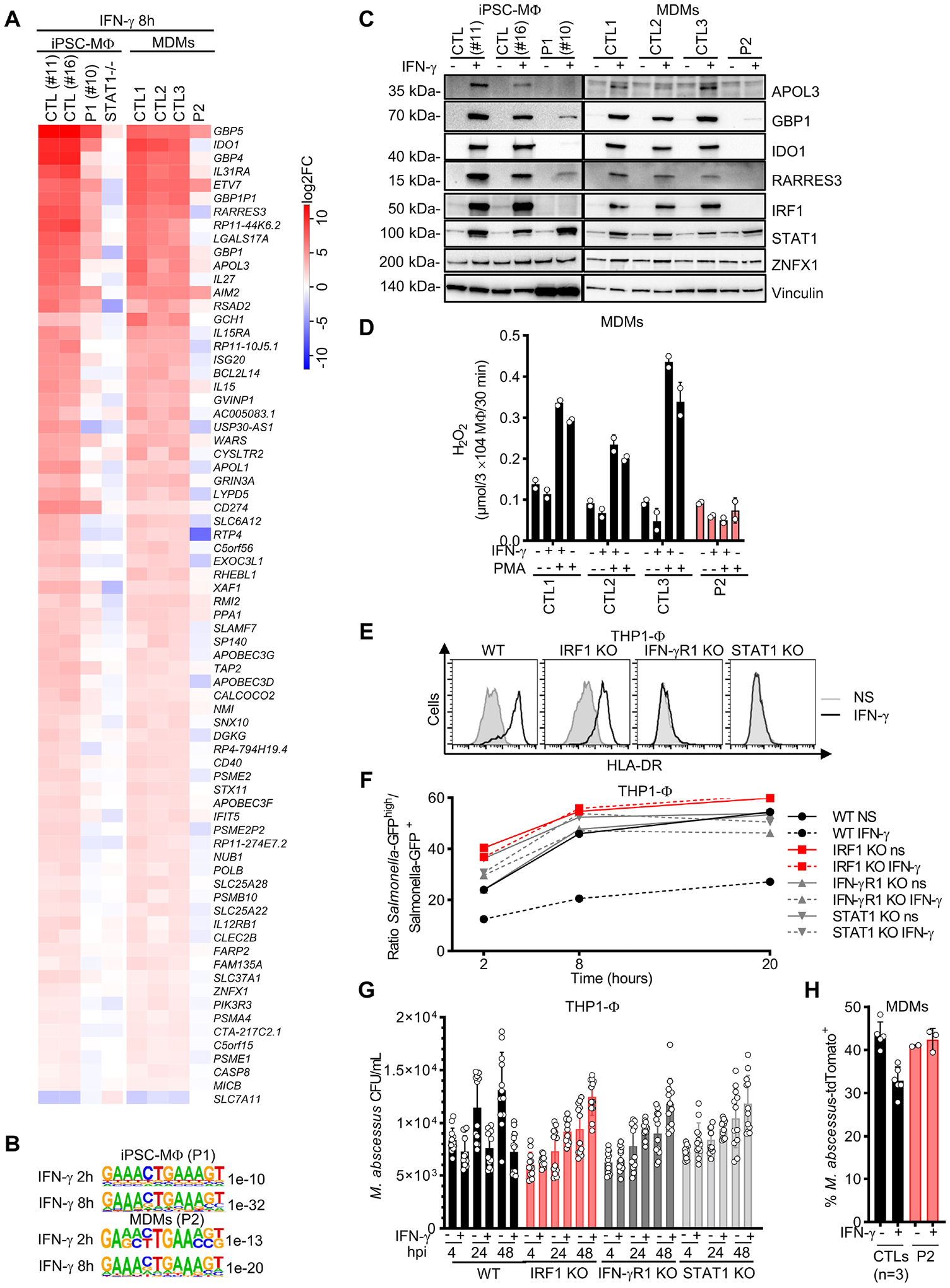
IFN-γ immunity in IRF1-deficient macrophages. **(A)** Heatmaps showing genes differentially expressed (|log_2_(FC)| > 1 and adj. *p*-value < 0.05) in CTL cells after 8 hours of stimulation with 10^3^ IU/mL IFN-γ, and differentially expressed in iPSC-MΦ (P1) and MDMs (P2) (|log_2_(FC)| > 1 and adj. *p*-value < 0.05). **(B)** HOMER *de novo* motif analysis of the genes differentially expressed in the iPSC-MΦ (P1), and in MDMs (P2); after IFN-γ stimulation. **(C)** Western blot of protein extracts in iPSC-MΦ, and MDMs, with and without stimulation with IFN-γ. **(D)** Extracellular H_2_O_2_ release for MDMs from CTLs and P2 (technical duplicates ± SD). **(E)** HLA-DR expression by flow cytometry, using THP1-Ф with and without IFN-γ stimulation. **(F)** Gentamicin protection assay performed on PMA-differentiated THP1-Ф with and without IFN-γ pretreatment and *Salmonella* Typhimurium-GFP (Stm-GFP) infection. Results are expressed as the proportion of *Salmonella* Typhimurium^high^ on Salmonella Typhi^+^. Representative results from 2–3 independent experiments. **(G)** CFU assays on PMA-differenciated THP1-Ф with and without IFN-γ pretreatment, following infection by *Mycobacterium abscessus*. All replicates from *n*=3 independent experiments are displayed. **(H)** Flow cytometry analyses on MDMs with and without IFN-γ pretreatment, following infection for 24 hours with *M. abscessus*-tdTomato.

We then studied cellular responses to IFN-γ in leukocytes from patients. IL-12p40 secretion into whole blood in response to BCG or BCG plus IFN-γ was in the control range for both patients (Figure S6A). By contrast, IL-12p70 induction in response to IFN-γ was impaired in both patients (Figure S6B). Impaired IL-12p70 induction probably contributed to the disseminated histoplasmosis observed in P1, as such infections are frequently reported in patients with complete IL-12Rβ1 or IL-12p40 deficiencies^12,19,22^. Patients with such deficiencies are also prone to isolated episodes of mycobacterial disease, but they almost never present recurrent episodes, contrasting with observations for P1 and P2. Impaired IL-12p70 production is, therefore, unlikely to explain the recurrence of mycobacterial disease in these two patients. Mononuclear myeloid cells are the best studied and classically defined effectors of IFN-γ-induced immunity^28^. They are also the only cells in which mycobacteria can replicate^71^. We therefore comprehensively profiled the transcriptome of mononuclear myeloid cells from patients after IFN-γ stimulation, by RNA-seq on iPSC-MΦ (P1) and MDMs (P2). After 8 hours of stimulation, 73 genes were found to display differential expression in the myeloid cells of both patients relative to controls, with all but one of these genes downregulated in the patients (Figures 6A, S6C–D, and Table S7). The expression of known MSMD genes was normal, except for *IL12RB1* and *ZNFX1*, which displayed mild downregulation (~4-fold). Conversely, the genes strongly expressed in controls but with much lower levels of expression (10- to 500-fold) in both patients included several genes known to be involved in cell-autonomous defense against intracellular pathogens, such as members of the *GBP* family^72,73^ (chr1p22), members of the *APOL* family (chr22q12, including *APOL3*^74^), *IDO1*^75^, and *RARRES3*^52,76^ (Figure 6A). The genes differentially expressed in the mononuclear myeloid cells of both patients were significantly enriched in (i) genes with an ISRE motif in their proximal promoter (Figure 6B) and (ii) loci to which IRF1 is known to bind, as detected by ChIP-seq in resting^77^ and IFN-γ-activated myeloid cells^58^ (Figure S6E). We confirmed the impairment of *GBP4* and *APOL3* induction by IFN-γ, by RT-qPCR on iPSC-MΦ derived from P1 and MDMs from P2 (Figure S6F). The impairment of APOL3, GBP1, IDO1, and RARRES3 protein production in response to IFN-γ stimulation was confirmed by western blotting on iPSC-MΦ from P1 and MDMs from P2 (Figure 6C). *ZNFX1* expression was normal (Figure 6C). The oxidative burst in response to stimulation with IFN-γ and/or PMA was reduced in MDMs from P2 (Figure 6D). We then analyzed IFN-γ-dependent immunity to intramacrophagic pathogens in IRF1-deficient myeloid cells. The pretreatment with IFN-γ of WT THP1 cells reduced the intracellular growth of *Salmonella* Typhimurium (Figures 6E–F and S6G) and *Mycobacterium abscessus* relative to non-stimulated conditions (Figure 6G). By contrast, IFN-γ had no effect in THP1 IRF1-knockout (KO) cells^78^ or in THP1 IFN-γR1^KO^ and STAT1^KO^ cells (Figures 6F–G and S6G). Similar findings were obtained for the MDMs of P2 for *M. abscessus* infection (Figures 6H and S6H). Together, these results suggest that human IRF1 governs IFN-γ-dependent macrophage activation and resistance to intracellular pathogens.

### Mildly impaired responses to IFN-α/β in IRF1-deficient cells

**Figure 7 –:**
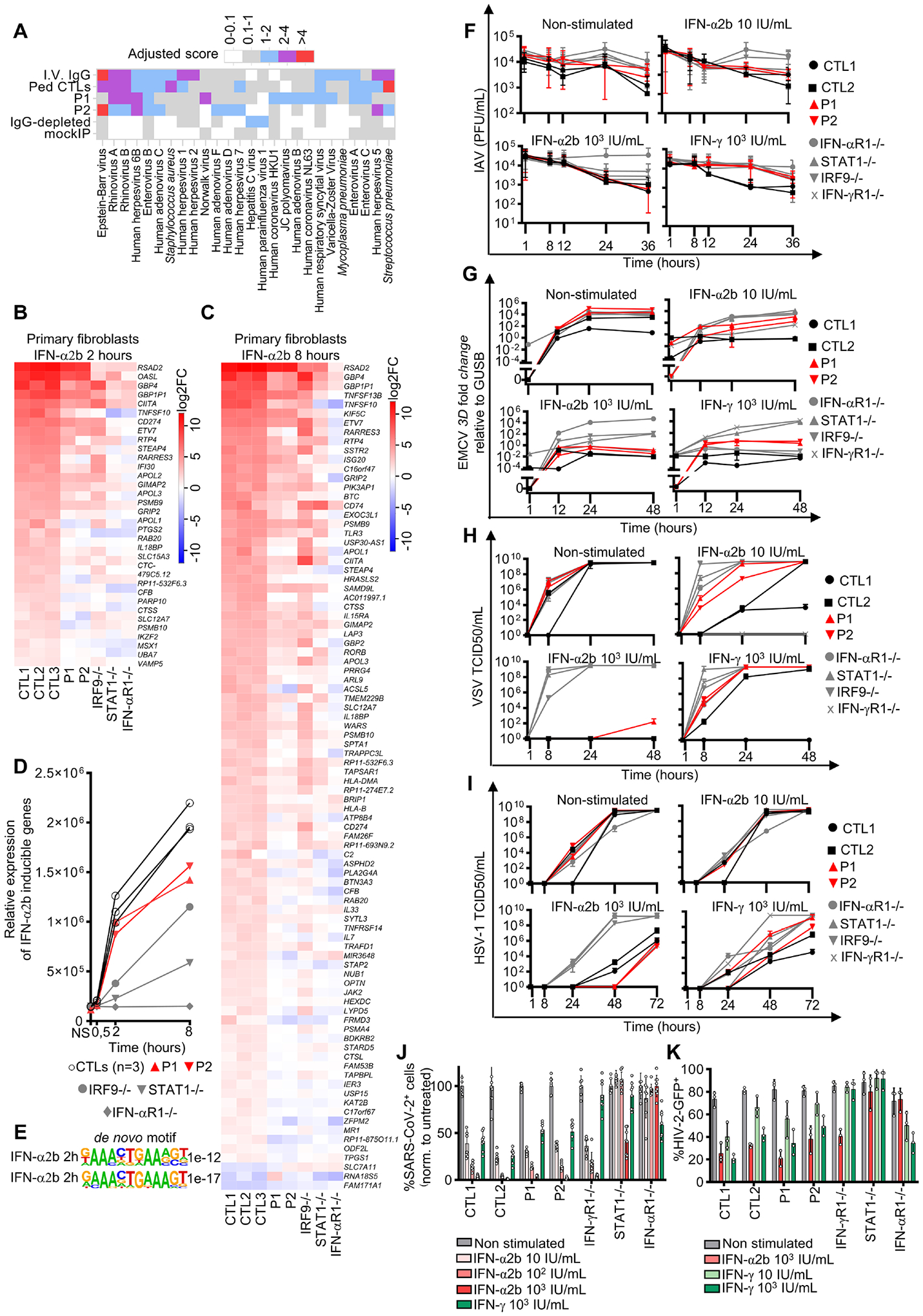
IFN-α and IFN-γ-driven antiviral immunity in the cells of IRF1-deficient patients. **(A)** Antiviral antibody responses to species for which at least one sample tested seropositive by PhIP-Seq. “IVIG” correspond to the mean response for samples from pooled patients on IVIGs and “pediatric CTLs” to pediatric controls. A hierarchical clustering of samples based on antiviral antibody levels is shown at the top. Heatmap showing genes differentially expressed (|log_2_(FC)| > 1 and adj. *p*-value < 0.05) in CTL cells after 2 hours **(B)** or 8 hours **(C)** of IFN-α2b stimulation, and differentially expressed in primary fibroblasts from P1 (|log_2_(FC)| > 1 and adj. *p*-value < 0.05) relative to CTLs. Genes differentially expressed in P1 and P2 relative to the control group at 2 and 8 hours from among those differentially expressed at both timepoints relative to non-stimulated fibroblasts in the control group, *i.e*., with a |log_2_(FC)| > 1 and adj. *p*-value < 0.05 after Benjamini-Hochberg correction in controls, and with a |log_2_(FC)| > 1 and adj. *p*-value < 0.05 after correction in patients relative to controls. **(D)** RNA-sequencing of IFN-α2b-inducible genes (logFC>2 in controls) in primary fibroblasts with and without IFN-α2b stimulation. **(E)** HOMER *de novo* motif analysis of genes differentially expressed in primary fibroblasts with IFN-α2b stimulation. **(F)** Influenza A virus (IAV), **(G)** encephalomyocarditis virus (EMCV), **(H)** vesicular stomatitis Indiana virus (VSV), **(I)** HSV-1, **(J)** SARS-CoV-2, and **(K)** HIV-2 infection of SV40-fibroblasts after pretreatment with IFN-α2b or IFN-γ. All viral infections were performed in 2–3 independent experiments.

In addition to its known role in IFN-γ-related immunity, the antiviral role of IRF1 has also been extensively studied. The ablation^52,61,79,80^ and overexpression^81,82^ of the *IRF1* gene have been shown to be associated with susceptibility and resistance to viral infections, respectively, *in vivo* in mice^83–87^ (Table S2) and *in vitro* in human cell lines^61,79,81^. Neither P1 nor P2 presented life-threatening viral infections (Supplemental Case report and Supplemental information). Phage immunoprecipitation-sequencing (PhIP-Seq) confirmed that both patients has been exposed to multiple DNA and RNA viruses (Figure 7A and Table S4). Stimulation with IFN-α/β induces IRF1, albeit at a level lower than observed after stimulation with IFN-γ^59–61,65,67^. We confirmed these findings in human cells and also found, surprisingly, that IFN-α/β-dependent IRF1 induction was ISGF3-independent (*i.e*. STAT2 and IRF9-independent) but GAS-dependent (*i.e*. STAT1-dependent) (Figures S7A–C). We analyzed antiviral IFN-α/β immunity in the patients’ cells by flow cytometry, RNA-seq, and in viral growth assays. The early response to IFN-α/β in SV40-fibroblasts and EBV-B cells, assessed by evaluating STAT1 phosphorylation after 20 minutes of stimulation with IFN-α2b and IFN-β, was normal in both patients (Figure S7D). We then studied the transcriptomic response of primary fibroblasts stimulated with IFN-α2b for 30 minutes, 2 hours, or 8 hours. We compared these responses to those of the cells of patients with complete deficiencies of IRF9, STAT1, or IFNAR1. Primary fibroblasts from healthy donors displayed differential expression relative to unstimulated conditions for 110 genes, 667 genes, and 1,093 genes for these three timepoints, respectively, with *IRF1* significantly upregulated at all these timepoints (Figures 7B–C, and Table S7). The impaired induction of various interferon-stimulated genes (ISGs; (for 2%, 10%, and 8% of the genes induced in controls)) was observed in the patients’ cells at these three timepoints. Quantitatively, 81% and 71% of the mRNA levels of IFN-α inducible genes in controls were correctly induced in both patients, at 2 and 8 hours, respectively (Figure 7D). The corresponding percentages were 24% and 54% in IRF9-deficient cells, and 8% and 23% in STAT1-deficient cells, for the same timepoints. Most of the ISGs dysregulated in IRF1-deficient cells have an ISRE motif in their promoters (Figure 7E). A subset of these ISGs also displayed impaired induction in IRF1-deficient cells but not in IRF9-deficient cells, defining these genes as GAF-IRF1-dependent (Figure 7E, and Table S7).

### Normal antiviral activity of IFN-α/β and IFN-γ in IRF1-deficient cells

We assessed the possible impact of these findings on the replication of several viruses in SV40-fibroblasts after pretreatment with IFN-α2b. In these conditions, the cells of the patients controlled the replication of encephalomyelitis virus (EMCV), influenza A virus (IAV), hepatitis A virus (HAV), herpes simplex virus type 1 (HSV-1), human immunodeficiency viruses type 1 and 2 (HIV-1 and HIV-2), yellow fever live-attenuated viral vaccine (YF17D-venus), SARS-CoV-2, and vesicular stomatitis virus Indiana (VSV) as effectively as control cells, whereas viral replication continued unabated in IFN-αR1^−/−^, IRF9^−/−^, STAT2^−/−^ or STAT1^−/−^ cells (Figures 7F–K and S7F–I), except for EMCV and VSV, for which greater susceptibility was observed with a low dose of IFN-α (Figure 7G–H). We conducted similar experiments with IFN-γ, which was initially described as an antiviral molecule^88–91^ before being identified as the macrophage-activating factor^28^. We found that IRF1-deficient cells had a lower susceptibility to infection following IFN-γ pretreatment, like control cells, for all viruses other than IAV, EMCV, and VSV, for which resistance was weaker than in control cells (Figures 7F–K and S7F–I). Collectively, these results suggest that the antiviral response of IRF1-deficient cells to IFN-α and IFN-γ is only mildly impaired and sufficient to protect IRF1-deficient fibroblasts against viral infection, consistent with the absence of severe viral illnesses in these two patients.

## Discussion

We report here that AR complete IRF1 deficiency is a genetic etiology of isolated and severe MSMD. The two unrelated IRF1-deficient patients of Latin American and Turkish ancestries described here experienced recurrent early-onset life-threatening mycobacterial diseases due to multiple mycobacteria (BCG, *M. avium*) despite treatment with multiple antimycobacterial drugs, and even treatment with recombinant IFN-γ in the case of P1. Remarkably, IRF1 deficiency was found to be as clinically severe as AR complete deficiencies of IFN-γ, IFN-γR1, or IFN-γR2^4,5,12^. Patients with these deficiencies display adverse reactions to BCG when vaccinated at birth, followed by relapses of BCG disease and/or disseminated disease caused by EM^4,5,12^. Disseminated infection with *M. avium* before six years of age is a hallmark of a lack of IFN-γ immunity due to inherited defects of the IFN-γ response pathway^4,5,12,34^, whereas *M. avium* disease at a later age may be due to profound deficiencies as a result of the production of anti-IFN-γ auto-antibodies^92,93^, or a severe progressive quantitative defect of IFN-γ myeloid target cells caused by GATA2 deficiency^94,95^. Given the severity of the clinical phenotype in these two unrelated patients, the clinical penetrance of inherited IRF1 deficiency for MSMD is probably complete. Consistently, *Irf1*^−/−^ mice are susceptible to both BCG^96^ and *M. tuberculosis*^97,98^, and other intramacrophagic pathogens^99–103^. Myeloid cells are classically considered to be the effector cells for IFN-γ^28^, and this cytokine is also their most potent activator^104^. Our findings indicate that IRF1 governs the response to IFN-γ, downstream from STAT1, in mononuclear myeloid cells. IRF1 controls the potent induction of several genes encoding intracellular components known to be effectors of resistance to intracellular pathogens, such as a protein from the GBP family^72–74,100,105,106^, IDO1^75^, RARRES3/PLAAT4^76^, and APOL3^74,107^. Our work extends the list of known IFN-γ-inducible IRF1-dependent genes in mice^58^ to human genes with no known ortholog in mice^108^, such as *RARRES3*^76^ and *APOL3*^74,107^. Inherited deficiencies of some of these IFN-γ- and IRF1-dependent effector genes may underlie MSMD or TB. IRF1 deficiency underlies severe and isolated MSMD, as in patients with AR IFN-γ, IFN-γR1, or IFN-γ2 deficiencies^4,5,12^ whose mononuclear myeloid cells cannot respond correctly to IFN-γ.

We also found additional quantitative and qualitative deficiencies of IFN-γ-producing lymphocytes in the two IRF1-deficient patients, consistent with reported findings for *Irf1*^−/−^ mice^83,101,109–113^. The observed deficiencies included an impairment of the development of NK cells, and naïve αβ CD8^+^ T cells, and impaired IFN-γ production by residual NK cells and γδ2^+^ T cells. However, the leukocytes of both patients produced normal total amounts of IFN-γ upon exposure to mycobacteria *in vitro*, suggesting that the abnormal counts or function of IFN-γ-producing lymphoid cell subsets in peripheral blood made only a marginal contribution to mycobacterial disease. IRF1 deficiency impairs the development of myeloid DCs, predominantly that of cDC1, the counts of which were low in the blood of both IRF1-deficient patients and in the tissues of *Irf1*^−/−^ mice. Our results suggest that IRF1 is essential for myeloid cell maturation, through an as yet unknown mechanism, possibly involving interaction with IRF8^25,27,114,115^. Indeed, human IRF8 governs DC development in a gene dosage-dependent manner, as illustrated by AD and AR IRF8 deficiencies^25,27,114–116^. We also documented low levels of IL-12p70 induction in both patients, which is more likely to result from direct binding to the *IL12A* locus, as previously shown in murine myeloid cells^117,118^, rather than a decrease in myeloid cell number. Consistently, patients with AD PU.1 deficiency, who have low circulating myeloid cell counts, have a normal capacity to produce IL-12^119^. Overall, in addition to their profoundly impaired cellular responses to IFN-γ, the patients with IRF1 deficiency have a mild impairment of IFN-γ production, which may have contributed to their MSMD.

By contrast, neither patient presented any severe viral diseases, despite low levels of ILCP, NK cells, and naïve CD8^+^ T cells. Patients with severe combined immunodeficiency (SCID) caused by LOF mutations of *IL2RG* or *JAK3* who undergo hematopoietic stem cell transplantation do not reconstitute a normal pool of peripheral ILC and NK cells^120^. They are not prone to viral infections other than HPV-driven common and flat warts, perhaps due to the persistence of deficiencies of keratinocytes or antigen-presenting cells in the skin^121^. Isolated deficiencies of ILCs and NK cells in patients with IEIs, such as GINS1, MCM4, or MCM10 deficiency^120,122–124^, can lead to various degrees of susceptibility to viral diseases, mostly caused by CMV. These IEIs are caused by genes with expression profiles not restricted to NK cells or ILCs, and the defect may be broader^120,122–124^. Patients with inherited CD8, TAP1, TAP2, TAPASIN, or β2-microglobulin deficiencies, all of whom have low levels of HLA-I in all cell types tested and low counts of blood CD8^+^ T cells, are not prone to viral infections either^125–131^. IEIs impairing CD8^+^ T-cell effector or expansion functions can underlie susceptibility to EBV^132^. P1 had not encountered CMV or EBV, but P2 had been exposed to CMV and had controlled its replication and tested positive for EBV by PCR. Overall, the low but non-zero counts of circulating antiviral lymphoid cell subsets in the two patients with IRF1 deficiency have so far proved sufficient to ensure immunity against the many viruses encountered in childhood. Innate and adaptive leukocytic immunity to viruses is evidently affected by complete IRF1 deficiency, albeit with no apparent clinical consequences, implying that other antiviral mechanisms can compensate for this relatively broad lymphoid deficiency.

More surprisingly, cell-intrinsic, IFN-α/β immunity is IRF1-independent for the nine viruses we have tested *in vitro*. The contrast between AR STAT1 and IRF1 deficiencies is striking in this respect, as the two patients with IRF1 deficiency did not suffer from the life-threatening viral diseases seen in patients with AR complete STAT1 deficiency^33,34^. Despite the impaired induction of a subset of target genes in fibroblasts stimulated with IFN-α/β, the patients displayed no severe viral diseases, even upon infection with SARS-CoV-2, probably the most potent known sensor of IFN-α/β deficiency^133–138^. Consistently, the cells of the patients controlled the replication of the seven RNA and DNA viruses tested *in vitro*, unlike cells from patients with AR STAT1, STAT2, IRF9, or IFNAR1 deficiency. All IEIs affecting the response to IFN-α/β underlying susceptibility to acute viral diseases (*i.e*. AR IFN-αR1, IFN-αR2, STAT1, STAT2, or IRF9 deficiencies) are caused by deficiencies leading to a complete abolition of ISGF3 function in response to stimulation with IFN-α/β^138–145^. These observations suggest that functional ISGF3 (composed of STAT1, STAT2, and IRF9) is essential for IFN-α/β intrinsic immunity, whereas IRF1 (formerly known as ISGF2^65,146^) is largely redundant for such immunity *in vivo*, as demonstrated by viral replication levels. We found that human IRF1 was driven by GAF but not ISGF3 complexes in response to IFN-α/β, but the GAF/STAT1-dependent induction of IRF1, and of subsequent target ISGs, such as GBP, RARRES3, APOL3, was not required for immunity to many viruses. By contrast, *Irf1*-deficient mice have been reported to be susceptible to a number of viral infections^147,83,84,87,86^. The lack of IRF1 in mice may, nevertheless, be partly compensated by other IRFs for antiviral immunity^148^. IRF1 is not induced after stimulation with IFN-λ^61^. In addition, humans with a complete deficiency of IL-10Rβ display a complete lack of response to IFN-λ and are apparently not susceptible to infectious diseases^149,150^, except fulminant viral hepatitis A^151^. Overall, the natural course of infectious diseases in the known patients with inherited deficiencies of IRF1 (this study), IRF3^152,153^, IRF7^138,140^, or IRF9^139,154^ suggests that IRF1 is essential for IFN-γ-dependent myeloid antimycobacterial immunity, whereas IRF3, IRF7 and IRF9 are essential for IFN-α/β-dependent antiviral immunity.

### Limitations of the Study

Our molecular, cellular, immunological, and clinical studies of two unrelated patients with inherited IRF1 deficiency do not exclude the possibility of predisposition to unusual and/or severe clinical diseases caused by viruses that have not been tested *in vitro*, or that have not been encountered by the patients *in vivo*, or both, or even such diseases caused by seemingly benign viruses in different infection conditions (*e.g*. high levels of inoculum, atypical route of infection, infection before vaccination), in these or other IRF1-deficient patients. Any susceptibility to viruses in these patients might be due to impaired IRF1-dependent IFN-α/β immunity, or due to the deficit of cytotoxic NK and CD8^+^ T cells, or both. In particular, the long-term outcome of infection with viruses capable of latency, such as those of the *Herpesviridae*, is unknown.

## STAR METHODS

### RESSOURCE AVAILABILITY

#### Lead Contact

Further information and requests for resources and reagents should be directed to and will be fulfilled by the Lead Contact, Jean-Laurent Casanova (casanova@mail.rockefeller.edu).

#### Materials Availability

All raw and processed data and biological materials, including immortalized cell lines from patients, are available upon request from the Lead Contact under a Material/Data Transfer Agreement with Inserm or the Rockefeller University.

#### Data and Code Availability

RNA-seq, single-cell RNA-seq, and CITE-seq data have been deposited at GEO and are publicly available as of the date of publication. Accession numbers are listed in the key resources table. The mass spectrometry proteomics data have been deposited with the ProteomeXchange Consortium *via* the PRIDE partner repository and datasets identifier is listed in the key resource table. Original western blot images, flow cytometry data, mass spectrometry data, and microscopy data reported in this paper will be shared by the lead contact upon request.

This paper does not report original code.

Any additional information required to reanalyze the data reported in this paper is available from the Lead Contact upon request.

### EXPERIMENT MODEL AND SUBJECT DETAILS

Patient 1 (P1, kindred A) is an eight-year-old girl born to consanguineous parents originating from and living in Argentina (Figure 1A). She was vaccinated with BCG at birth. At the age of five months, she was hospitalized for lymphadenitis and hepatosplenomegaly. BCG was detected in a lymph node biopsy and bone marrow aspirate by culture, attesting to disseminated BCG disease (BCG-osis). P1 was treated with a combination of amikacin, rifampicin, isoniazid, ethambutol, and levofloxacin for two years. At the age of three years, she had fever and abdominal pain. Computed tomography (CT) revealed hepatomegaly, and mediastinal and abdominal lymphadenopathies (Figure 1B). Mediastinal lymph node biopsy revealed granulomatous lymphadenitis (Figure 1C), and culture yielded *M. avium*. During this episode, PCR also detected *Histoplasma* sp. in the blood. P1 was treated with rifampicin, isoniazid, ethambutol, levofloxacin for two years for mycobacterial disease, and with amphotericin B followed by voriconazole for prophylaxis. At the age of four years, IFN-γ treatment was initiated following a suspected reactivation of mycobacterial disease. At the age of five years, P1 was hospitalized for pneumonia. *M. avium* was again identified in a sputum sample. Clarithromycin was added to the patient’s IFN-γ and antimycobacterial treatment. At the age of seven years, P1 had rhinitis causing upper respiratory obstruction. *M. intracellulare* subsp. *chimaera* was documented on a nasal swab. During this episode, P1 also had hepatosplenomegaly and pancytopenia. Bone marrow biopsy showed epithelioid granulomas with giant multinucleated cells. P1 was treated with liposomal amphotericin, levofloxacin, clarithromycin, and ethambutol. She is now seven years old and clinically well on immunoglobulins, recombinant IFN-γ, levofloxacin, clarithromycin, cotrimoxazole, and itraconazole.

Patient 2 (P2, kindred B) is a seven-year-old girl born to consanguineous parents originating from and living in Turkey (Figure 1A). She was vaccinated with BCG at two months of age. She presented with left axillary lymphadenitis at five months old. BCG was grown from the abscess, attesting to local-regional BCG-infection (BCG-itis). P2 was successfully treated with isoniazid, rifampicin, and ethambutol for six months. At the age of five years, she presented fever, digital clubbing, and rash. A chest computed tomography (CT)-scan revealed multiple mediastinal and hilar lymphadenopathies, and a lung mass (Figure 1D). Biopsy specimens from the lymphadenopathies and the lung mass contained multinucleated giant cells and spindle-shaped histiocytes (Figure 1E). *M. avium* was cultured from these lesions. P2 has since been treated with azithromycin, ethambutol, and rifabutin.

Mice used in this study (C57BL/6 (B6) mice and *Irf1*^−/−^ mice were all male.

### METHOD DETAILS

#### Patients

P1 is resident in Argentina and P2 is resident in Turkey. Informed consent was obtained in Argentina and Turkey, respectively, in accordance with local regulations and with institutional review board (IRB) approval. Experiments were conducted in Australia, Canada, France, Qatar, and the United States of America, in accordance with local regulations and with the approval of the IRB of the Rockefeller University and INSERM, for the United States of America and France, respectively. A detailed clinical case report is provided below. Healthy controls were recruited in Argentina, France, Turkey, and the United States of America.

#### Case reports for P1 and P2

The patient from kindred A (P1, II.1) is a girl born in 2014 and living in Tucuman, in the North East of Argentina (Figure 1A). She was vaccinated with BCG at birth. At the age of one month, she presented impetigo and a local abscess at the site of vaccination with BCG. At the age of five months, she was hospitalized with fever, hepatosplenomegaly, and intra-abdominal and mediastinal adenomegalies. *M. bovis*-BCG was cultured from lymph node and bone marrow aspiration fluids. P1 also had digestive symptoms, with diarrhea and intestinal subocclusion, and a cutaneous eruption. BCG-osis was successfully treated with a combination of amikacin, rifampicin, isoniazid, ethambutol, and levofloxacin. At the age of eight months, during hospitalization for intestinal malrotation surgery, P1 was found to have vesicular lesions on the scalp, trunk, perianal region, vulva, and lower limbs, but no signs of meningoencephalitis or pneumonia. The Tzanck test was positive, suggesting infection with varicella-zoster virus (VZV). Infection with this virus was confirmed by positive PhIP-Seq (Figure 7A) and serological testing for VZV (Table S4). Given that VZV was hospital-acquired in a young patient with a suspected IEI, and despite the absence of clinical signs of severity (including an absence of both pneumonia and encephalitis), P1 was pre-emptively treated with intravenous acyclovir, leading to resolution of the varicella virus infection. At the age of 11 months, P1 had a lower respiratory tract infection caused by parainfluenza virus 3 (PIV3), documented by PCR. She also experienced another lower respiratory tract infection caused by *Chlamydia pneumoniae*, and various other respiratory tract infections presumed to be viral. She did not require intubation for any of these respiratory tract infections. At the age of three years, she presented simultaneous disseminated infections due to *M. avium* and *Histoplasma capsulatum*. These infections were treated with amikacin, rifampicin, isoniazid, ethambutol, levofloxacin, and liposomal amphotericin B. During the episode of BCG-osis, P1 also displayed leukopenia, anemia, and thrombocytopenia. At the age of four years she presented with vesicular lesions of the right upper limb. However, PCR tests for VZV and HSV were negative. At the age of five years, P1 presented pneumonia due to *M. avium intracellulare*. Clarithromycin was added to the antimycobacterial treatment regimen. At the age of six years the patient suffered rhinosinusitis leading to nasal obstruction. *Acinetobacter baumanii* and *M. intracellulare* subsp. *chimaera* were documented on culture of a sinus sample. During this episode, the patient displayed hepatosplenomegaly and pancytopenia associated, on bone marrow biopsy, with epithelioid granuloma giant multinucleated cells without hemophagocytic lymphohistiocytosis (HLH). The patient was treated with liposomal amphotericin, levofloxacin, clarithromycin, and ethambutol. She suffered failure to thrive, from infancy (−2 to −3 SD for weight and height). Between the ages of two and four years, P1 suffered recurrent abdominal pain, with an enlarged liver and abdominal distention. Digestive tract endoscopy results were normal. Abdominal and pelvic CT-scan results were normal, with the exception of organomegaly. P1 had ventricular septal defects and café au lait spots on the skin, from birth. In addition to BCG, she was vaccinated against diphtheria, tetanus, hepatitis A, hepatitis B, *Haemophilus influenzae* type b, seasonal flu, pneumococcus (Prevenar 13), poliomyelitis (Salk), and rotavirus (Rotarix), and had received one dose of measles-mumps-rubella vaccine. Serological tests for HIV were negative. Chromosome 22q11.2 deletion syndrome was ruled out by fluorescence *in situ* hybridization. Blood karyotype was normal. The primary fibroblasts of P1 displayed normal sensitivity to mitomycin C and gave normal results in FANCD2-monoubiquitination assays^155,156^, ruling out Fanconi anemia. Plasma alpha-feto protein concentration was in the normal range. The oxidative burst in PNM cells (dihydrorhodamine test), and ADA and PNP activities in plasma were normal. A brain CT-scan at the age of four years was normal. P1 is currently being treated with subcutaneous recombinant IFN-γ (Imukin^®^), polyvalent subcutaneous immunoglobulin, levofloxacin, ethambutol, clarithromycin, trimethoprim-sulfamethoxazole, iron, folic acid, vitamin D and calcium. She is well on this treatment. The mother of P1 had no history of unusually severe infectious diseases. She did not display adverse reaction to BCG vaccination.

The patient from kindred B (P2, II.1) is a girl born in 2015 and living in Turkey (Figure 1A). She was vaccinated with BCG at birth, and presented left axillary lymphadenitis (BCG-itis) without organomegaly at the age of five months. She was successfully treated for six months with isoniazid, rifampicin, and ethambutol. At the age of one year, she presented respiratory infection due to influenza B virus that did not require oxygen support. At the age of five years, P2 was referred to hospital for intermittent fever and a rash of three months’ duration. She was treated with oral antibiotics for upper respiratory tract infection, and with colchicine for suspected familial Mediterranean fever. She had been on intramuscular antibiotics for two weeks when she was diagnosed with pneumonia, without identification of the causal microbe. On admission, she had tachypnea, but was in good general condition with no fever. Lymphadenopathies were observed in both axillary regions on clinical examination. A chest CT-scan revealed multiple lymphadenopathies in both axillary regions, in the mediastinal and hilar regions, and a mass-like lesion in the left lower lobe of the lungs. Abdominal CT-scan was normal, with no organomegaly. Antibiotic treatment with ceftriaxone and clindamycin was continued for pneumonia. A peripheral smear and bone marrow aspiration were performed; the results excluded leukemia and HLH. A left shift and leukocytosis with neutrophilia were found. Cultures of blood and nasopharyngeal fluid were negative. Three fasting gastric fluid aspiration specimens were sent for staining for acid-fast bacilli (AFB) and mycobacterial culture, but the results were negative on all three occasions. A tuberculin skin test (TST) gave a 17 mm induration, and the Quantiferon test was negative. Due to a lack of improvement, antituberculosis treatment was initiated with moxifloxacin, rifampicin, isoniazid, pyrazinamide and ethambutol. Samples of the lung mass and mediastinal lymphadenopathies were sent for histological analysis and mycobacterial culture. Cultures revealed *M. avium intracellulare*. The histopathology report showed a few giant cells and spindle-shaped histiocytes. P2 was treated with azithromycin, ethambutol, and rifabutin. The patient had papular hyperemic rashes on her hands and feet during clinical follow-up and was referred to a dermatologist. The cutaneous biopsy results obtained from the rashes were consistent with acute generalized exanthematous pustulosis. At the age of five years, the patient was infected with SARS-CoV-2, causing mild cold-like symptoms with no need for hospitalization. This patient has never been vaccinated against COVID-19. P2 is currently doing well. The father, mother, and brother of P2 had no history of unusually severe infectious diseases, including adverse effects of live vaccines.

#### Both *IRF1* variants are predicted to be deleterious

*IRF1* encodes interferon regulatory factor 1 protein (IRF1), a transcription factor induced by IFN-γ, the deficiency of which in mice underlies susceptibility to various pathogens, including mycobacteria^43,49,51,53,55,57,62,83,84,96–98,109,110,147^ (Table S2). IRF1 is a 325-amino acid transcription factor composed of an amino-terminal DNA-binding domain (DBD) that can bind to DNA interferon-stimulated response elements (ISRE) or positive regulatory domain I (PRDI) motifs^64^, an intermediate segment containing a putative nuclear localization sequence (NLS), and a carboxyterminal IRF association domain 2 (IAD2), which is crucial for transcriptional activity^65,66,68,70^ (Figure 1G). Both the c.385C>T and c.103C>T variants are predicted to be loss-of-function (pLOF). Indeed, both result in the creation of a premature stop codon, p.R129* and p.Q35*, respectively (Figure 1G). The CADD score of the two variants is 38, well above the 99% MSC^37,166^ of *IRF1* at 8.2 (Figure 1I). An analysis of public databases, including ExAC, gnomAD v2.1.1 or v3.1.1^168^, BRAVO/TOPmed freeze 8^170^, UK Biobank^172^, ATAV^174^, the Greater Middle East variome^176^, and the Turkish variome^178^ containing WES or whole-genome sequencing (WGS) data from more than 250,000 individuals showed that the p.Q35* variant is private, whereas p.R129* is found in the heterozygous state in two individuals. All the non-synonymous coding or structural variants of *IRF1* listed in public WES and WGS databases are present in the heterozygous state, with the exception of one homozygous missense variant (c.199G>C, p.A67P, rs137993322) in the TOPmed^170^ database with a gnomAD MAF of 1.2e-4 and a CADD score below that of p.R129* and p.Q35* (Figure 1I). We identified no other homozygous or compound heterozygous non-synonymous *IRF1* coding variants in our in-house database of 20,000 WES or WGS from patients with severe infectious diseases. The cumulative frequency of heterozygous pLOF variants in gnomAD is also low (4.3×10^−5^) (Figure 1I). Consistently, the consensus negative selection (CoNeS) score of *IRF1* is low, suggesting that this gene is under negative selection, consistent with either recessive or dominant inheritance^180^ (Figure S1D). These findings further suggest that homozygosity for the pLOF private *IRF1* variants identified here was causal for MSMD in P1 and P2.

#### Impaired development of lymphoid and myeloid cells in *Irf1*^−/−^ mice

IRF1-knockout mice (*Irf1*^−/−^) were first described in 1993^112^. Consistent with the results obtained for IRF1-deficient patients, *Irf1*^−/−^ mice with various genetic backgrounds (C57BL/6 and 129/Sv) display impaired development of NK cells, and T_H_1 cells, together with a predominance of naïve cells among CD8^+^ T cells (Table S5)^43,109,110,182,184^. However, other leukocyte subsets do not seem to have been analyzed in these mice. We studied the immunophenotypes of *Irf1*^−/−^ mice with a C57BL/6 background by isolating leukocytes from the bone marrow, spleen, and lung. We replicated the reported decrease in the absolute numbers of NK and CD8^+^ T cells in C57BL/6 *Irf1*^−/−^ mice (Figures S3E–F). The development of myeloid DCs has been little studied, and with only rudimentary markers, in mice, but the available results suggest an impaired maturation of myeloid DCs^186^, whereas ILC2 maturation has not been studied. We found that cDC1 counts were low in the spleen and lungs of *Irf1*^−/−^ mice, whereas cDC2 counts in the lungs of *Irf1*^−/−^ mice were unaffected, and those in the spleen were only slightly lower than those of WT mice (Figures S3E–F). Spleen pDC counts were normal in *Irf1*^−/−^ mice (Figure S3F). The number of mature ILC2 in the lungs was low, whereas the number of immature ILC2 progenitors in the bone marrow was normal in *Irf1*^−/−^ mice (Figure S3G). Overall, IRF1 deficiency appears to impede the development of the same lymphoid and myeloid cell subsets in humans and mice, resulting in deficiencies of cDC1, ILC2, NK cells, and CD8^+^ T cells.

#### Lack of severe viral infections in IRF1-deficient patients

In addition to its known role in IFN-γ-related immunity, the antiviral role of IRF1 has also been extensively studied. The ablation^52,61,79,80^ and overexpression^81,82^ of the *IRF1* gene have been shown to be associated with susceptibility and resistance to viral infections, respectively, *in vivo* in mice^83–87^ (Table S2) and *in vitro* in human cell lines^61,79,81^. Indeed, human IRF1 is induced by IFN-α/β^61,188^, but not by IFN-λ^61^. Inborn errors of IFN-α/β immunity underlie various severe viral diseases in humans^2,3,138–143,154,190,192^. However, neither P1 nor P2 presented life-threatening viral infections (see Supplemental Case report). The patients had experienced benign upper respiratory infections caused by *Parainfluenza virus* 3 (P1), influenza virus (P2), and SARS-CoV-2 (P2). The last of these infections is of particular importance as even subtle defects of type IFN-α/β immunity underlie critical COVID-19 pneumonia^3,134–138^. One of the major mechanisms of SARS-CoV-2 virulence depends on the ability of this virus to induce only small amounts of IFN-α/β, rendering the amounts of this cytokine produced all the more important to the infected individual^3,194,196^. Phage immunoprecipitation-sequencing (PhIP-Seq) confirmed that both patients has been exposed to multiple DNA and RNA viruses (Figure 7A and Table S4). Thus, both these patients with IRF1 deficiency controlled many common, and even virulent viruses well.

#### Genetics

P1 and P2 were genotyped from DNA extracted from whole blood, with the Genome-Wide Human SNP Array 6.0 and/or WES with SureSelect Human All Exon V6 from Agilent. *IRF1* exon 3 was amplified from genomic DNA with the following primers (5’-TGGTCTGTTTAAGCCAGCCTC-3’ and 5’-CAGAAACACAAGTCTGCCACC-3’), and exon 5 with the following primers (5’-TTCCACCTCTCACCAAGAACC-3’ and 5’-CAGAGAAGGTATCAGGGCTGG-3’) both at a Tm of 60°C, with the GoTaq DNA Polymerase (#M3005, Promega). Amplicons were then sequenced by the Sanger sequencing method with Big Dye Terminator v3.1 (Thermo Fisher Scientific), and subjected to capillary electrophoresis (#A30469, Applied Biosystems 3500xL system, Thermo Fisher Scientific). The genotype of P1 was checked with DNA extracted from whole blood, granulocytes, EBV-B cells, HVS-T cells, SV40-fibroblasts, primary fibroblasts, and induced pluripotent stem cells (iPSCs). The genotype of her mother was checked with DNA extracted from whole blood and granulocytes. The genotype of P2 was checked with DNA extracted from whole blood, granulocytes, primary fibroblasts, SV40-fibroblasts and MDMs, and the genotypes of her relatives were checked with DNA extracted from whole blood.

#### Cell culture and stimulation

HEK293T cells and SV40-fibroblasts were cultured in Dulbecco/Vogt modified Eagle’s minimal essential medium (DMEM, #61965059, Gibco) supplemented with decomplemented 10% fetal bovine serum (FBS, #10270098, Gibco), and EBV-B cells were cultured in Roswell Park Memorial Institute medium (RPMI 1640, # 61870044, Gibco) supplemented with 10% decomplemented FBS. HVS-T cells were cultured with an equal mixture of RPMI and Panserin 401 (#P04–710401, Pan Biotech) supplemented with 10% decomplemented FBS, 1.2% Glutamax (#35050061, Gibco), gentamycin, and 100 IU/mL recombinant interleukin 2 (Aldesleukin, Novartis). T-cell blasts were cultured in ImmunoCult-XF T Cell Exp Medium (#10981, Stemcell) in the presence of IL-2 and primed every two weeks with ImmunoCult Human CD3/CD28/CD2 T-Cell Activator (#10970, Stemcell). All cells were grown at 37°C, under an atmosphere containing 5% CO_2_. HEK293T cells were plated at a density of 600,000 cells per well, in six-well plates. For CD274 (PD-L1) induction, SV40-fibroblasts were plated at a density of 200,000 cells per well, in six-well plates, with 2 mL DMEM-10% FBS per well, and were left unstimulated or were stimulated the following day with 10^2^, 10^3^, or 10^4^ IU/mL recombinant IFN-γ (IFN-γ, Imukin, Horizon Pharma). Forty-eight hours after stimulation, cells were harvested with trypsin, and stained as described below. For p-STAT1 induction, SV40-fibroblasts were starved overnight in DMEM-1% FBS medium, and were left unstimulated or were stimulated for 20 minutes with 10^3^ IU/mL recombinant IFN-γ (Imukin, Horizon Pharma), IFN-2αb (Introna, MSD), or 1 ng/mL IFN-β (Miltenyi Biotec). The same protocol was used for p-STAT1 induction in EBV-B cells, except that the cells were starved in RPMI-1% FBS medium and stimulated with 10^5^ IU/mL IFN-γ or IFN-α2b, or 10 ng/mL IFN-β (Miltenyi Biotec). The reaction was stopped by adding cold PBS, and the cells were harvested with trypsin and directly stained, as described below.

#### Site-directed mutagenesis, transient and stable transfection

Empty vector (EV) and a plasmid containing the DDK-tagged *IRF1* cDNA were obtained from a commercial source (#RCPS100001 and #RC203500, respectively, Origene). Constructs carrying single-nucleotide mutant alleles were generated from this plasmid by mutagenesis with appropriate primers, with the Pfu Ultra II Fusion HS DNA (#600674, Agilent) polymerase, followed by digestion with *Dpn*I (#R0176L, New England Biolab). For assessments of the reinitiation of translation, methionine codons were mutated to alanine codons (ATG>GCG). Plasmids were amplified in competent *E. coli* cells (#C3019H, New England Biolab) and purified with a maxiprep kit (#12663, Qiagen). HEK293T cells were transiently transfected with the various constructs at a concentration of 2.5 μg/mL, with the Lipofectamine LTX kit (#15338100, Thermo Fisher Scientific) in accordance with the manufacturer’s instructions. Retroviral plasmids and vectors were prepared as previously described, with primers for site-directed mutagenesis or deletion, and were produced in Phoenix cells^157^.

#### Western blotting and EMSA

Total protein extracts were prepared by mixing cells with modified radioimmunoprecipitation assay buffer supplemented with protease inhibitors (EDTA-free Complete, Roche) and phosphatase inhibitor cocktail (PhosphoStop, Roche), 0.1 mM dithiothreitol (DTT; Life Technologies), 10^−3^ mM Na_3_VO_4_, and 1 mM PMSF, and incubating for 40 minutes on ice. The cytoplasmic and nuclear contents of the cells were separated with NE-PER nuclear and cytoplasmic extraction reagents (#78835, Thermo Fisher Scientific). Equal amounts of protein, according to a Bradford protein assay (#5000002, Biorad), were resolved by SDS-PAGE in a Criterion TGX 10% or 12% precast gel (Biorad) and the bands obtained were transferred to a nitrocellulose membrane (#1704159 and #1704157, Biorad). Membranes were probed with antibodies directed against IRF1 (unconjugated, clone D5E4, #8478, Cell Signaling; or unconjugated, polyclonal, #11335–1-AP, Proteintech), IRF8 (unconjugated, goat polyclonal, #sc-6058, Santa Cruz; or unconjugated, clone D20D8, #5628, Cell Signaling), IRF9 (unconjugated, rabbit polyclonal, #sc-496, Santa Cruz; or unconjugated, rabbit polyclonal, #14167–1-AP, ProteinTech), IRF3 (unconjugated, clone D9J5Q, #10949, Cell Signaling), STAT1 (unconjugated, clone 1, #610115, Beckton-Dickinson), pSTAT1 (unconjugated, clone 4a, #612232, Beckton-Dickinson), STAT2 (unconjugated, clone B-3, #sc-514193, Santa Cruz), MX1 (unconjugated, polyclonal, #13750–1-AP, ProteinTech), ISG15 (HRP-conjugated, clone F-9, sc-166755, Santa-Cruz), DDK-tag (HRP-conjugated, clone M2, #A8592, Sigma-Aldrich), GBP1 (unconjugated, clone 1B1, #sc-53857, Santa-Cruz, #A8592, Sigma-Aldrich), APOL3 (unconjugated, clone EPR8238(2), #ab154869, Abcam), RARRES3 (unconjugated, rabbit polyclonal, #12065–1-AP, ProteinTech), vinculin (unconjugated, clone EPR8185, #ab129002, Abcam; or HRP-conjugated, clone 7F9, #sc-73614-HRP, Santa Cruz), and lamin A/C (HRP-conjugated, clone E-1, #sc-376248-HRP, Santa Cruz). Unconjugated antibodies were detected by incubation with goat anti-mouse or rabbit IgG (H + L)-HRP-conjugated antibodies (#1706516 or #1706515, respectively, Biorad). Binding was detected by incubation with the Clarity Western ECL substrate (Biorad, #1705061) or SuperSignal West Femto (Thermo Fisher Scientific, #34096) with ChemiDoc MP (Biorad). The Spectra Multicolor Broad Range Protein Ladder (#26623, Thermo Fisher Scientific) or the Chameleon Duo Prestained Protein Ladder (#928–6000, Licor) was used to provide molecular weight markers. Membranes were stripped in Restore Western Blot Stripping Buffer (#21063, Thermo Fisher Scientific).

EMSA was performed by incubating 10 μg of nuclear protein lysate on ice for 30 minutes with an IRD700-conjugated ISRE probe (5′-GATCGGGAAAGGGAAACCGAAACTGAA-3’) designed on the basis of the ISRE motif from the *ISG15* promoter. For supershift assays, nuclear protein lysates were incubated for 30 minutes on ice with 3 μg anti-DDK antibody (clone M2, #14793S, Cell Signaling) or the corresponding isotype (#2729S, Cell Signaling). Protein/oligonucleotide mixtures were then subjected to electrophoresis in 12.5% acrylamide/bis-acrylamide 37.5:1 gels in 0.5% TBE migration buffer for ~120 minutes at 200 mA and 4°C in the dark. Binding was detected with the Licor Odyssey CLx system (Li-Cor, Lincoln). Images were analyzed with Imagine Lab 6.0.1 build 34 (Bio-Rad Laboratories).

#### Confocal microscopy

SV40-fibroblasts were plated on chambered coverslips (#80826, iBidi). The following day, they were left unstimulated or were stimulated for the indicated times with 10^3^ IU/mL IFN-γ (Imukin, Horizon Pharma). Cells were fixed by incubation for 15 minutes in 4% formaldehyde in phosphate-buffered saline (PBS), pH 7.4 at 37°C. The cells were then incubated overnight at 4°C with primary antibody (unconjugated, clone D5E4, #8478, Cell Signaling). They were washed three times in PBS, stained by incubation with secondary antibodies for one hour at room temperature (goat anti-rabbit IgG Alexa Fluor 555, #A21429), and left in ProLong Gold with DAPI (#P36931, Thermo Fisher Scientific). Cells were then visualized by confocal microscopy (×63 oil immersion lens, SP8 gSTED, Leica). Images were analyzed with Fiji software.

#### Luciferase assay

We used two different ISRE-luciferase reporter assays: (i) an assay using the ISRE3 reporter plasmid (pGL4.10[luc2] backbone, Promega #E6651), which contains, as previously described^158^, three repeats of the ISRE sequence (5’-GGAAAGGGAAACCGAAACTGAA-3’) separated by spacers designed on the basis of the ISRE motif from the *ISG15* promoter, (ii) an assay using the ISRE5 reporter plasmid, which contains, as previously described^139^, five repeats of the ISRE sequence 5’-GGGAAAGTGAAACTA-3’. HEK293T cells were transiently transfected, in 96-well plates, with the (ISRE) reporter plasmid (100 ng/well and 100 μL DMEM-10% FBS medium), the pRL-SV40 vector (Promega, #E2231, 40 ng/well) and the *IRF1* WT or mutant p.CMV6 plasmid (100 ng/well), with the Lipofectamine LTX kit (Thermo Fisher Scientific, #15338–100), according to the manufacturer’s instructions. Cells were used for the ISRE assay with the Dual-Luciferase system kit (Promega #E1980), according to the manufacturer’s protocol, 24 hours after transfection. Signal intensity was determined with a Victor X4 plate reader (Perkin Elmer). Experiments were performed in triplicate, and dual reporter activity is expressed as the fold-induction relative to cells transfected with the empty vector.

#### RT-qPCR and cDNA

RNA was extracted with the RNeasy Plus Mini Kit (#74136, Qiagen) or Quick-RNA MicroPrep Kit (#R1051, Zymo). Any remaining genomic DNA was removed by extraction on a column or by DNase digestion. RNA was reverse-transcribed with the SuperScript II Reverse Transcriptase (#18064014, Thermo Fisher Scientific) and oligo(dT)_12–18_ (#18418012, Thermo Fisher Scientific) or with the High-Capacity RNA-to-cDNA Kit (#4387406, Applied Biosystems), according to the manufacturer’s protocol. qPCR was performed on cDNA with *Taq*Man Fast Universal PCR Master Mix (2X), no AmpErase UNG (#4352042, Thermo Fisher Scientific) on a 7500 Real-Time PCR System (Applied Biosystems) or *Taq*man ViiA7, with the following probes, all from Thermo Fisher Scientific: *IRF1* exons 3–4 (#Hs00971960_m1), *IRF1* exons 8–9 (#Hs00971965_m1), *GBP4* (#Hs00364728_m1), *APOL3* (#Hs00758274_m1), and *GUSB* (#1702016).

#### Flow cytometry on cell lines

SV40-fibroblasts were incubated with the PE-Dazzle-594-PD-L1 (CD274) antibody (clone 29E.2A3, #329732, BioLegend), or the corresponding isotype (#400358, BioLegend). For pSTAT1 staining, the cells were starved overnight in DMEM-1% FBS. For intracellular staining, 10^6^ cells were washed with PBS-2% FBS- 2 mM EDTA buffer, fixed by incubation for 10 minutes at 37°C with Fix Buffer I (#557870, Beckton Dickinson,) and permeabilized by incubation for 20 minutes at 4°C with Phosflow Perm Buffer III (#558050, Beckton Dickinson). Cells were then incubated with PE-coupled STAT1 (clone 1, #558537, Beckton Dickinson) or PE- or AF647-conjugated anti-pSTAT1 antibody (clone 4a, # 612564 or #612597, Beckton Dickinson), the corresponding isotype (#554680 and #565363, respectively, Beckton Dickinson), or unconjugated IRF1 (clone D5E4, #8478, Cell Signaling) or the corresponding isotype, for detection with PE-conjugated goat anti-rabbit antibody (#A10542, Thermo Fisher Scientific). All non-fibroblastic cells were also stained with the Aqua Dead Cell Stain kit (#L34957, Thermo Fisher Scientific). Cells were acquired on a Beckman Coulter Gallios flow cytometer and analyzed with FlowJo Software.

#### Mass spectrometry on primary fibroblasts

Primary fibroblasts were plated at a density of 180,000 cells/mL in 12 mL DMEM supplemented with 10% FCS in T75 flasks. The following day, they were starved overnight and incubated for 24 hours with or without 10^3^ IU/mL IFN-γ (Imukin, Horizon Pharma). The following day, cells were harvested with trypsin, washed once in PBS, and total protein was extracted by incubation for 40 minutes on ice (with vortexing for 15 seconds every 10 minutes) in the following SDS-free RIPA buffer: 150 mM NaCl, 1% NP40 (#85124, Thermo Fisher Scientific), 50 mM Tris, 1 mM EDTA, and EDTA-free protease inhibitor (#0469332001, Roche). A volume of 80 to 120 μL buffer was used, depending on the size of the pellet. The mixture was then centrifuged at 16,800 *g* for 10 minutes at 4°C and the protein-containing supernatant was collected. The concentration of protein in the supernatant was determined with the Pierce BCA Protein Assay Kit (#23225, Thermo Fisher Scientific) and the standard test tube protocol. Protein integrity was assessed with a colloidal blue staining kit (#LC6025, Thermo Fisher Scientific). Samples were then stored at −80°C before further processing.

The cysteine residues were then reduced and alkylated with DTT and IAA. Proteins were precipitated in ice-cold acetone and the resulting pellet was dissolved in 200 mM EPPs pH 8.5 supplemented with 1 μg sequencing grade trypsin. Samples were digested by incubation overnight at room temperature. An additional 1 μg of trypsin was then added and the temperature was increased to 50°C for 1 hour. Peptides were labeled by incubation with TMTpro reagent for 1 hour at room temperature. Stoichiometry and labeling efficiency were checked, hydroxylamine was added to quench the reaction and the peptides were pooled. TMTpro peptides were first separated into two fractions by custom-made SCX column. Each of these two fractions was then further separated into eight fractions on high-pH reversed-phase spin columns, and the eight fractions were then concatenated to obtain a total of 12 fractions. The peptide fractions were separated by HPLC for two hours with a linear gradient on a 250 mm*75 μm EasySpray column connected to an Easy nLC 1200 HPLC machine, with an orbitrap Fusion Lumos mass spectrometer operating in DDA MS2 positive mode used for analysis. Spectra were queried against the human proteome (downloaded from uniprot.org on 2019/02/12) at a FDR of 1% with Sequest HT through Proteome Discoverer v. 2.5. A spectral purity of 75% was required to ensure adequate confidence in quantification. Protein abundances were processed further in the Perseus statistical software environment. Abundances were log_2_-transformed and normalized against the median intensity for each sample. The mass spectrometry proteomics data have been deposited with the ProteomeXchange Consortium *via* the PRIDE^159^ partner repository with the dataset identifier PXD037759.

#### Deep flow cytometry phenotyping and *ex vivo* naïve CD4^+^ T-cell polarization experiments

Cryopreserved PBMCs and their subpopulations were analyzed with a 28-color flow cytometry panel, as previously described^160^. PBMCs were collected from P1 at the age of five years and from P2 at the age of six years. Both patients were receiving broad-spectrum antimycobacterial drugs and P1 was also receiving recombinant IFN-γ. Cells were also labeled with anti-CD4 (APC-Cy7, RPA-T4, #557871, BD Pharmingen), anti-CD45RA (BV605, HI100, #562886, BD Horizon), and anti-CCR7 (AF700, 100503, #561143, BD Pharmingen) antibodies, and naïve (defined as CD45RA^+^CCR7^+^CD4^+^) T cells were isolated (>98% purity) with a FACS Aria cell sorter (BD Biosciences). Isolated cells were then cultured with T-cell activation and expansion beads (TAE; anti-CD2/CD3/CD28; Miltenyi Biotec) + IL-2 (50 IU/mL, Millipore) to allow proliferation to occur, over a period of seven days. The cells were then subcultured with TAE beads alone (T_H_0) or under T_H_1 (IL-12 [50 ng/mL; R&D Systems]), T_H_2 (IL-4 [1 IU/mL; Thermo Fisher Scientific], T_H_9 ([100 IU/mL; Thermo Fisher Scientific], TGF-β [2.5 ng/mL; R&D Systems]), or T_H_17 (TGF-β [2.5 ng/mL; R&D Systems], IL-1β [50 ng/mL; Peprotech], IL-6 [50 ng/mL; PeproTech], IL-21 [50 ng/mL; PeproTech], IL-23 [50 ng/mL; Thermo Fisher Scientific]) polarizing conditions. After five days of culture, the supernatant was used for assessments of the secretion of IL-2, IL-4, IL-5, IL-6, IL-9, IL-10, IL-13, IL-17A, IL-17F, IFN-γ, and TNF with a cytometric bead array (BD Biosciences). Once the supernatant had been collected, the cells were stimulated with PMA (100 ng/mL)-ionomycin (750 ng/mL) for six hours, with brefeldin A (10 μg/mL) added after the first two hours of incubation. For the assessment of intracellular cytokine production, cells were stained for IFN-γ (BUV737, 4S.B3, #564620, BD Horizon), TNF (PerCP, Mab11, #502924, BioLegend), IL-9 (PE, MH9A3, #560807, BD Pharmingen), IL-13 (BV421, JES10–5A2, #563580, BD Horizon), IL-4 (AF488, 8D4–8, #500710, BioLegend), IL-17A (BV510, BL168, #512330, BioLegend), IL-17F (BV650, O33–782, #562264, BD Horizon), IL-2 (BV750, MQ1–17H12, #566361, BD Horizon), and IL-21 (eF660, eBio3A3-N2, #50-7219-42, Thermo Fisher Scientific), and analyzed by FACS (BD FACSymphony High-Speed Cell Analyzer A3). A previously described gating strategy was used^160^.

#### Immunophenotyping of MAIT, iNKT, and γδ T cells

The immunophenotyping of MAIT, iNKT, and γδ T cells was performed as previously described^8^ on cryopreserved PBMCs from P1 prepared from a sample collected at the age of three years; and as previously described^10^ on cryopreserved PBMCs from P2 prepared from a sample collected at the age of six years. Both patients were receiving broad-spectrum antimycobacterial drugs. Briefly, staining was performed in the presence of Fcblock (Miltenyi Biotec), with Zombie-NIR live-dead exclusion dye (#423105, BioLegend), anti-CD3-Alexa532 (Clone UCHT1, # 58-0038-42, Thermo Fisher Scientific), anti-γδTCR-FITC (#11-9959-42, Thermo Fisher Scientific), anti-Vδ2-APC-Fire750 (#331420, BioLegend), anti-CD56-BV605 (clone 5.1H11, #362538, BioLegend), anti-CD4-BV750 (#5663656, BD Biosciences), anti-CD8a-BV510 (clone RPA-T8, #301047, BioLegend), anti-Vα7.2-BV711 (clone 3C10, #351731, BioLegend), anti-Vα24-Jα18-PE-Cy7 (clone 6B11, #342912, BioLegend), anti-Vδ1-Vioblue (#30-100-555, Miltenyi Biotec), anti-CD161-PE (clone HP-3G10, #339938, BioLegend) and anti-Vβ11-APC (Miltenyi Biotec) antibodies. Cells were analyzed with an Aurora cytometer (Cytek). The gating strategy for MAIT cells, iNKT cells, γδ1^+^ T cells, and γδ2^+^ T cells has been described elsewhere^8,69^.

#### ILC immunophenotyping

ILC, T, B, and NK cells were immunophenotyped on cryopreserved PBMCs from P1 and P2 prepared from samples collected at the ages of four and six years, respectively. Both patients were receiving broad-spectrum antimycobacterial drugs and P1 was also receiving recombinant IFN-γ. Briefly, biotinylated anti-human CD1a (biotin, HI149, #300112, BioLegend), CD14 (biotin, 61D3, #13–0149-82, Invitrogen), CD34 (biotin, 4H11, #316404, BioLegend), CD123 (biotin, 6H6, #306004, BioLegend), CD203c (biotin, FR3–16A11, #130-092-345, Miltenyi Biotec), CD303 (biotin, AC144, #130-090-691, Miltenyi Biotec), FcεRIα (biotin, AER-37(CRA-1), #334606, BioLegend), TCRαβ (biotin, IP26, #306704, BioLegend) and TCRγδ (biotin, B1, #555716, BD Biosciences) antibodies were used, in combination with streptavidin BUV661 (#565081, BD Biosciences), for lineage staining, along with anti-human CD4 FITC (OKT4, #317408, BioLegend), CD336 PerCP-eFluor710 (NKp44, 44.189, #46-3369-42), EOMES PE (WD1928, #12-4877-42, Invitrogen), CD8a PE-CF594 (RPA-T8, #562282, BD Biosciences), CD127 PE-Cy7 (eBioRDR5, #25-1278-42, Invitrogen), CD294 (CRTh2) AF647 (BM16, #558042, BD Biosciences), CD161 AF700 (HP-3G10, #339942, BD Biosciences), CD94 APC-Fire750 (DX22, #305518, BioLegend), CD335 (NKp46) BV421 (9E2/NKp46, #564065, BD Biosciences), CD45RA BV570 (HI100, #304132, BioLegend), CD117 BV605 (104D2, #313218, BioLegend), CD3 BV650 (UCHT1, #563852, BD Biosciences), CD7 BV711 (M-T701, #564018, BD Biosciences), T-bet BV786 (O4-46, #564141, BD Biosciences), CD19 BUV395 (SJ25C1, #563549, BD Biosciences), CD16 BUV496 (3G8, #564653, BD Biosciences), CD25 BUV563 (2A3, #565699, BD Biosciences), CD56 BUV737 (NCAM16.2, #564447, BD Biosciences) and CD45 BUV805 (HI30, #612891, BD Biosciences) antibodies. Human IgG from serum (Sigma-Aldrich) was used to block Fc receptors before staining. Extracellular staining was performed in Brilliant Stain Buffer (BD Biosciences). Dead cells were excluded with the fixable viability dye eFluor 506 (Invitrogen). Transcription factors were stained with the FoxP3 staining buffer set (Invitrogen), in accordance with the manufacturer’s instructions. Samples were acquired on a Symphony A5 cytometer (BD Biosciences) with FACSDiva 8 software and were analyzed with FlowJo v.10 (BD Biosciences).

#### Mass cytometry on fresh whole blood and cryopreserved PBMCs

Whole-blood mass cytometry was performed with two different panels, on two different blood samples from P2 collected two months apart, at the age of six years. P2 was receiving broad-spectrum antimycobacterial drugs. The first panel was that of the Maxpar Direct Immune Profiling Assay (#201325, Fluidigm), used according to the manufacturer’s instructions. The second was a custom-produced panel, the content of which is detailed in STAR Methods. Marked cells were frozen at −80°C after overnight dead-cell staining, and acquisition was performed on a Helios machine (Fluidigm). All the samples were processed within 24 hours of sampling. Data analysis was performed with OMIQ software. Mass cytometry on cryopreserved PBMCs was performed as previously described^8^ on two different samples collected at the ages of three and five years. The antibodies indicated in STAR Methods were used. The gating strategy was as previously described^8^.

#### Whole-blood activation ELISA for cytokines

Venous blood samples from healthy controls, P1 (aged 3 years), and P2 (aged 6 years) were collected in tubes containing heparin^161,162^. Both patients were receiving broad-spectrum antimycobacterial drugs. These samples were diluted 1:2 in RPMI 1640 (Gibco) supplemented with 100 IU/mL penicillin and 100 μg/mL streptomycin (Gibco). We then dispensed 1 mL of each diluted blood sample into each of five wells (1 mL/well) of a 48-well plate (Nunc). These samples were incubated for 48 hours at 37°C, under an atmosphere containing 5% CO_2_/95% air, and under various activation conditions: with medium alone, with live BCG (*M. bovis*-BCG, Pasteur substrain) at a MOI of 20 BCG cells/leukocyte, or with BCG plus recombinant (rh) IL-12 (20 ng/mL; R&D Systems), or BCG plus IFN-γ (Imukin, Horizon Pharma). The supernatants were then collected and subjected to ELISA.

#### ELISA

Supernatants from whole-blood stimulation experiments were used for determinations of IL-12p40 (#DP400, R&D Systems), IL-12p70 (#HS120, R&D Systems), and IFN-γ (#DIF50, R&D Systems), in accordance with the manufacturer’s protocol.

#### Phage immunoprecipitation-sequencing (PhIP-Seq)

A plasma sample was collected from P1 at the age of three years (before immunoglobulin administration) and from P2 at the age of six years. For antibody profiling by phage immunoprecipitation-sequencing (PhIP-Seq)^163^, plasma samples from both patients and controls were assayed and data were analyzed as previously described^5,164^, but with the following modifications. We calculated species-specific significance cutoff values to estimate the minimum number of enriched, non-homologous peptides required to consider a sample seropositive (as previously described^163^) with an in-house dataset and a generalized linear model. For each sample, we calculated virus-specific scores by dividing the counts of enriched, non-homologous peptides by the estimated cutoff score. These adjusted virus scores were used for the heatmap plot (Figure 7A). In addition to studying the patients reported here, we also calculated and plotted the mean antibody responses for a pediatric control cohort of lean individuals without infectious or immunological disease (*n*=111; age range: 7 to 15 years; median age: 11.0 years) described in a previous study^165,167^. Pooled human plasma for IVIg (Privigen^®^ CSL Behring AG) and human IgG-depleted serum (Molecular Innovations, Inc.) served as additional controls. All research on human subjects was performed after informed written consent had been obtained or after the samples had been rendered anonymous. The procedures were approved by the Institutional Research Ethics Boards of Sidra Medicine.

#### Viral infection experiments

In viral infection experiments (as indicated), SV40-fibroblasts were subjected to pretreatment for 16 hours with the indicated doses of IFN-α2b (Introna, MSD) or IFN-γ (Imukin, Horizon Pharma).

VSV (Indiana strain) replication experiments were performed as previously described^143^. Briefly, 1.25 × 10^5^ SV40-fibroblasts per well were plated in 24-well plates, in DMEM supplemented with 10% FBS. Cells were infected with VSV at a multiplicity of infection (MOI) of 0.1, in DMEM supplemented with 2% FBS. Cells and supernatants were obtained at various time points (1, 8, 24 and 48 hours) and frozen. VSV titers were determined by calculating the 50% end point (TCID_50_), as described by Reed and Muench^169^, following the inoculation of Vero cell cultures in 96-well plates.

For HIV infections, the HIV-1 reporter virus HIV-GFP env-nef- was NL4–3 ΔenvΔnef encoding GFP in nef^171^ and the HIV-2 reporter virus was ROD9 ΔenvΔnef encoding GFP in nef^173^. Viral particles were produced by transfecting 293FT cells in six-well plates with 3 μg DNA and 8 μL TransIT-293 (Mirus Bio) per well: for VSV-G-pseudotyped HIV-1, 0.2 μg CMV-VSVG, 0.2 μg HXB2^175^ and 2.6 μg HIV-GFP env-nef-; for VSV-G-pseudotyped HIV-2, 0.4 μg CMV-VSVG and 2.6 μg HIV-2ROD9ΔenvΔnef GFP. One day after transfection, the medium was removed, and fresh medium was added. Viral supernatants were harvested the following day, passed through a filter with 0.45 μm pores, and frozen at −80°C. SV40-fibroblasts were plated in a 96-well plate at a density of 2.5 × 10^4^/mL in 200 μL of DMEM supplemented with 10% FCS per well. The following day, the cells were left unstimulated or were stimulated by overnight incubation with 10^3^ IU/mL IFN-α2a (#11343506, Immunotools), or 10 or 10^3^ IU/mL of IFN-γ (Imukin, Horizon Pharma). Cells were washed and then incubated with infected viral supernatants in the presence of 1 μg/mL protamine. Two days later, the GFP expression of living cells was assessed on a BD FACSVerse flow cytometer.

The SARS-CoV-2 NYC isolate (GenBank OM345241) was obtained from a de-identified patient in July 2020. The virus isolate was amplified though six- to seven-day passages in Caco-2 cells at 37°C. After each passage, virus-containing supernatant was harvested, clarified by centrifugation (3,000 × *g* for 10 minutes), and filtered through a 0.22 μm-mesh disposable vacuum filter system. The passage 3 stock, used in this study, had a titer of 3.4 × 10^6^ PFU/mL, as determined on Vero E6 cells with a 1% methylcellulose overlay, as previously described^177^. SV40-fibroblasts stably transduced with ACE2 were used to seed 96-well plates at a density of 7,000 cells per well in the presence or absence of the indicated doses of IFN-α2b (Introna; MSD) or IFN-γ. The cells were infected with SARS-CoV-2 16 hours later, by adding the 0.1 μL of viral inoculum to the media (final volume 110 μl) and centrifuging the cells for 5 minutes at 500 × *g* at room temperature. The infections were conducted in four replicates (separate wells). The cells were fixed, 24 hours post-infection, with neutral buffered formalin at a final concentration of 10%, stained for SARS-CoV-2 with an anti-N antibody at a dilution of 1:3000 (#GTX135357; GeneTex), then with an Alexa Fluor 647-conjugated secondary antibody (#A-21245; Invitrogen) and 1 μg/mL Hoechst 33342 (# H3570; Invitrogen). Plates were imaged with ImageXpress micro XL and analyzed with MetaXpress (Molecular Devices).

The YF17D-venus reporter virus expressing the Venus fluorescent protein was generated as described by Yi *et al*.^179^; the titer of this virus was 1.8 × 10^6^ TCID_50_/mL on Huh-7.5 cells. SV40-fibroblasts were used to seed 96-well plates at a density of 8,000 cells/well in the presence or absence of 10 or 10^3^ IU/mL IFN-α2b (Introna; MSD), or 10^3^ IU/mL of IFN-γ (Imukin, Horizon Pharma). After incubation for 16 hours, the medium was removed and the cells were inoculated with 50 μL YF17D-venus diluted in Opti-MEM (#51985–091; Gibco). The dilution used was determined empirically (1/32) so as to obtain > 90% YF17D-venus positive IFNAR1-deficient cells at 3 dpi (MOI ≈ 0.3; titer determined on Huh-7.5 cells). After inoculation for 1 hour, the inoculum was removed and 100 μL phenol-free medium (FluoroBrite DMEM; #A1896701; Gibco) supplemented with 10% FBS was added. Infections were performed with six replicates (separate wells). Three hours post-infection, the nuclei of the cells were stained by incubation with 1 μg/mL Hoechst 33342 (#H3570; Invitrogen). Live imaging and quantification were performed with a BioTek Cytation 7 microscope.

For hepatitis A virus assays, SV40-fibroblasts were used to seed 12-well plates. They were incubated at 37°C for 24 h, and were then treated with IFN-α2b (10^3^ IU/mL) or IFN-γ (10^3^ IU/mL), or left untreated for 16 hours before infection with the HM175/18f-NLuc reporter virus (6 ×10^3^ genome equivalents/well)^52^. The cells were incubated for another 72 hours, then harvested and assayed for nanoluciferase (NLuc) activity, as previously described^52^. The replication of influenza A virus (IAV) was assessed as previously described at a MOI of 10, with the A/California/4/2009 strain^181^. The HSV-1 replication experiment was performed as previously described^139,143,183^ with a MOI of 0.001.

The EMCV replication experiment was performed on SV-40 fibroblasts plated at a density of 5×10^4^ cells per well in 48-well plates. The cells were subjected to pretreated with the indicated concentrations of IFN-α2b or IFN-γ, or were left untreated, for 16 hours before infection. Cells were then incubated with EMCV at a MOI of 0.01 for 1 hour, washed twice in PBS, and then transferred to fresh complete DMEM only, or complete DMEM supplemented with the indicated concentrations of IFN-α2b or IFN-γ. Virus samples were collected one hour post-infection (hpi), 12 hpi, 24 hpi, and 48 hpi. Total RNA was extracted from the mixture of cells and supernatant with the Quick-RNA MicroPrep Kit (#R1051, Zymo Research), according to the manufacturer’s protocol. Reverse transcriptase-PCR was performed with random hexamers (#18080–051, Invitrogen). The viral titer was determined with SYBR Green qPCR methods (#4385612, Applied Biosystems) for EMCV *3D*, using the previously described primers^185,187^, with β-glucuronidase (*GUSB*) as the housekeeping gene for normalization. The results are expressed according to the ΔΔCt method, as recommended by the manufacturer.

#### iPSC-derived macrophages (iPSC-MΦ) and MDMs

The iPSC clones C16 and C11 derived from a healthy control, and the iPSC clones derived from a patient with complete STAT1 deficiency complete have been described elsewhere^50,189,191^. We obtained iPSCs from P1 by reprogramming primary fibroblasts with a “4-in-1” 3rd generation SIN lentiviral vector containing the original Yamanaka factors, as previously described^193^. Cells were maintained on irradiated CF1 mouse embryonic fibroblasts (#A34180 or #A34181) cultured in KnockOut DMEM (#10829018) supplemented with 20% KnockOut Serum Replacement medium (#10828028), GlutaMAX Supplement (#35050061), penicillin-streptomycin (#15140122), MEM non-essential amino acids solution (#11140050), and 2-mercaptoethanol (#31350010), all from Thermo Fisher Scientific. Cultures were split weekly with collagenase IV (#17104–019, Gibco). Pluripotency was confirmed by an embryonic stem cell-like morphology, and the expression of SSEA-A, TRA1–60 and alkaline phosphatase. The karyotype was shown to be normal by R-banding and SNP array techniques. We then obtained macrophages from the iPSCs (iPSC-MΦ) as follows: the medium was depleted of bFGF for seven days, and embryonic bodies were allowed to form by incubation on an orbital shaker in the presence of ROCK inhibitor (#1254, Biotechne)^189,191^. After five days, EBs were collected and transferred to differentiation medium composed of STEMdiff APEL2 Medium (#5275, Stem Cell Technologies) supplemented with 50 ng/L recombinant human M-CSF and 25 ng/mL IL-3 (Peprotech)^189,191^. The medium was replaced weekly, and it was possible to harvest monocytic cells from the supernatant after 21 days, for terminal differentiation in RPMI supplemented with 10% FBS, 1% PS and 50 ng/mL M-CSF for 10 days^189,191^.

MDMs were prepared as previously described^195^. Briefly CD14^+^ cells were isolated from PBMCs by positive selection with anti-CD14 MicroBeads (Miltenyi Biotec). Cells were cultured in 12-well plates, in RPMI 1640 containing 10% FBS and M-CSF (50 ng/mL, R&D Systems). On day 7, IL-4 (50 ng/mL, R&D Systems) was added, and the cells were incubated for a further seven days for the completion of MDM differentiation. Primary fibroblasts, iPSC-MΦ, or MDMs were left unstimulated or were stimulated with 10^3^ IU/mL recombinant IFN-γ (Imukin, Horizon Pharma).

#### RNA-seq

Primary fibroblasts were plated at a density of 700,000 cells per well in six-well plates, in 2 mL DMEM supplemented with 10% FBS. They were starved overnight in DMEM with 0.3% BSA (#A1595, Sigma-Aldrich). Terminally differentiated iPSC-MФ were positively selected with anti-CD14 MicroBeads (Miltenyi Biotec) to ensure that a pure population was obtained and >50,000 cells per well were plated in 12-well plates, in RPMI supplemented with 10% FBS and 100 ng/mL M-CSF. Primary fibroblasts, iPSC-MФ, and MDMs were left unstimulated or were stimulated for 2 or 8 hours with 10^3^ IU/mL recombinant IFN-γ (Imukin, Horizon Pharma). Primary fibroblasts were left unstimulated or were stimulated with 10^3^ IU/mL recombinant IFN-α2b (Introna, MSD). Total RNA was extracted from cells with the Quick-RNA MicroPrep kit (#R1051, Zymo Research), and treated with DNAse (Zymo) to remove residual genomic DNA. Ribosomal RNA (rRNA) and mitochondrial RNA (mRNA) were depleted. RNA-seq libraries were prepared with the Illumina RiboZero TruSeq Stranded Total RNA Library Prep Kit (Illumina) and sequenced on a NovaSeq machine in the 100- or 150-nt, paired-end configuration. The RNA-seq FASTQ files were first inspected with FastQC (https://www.bioinformatics.babraham.ac.uk/projects/fastqc/) to ensure that the raw data were of high quality. For each subject, the two FASTQ files generated were then mapped onto the human reference genome (Ensembl GRCh37 release 75) with STAR v.2.7.3a, in the two-pass mode^197^. The mapping quality of each BAM file was then evaluated with RSeQC^198^. Reads were quantified to generate gene-level feature counts from the read mapping, with HTSeq-count v.0.11.2^199^. We normalized the datasets with the functions DGEList and calcNormFactors from the edgeR version 3.26.8 package^200^ implemented in R v.3.6.3. We retained only genes with at least 1 count-per-million (CPM) in at least two samples. We considered a gene to be differentially expressed between two sets of conditions if the log_2_-fold-change between the two sets of conditions was greater than 1 (absolute value) and the adjusted *p*-value was below 0.05, according to the calculations made with the DESeq function of the DESeq2 package version 1.24.0^201^ implemented in R. Differential gene expression data were plotted on heatmaps with heatmap.2 implemented in R. We used the findMotifs.pl script from HOMER v4.11^202^ with the human v.6.3 database and the parameter -len 12.

#### Generation of THP1 cells with knockouts of IFN-γR1 or STAT1

THP1^KO^ cells were prepared as previously described^8^. Briefly, the plasmid was digested with *Bsmb*I. Annealed forward and reverse sgRNA for the target sequence (*IFNGR1* exon 3 or *STAT1* exon 3) was inserted into the pLentiCRISPRv2 plasmid digested with *Bsmb*I. The resulting plasmid was used to transduce HEK293T cells in the presence of pCMV-VSV-G (#8454, Addgene), pHXB2 (#1069, NIH-AIDS Reagent Program), psPAX2 (#12260, Addgene), and pLentiCRISPRv2 (#52961, Addgene) with inserted sgRNA. After 24 hours, the supernatant of HEK293T cells was filtered through a membrane with 45 μm pores, protamine was added to a final concentration of 8 μg/mL, and 100 μL of the viral supernatant was added to 200,000 THP1 WT cells in a 96-well round-bottomed plate. Spinoculation was performed for 2 hours at 1200 × *g*. Two days later, selection on 5 ng/mL puromycin was initiated for five days. The cells were then cultured under single-clone conditions. After expansion IFN-γR1^KO^ or STAT1^KO^ clones completely deficient for the molecules concerned were identified on the basis of their (i) on complete lack of expression of IFN-γR1 or STAT1, respectively, on flow cytometry; (ii) complete lack of induction of HLA-DR after 24 hours of stimulation with 10^4^ IU/mL IFN-γ (Imukin, Horizon Pharma).

#### Infection of macrophages with bacteria and mycobacteria

THP1 WT cells, the previously described IRF1^KO^ cells^78^, or custom-generated IFN-γR1^KO^ or STAT-1^KO^ cells were cultured in FCS-supplemented RPMI. Theydifferentiated into adherent macrophages with 48 hours of PMA treatment (20 ng/mL, #P8139–1MG, Sigma Aldrich). The cells were then left unstimulated or were stimulated for 24 hours with IFN-γ at a concentration of 10^3^ IU/mL (Imukin, Horizon Pharma). Cells were then infected as previously described^74^ with *Salmonella enterica* subsp. *enterica* serovar Typhimurium GFP (14028GFP, ATCC), in a gentamicin protection assay. Briefly, *Salmonella* Typhimurium-GFP was cultured overnight in LB broth supplemented with 100 μg/mL ampicillin. The overnight culture was then diluted 1:33 in the same medium and cultured for another three hours until it reached exponential growth phase. The bacteria were then washed once with 1X PBS and used to infect cells at a MOI of 5–10. After 45 minutes, the medium was changed and the cells were incubated with gentamicin (#15750060, Thermo Fisher Scientific) at a concentration of 100 μg/mL for 1 hour. The medium was then removed; the cells were gently washed three times with 1X PBS and incubated with RPMI supplemented with 10% FCS and 20 μg/mL gentamicin with or without 10^3^ IU/mL IFN-γ for the indicated infection time. The cells were then treated with trypsin, and fixed by incubation with 4% paraformaldehyde (#30525-89-4, Santa Cruz) for 20 minutes at room temperature. Cells were acquired on a Beckman Coulter Gallios flow cytometer and analyzed with FlowJo Software. The infection of MDMs (differentiation with M-CSF and IL-4 (#216-MC-010 and #204-IL-010, respectively, R&D Systems)) with *M. abscessus*-tdTomato^203^ and intracellular CFU determination were performed as previously described^204^. Infected MDMs were acquired on a Beckton Dickinson Fortessa flow cytometer and analyzed with FlowJo Software.

#### CITE-seq

Single-cell RNA-seq profiling was performed on a first PBMC sample from P1 (P1.1 sampled at the age of three years), and cellular indexing of transcriptomes and epitopes by sequencing (CITE-seq) was performed on PBMCs obtained from P1 (P1.2, sample obtained at the age of five years), P2 (sample obtained at the age of six years), one pediatric control and two other healthy controls. Both patients were receiving broad-spectrum antimycobacterial drugs. P1 was also receiving recombinant IFN-γ at the age of five years but not at the age of three years. The frozen PBMCs were quickly thawed at 37°C and gently resuspended by serial additions of DMEM + 10% heat-inactivated FBS, to obtain a final volume of 14 mL. The cell suspension was centrifuged at 300 × *g* for 5 minutes, and the cells were washed twice with 5 mL DMEM + 10% HI-FBS each to remove cell debris. Cells were counted and viability was assessed with the LIVE/DEAD Viability kit (Thermo Fisher Scientific), according to the manufacturer’s guidelines. Residual red blood cells were removed with 25 μL of the antibody cocktail and beads from the StemCell EasySep PBMC isolation kit. One million cells were resuspended in 1 mL Biolegend Cell Staining Buffer and passed through a 40 μm-mesh Flowmi cell strainer (Sigma) to remove aggregates. Cells were then centrifuged at 400 × *g* for 5 minutes and resuspended in 45 μL Biolegend Cell Staining Buffer supplemented with 5 μL Human TruStain FcX Fc block (Biolegend). Cells were then incubated for 30 minutes at 4°C with a cocktail of DNA-barcoded TotalSeq-B antibodies (Biolegend): CD11c (S-HCL-3), CD141 (M80), CD161 (HP-3G10), CD14 (Me5E2), CD16 (3G8), CD19 (HIB19), CD1c (L161), CD28 (CD28.2), CD370 (CLEC9A) (8F9), CD38 (HB-7), CD3e (UCHT1), CD4 (RPA-T4), CD45RA (HI100), CD45RO (UCHL1), CD56 (NCAM) (5.1H11), CD66b (6/40c), CD69 (FN50), CD8a (RPA-T8), TCR gd (B1), TCR Va7.2 (3C10), IgG1 k Isotype control (MOPC-21). Cells were washed twice in cell staining buffer, once in PBS + 0.04% BSA, and resuspended at a concentration of 1000 cells/μL in PBS + 0.04% BSA. The samples were loaded onto a 10X Genomics Chromium G chip and reverse transcription and library preparation were performed with the Chromium Single-Cell 3′ Reagent Kits (v2 for P1.1, and v3.1 for the others), in accordance with the manufacturer’s instructions. The quality of the cDNA and feature barcode library was assessed with a TapeStation (Agilent), before sequencing on the S4 flow cells of an Illumina NovaSeq 6000 sequencer.

Two additional control PBMC scRNA-seq datasets were obtained from another study that we performed^205^. For the analysis, we included scRNA-seq for P1.1 and 2 controls, and CITE-seq for 3 controls and the P1.2 and P2 samples. The analysis was performed as previously described^205^. Briefly, sequence read quality was assessed with BVAtools (https://bitbucket.org/mugqic/bvatools). Cell Ranger (v3.0.1 for scRNA and v6.0.1 for CITE-seq) was used to map reads to the hg38 human reference genome assembly, to perform filtering, and to count barcodes and unique molecular indices (UMIs). Genes that were not expressed in any of the datasets were discarded. Cells with >20% mitochondrial genes were excluded. We filtered out low-quality cells and doublets, by excluding cells falling outside the [−1SD;+2.5SD] interval from the UMI and gene count distributions. We identified and removed any remaining doublets by manually removing cells that co-expressed markers for different cell types (CD14, CD79A, TRBC1, HBA2 and LILRA4). We then used DoubletFinder package v2.0.3 to identify and filter out any remaining cell doublets^206^. Once dead cells and doublets had been removed, the patient and control samples were analyzed together with the Seurat v4.0.2 R package^207^ and cell clustering was performed by the Uniform Manifold Approximation and Projection dimension reduction method^208^ applied to the most variable genes, but excluding mitochondrial and ribosomal protein genes, together with sex-related genes as both patients were female. Based on the clustering pattern obtained, we performed subclustering for the large immune cell lineages separately (myeloid/pDC, T/NK, and B cells) to identify any remaining doublets. Small residual cell clusters expressing multiple cell type-specific markers were, therefore, excluded. A final UMAP^208^ clustering identified 14 different cells, including 11 T cells and 2 B cells; myeloid cells were excluded from further analyses because of the small numbers of these cells in the patient samples, probably due to the poor survival of these cells during sample freezing and shipping. The protein markers identified with CITE-seq were used to assign a cell-type identity to each cluster, in conjunction with the MAST approach^209^ to identify the marker genes for each cluster. Differential gene expression analysis was performed with MAST for each lymphoid cell cluster, by comparing the RNA levels in P1.2 and P2 with those in five healthy controls. Adjusted *p*-values were estimated with the Benjamini-Hochberg (BH) procedure. Every significant differentially expressed gene (absolute FC >=1.5 and adj-p-value ≤0.001) was classified as down- or upregulated, extracted and used for downstream analysis. All the differential expression results are provided in Table S6. Enrichment analyses for GO biological processes, KEGG and Reactome pathways and Transfac motifs were performed separately on up- and downregulated differentially expressed genes and for each cell subpopulation, with the gProfiler R package and the g:SCS threshold method^210^; all the enrichment results are provided in Table S6.

We evaluated the effects on IRF1-binding genes in the cells of the patients by estimating module scores with Seurat’s AddModuleScore function, using the default settings. Module score was determined for each cell by calculating the mean transcriptional activity of the genes from the module and then subtracting a mean expression value for a set of control genes. The list of IRF1-binding genes was obtained as follows: IRF1 binding sites were identified by ChIP-seq in IFN-γ-treated (3 h) mouse bone marrow-derived macrophages obtained in a previous study^58^, we then retain genes with IRF1 sites within 10 kb on either side of the transcription start site and performed a conversion to human orthologs. Control genes were randomly sampled from bins defined based on the observed level of expression for the genes in the module. The significance of differences between expression in the patients and control module expression was then assessed by comparing the corresponding module score distributions for each cell subset in a Wilcoxon signed rank-test approach (q-value <= 0.05). We obtained a sample size-free evaluation of the difference in expression by calculating Cohen’s *d* effect size for every significant variant.

The scRNA-seq and CITE-seq datasets have been deposited in NCBI’s Gene Expression Omnibus and are accessible through GEO Series accession number GSE216489.

#### Generation of single-cell suspensions from murine blood and tissues for flow cytometry

All mice were kept in specific pathogen-free conditions and handled according to the guidelines and regulations of the Canadian Council on Animal Care. Experimental protocols were approved by the McGill University Institutional Animal Care Committee (protocol number 2018–8014). Groups of male C57BL/6 (B6) and *Irf1*^−/−^ mice (originally from The Jackson Laboratory, reared in-house) were killed, and their blood was collected by cardiac puncture and transferred to heparin-containing tubes (Sarstedt). Blood was diluted 1:5 in PBS, centrifuged in a benchtop centrifuge and the red blood cells in the pellet were lysed with Red Blood Cell Lysing Buffer Hybri-Max (Sigma-Aldrich) according to the manufacturer’s instructions. Cells were washed twice with FACS buffer (PBS supplemented with 2% heat-inactivated FBS) and resuspended in FACS buffer. Mouse lungs were processed as previously described^211^ but with minor modifications. Briefly, lungs were perfused with 5 mL ice-cold PBS, isolated and finely minced. Lung pieces were digested by incubation in 2.5 mL lung digestion buffer (RPMI-1640 supplemented with 5% FBS, 0.2 mg/mL Liberase [Roche] and 0.1 mg/mL DNase I [Roche]) for 50 minutes at 37°C. Digests were homogenized by passage through a syringe (18 G needle) and a cell strainer with 70 μM pores. Cells were washed with PBS and red blood cells were lysed with Red Blood Cell Lysing Buffer Hybri-Max (Sigma-Aldrich). Cells were washed twice and resuspended in FACS buffer. For the generation of single-cell splenocyte suspensions, spleens were isolated, gently homogenized between the frosted ends of glass slides and digested by incubation in 3 mL spleen digestion buffer (HBSS supplemented with 10 mM HEPES, 150 mM NaCl, 5 mM KCl, 1 mM MgCl_2_, 1.8 mM CaCl_2_, 1 mg/mL collagenase D [Roche] and 0.2 mg/mL DNase I [Roche]) for 30 minutes at 37°C. Cell suspensions were further homogenized by pipetting and incubated for an additional 15 minutes at 37°C. EDTA was added to a final concentration of 1 mM and the suspensions were incubated for another 10 minutes at room temperature. Splenocytes were collected by centrifugation. The pellet was washed with PBS and red blood cells were lysed with Red Blood Cell Lysing Buffer Hybri-Max (Sigma-Aldrich). Cells were washed twice and resuspended in FACS buffer. Bone marrow was isolated from femurs and tibiae and red blood cells were lysed with Red Blood Cell Lysing Buffer Hybri-Max (Sigma-Aldrich). Cells were washed twice and resuspended in FACS buffer.

#### Flow cytometry on mouse cells

Pelleted cells were resuspended in 2.4G2 hybridoma supernatant diluted in FACS buffer and the resulting suspensions were incubated for 15 minutes on ice to block Fcγ receptors. Surface markers were then stained by incubation with the corresponding fluorochrome-labeled antibody dilutions in FACS buffer for 30 minutes on ice. Dead cells were excluded by staining with Fixable Viability Dye eFluor 780 (Thermo Fisher Scientific) according to the manufacturer’s instructions. Intracellular staining was performed with the FoxP3/Transcription factor staining kit (Thermo Fisher Scientific), in accordance with the manufacturer’s protocol. Stained cell suspensions were acquired on a BD LSRFortessa Cell Analyzer (BD Biosciences) and data were analyzed with FlowJo v.10 software (BD Biosciences). All the antibodies used for murine flow cytometry analyses are listed in STAR Methods.

### QUANTIFICATION AND STATISTICAL ANALYSIS

Unless otherwise indicated in the figure legends, the statistics provided correspond to independent experiments. Mann-Whitney or Student’s *t*-tests were performed. In the relevant figures, n.s. indicates not significant, *p*>0.05; **** *p*<0.0001; ***p < 0.001; **p < 0.01; and *p < 0.05. Analyses were performed with GraphPad software.

## Supplementary Material

Figure S1 **Figure S1 – Identification of homozygous complete loss-of-function *IRF1* variants in patients with severe MSMD, Related to** Figure 1. **(A)** Cells involved in the production of and response to IFN-γ. Proteins for which a mutation of the corresponding gene has been recognized to cause isolated MSMD are depicted in blue, those responsible for syndromic MSMD are shown in red, and those that can cause either isolated MSMD or syndromic MSMD are depicted with crossed lines. **(B)** Relative clinical severity of isolated MSMD forms vs. relative residual IFN-γ activity. **(C)** Principal component analysis of WES data from the two patients and our in-house WES database. **(D)** Consensus negative selection (CoNeS) of *IRF1*. **(E)** Diagram of the various *IRF1* cDNA constructs used for transient overexpression experiments. **(F)** RT-qPCR on HEK293T cells without (NT) and with transfection with empty vector (EV) or various *IRF1* cDNAs. The bars represent the mean. Data from two to four independent experiments are shown. **(G)** Western blot for DDK, IRF1, lamin A/C, and vinculin on cytoplasmic (C) and nuclear (N) extracts from HEK293T cells not transfected (NT) or transfected with EV or various *IRF1* cDNAs. Data representative of three independent experiments are shown. **(H)** EMSA and supershift of the nuclear extract of HEK293T cells transfected with EV or various *IRF1* cDNAs with DDK tagging of the C-terminus of the protein, incubated with a fluorescent ISRE probe. Representative data from two independent experiments are shown**. (I)** Dual luciferase ISRE5 reporter activity of HEK293T cells transfected with EV or various *IRF1* cDNAs. Bars represent the mean and SD. Data from three to six independent experiments are shown. Statistical analysis was performed with Student’s *t*-test. ns = not significant, *p* > 0.05; ****p < 0.0001.

Figure S2 **Figure S2 – IRF1 protein levels in cells from IRF1-deficient patients, Related to** Figure 2. **(A)** Western blot for IRF1, IRF3, IRF9, STAT1, and vinculin on total lysate from HVS-T cells from three healthy controls (CTLs), P1, and a patient with complete IRF9 deficiency. Data from a single experiment are shown. **(B)** IRF1 expression in primary fibroblasts from three CTLs, P1, P2, and a patient with complete IFN-γR1 deficiency, with and without 16 hours of stimulation with 10^3^ IU/mL IFN-γ. Intracellular IRF1 (upper panel) and STAT1 (lower panel) levels, assessed by flow cytometry on **(C)** HVS-T cells (from a CTL and P1), **(D)** SV40-fibroblasts (from a CTL, P1, P2, and from a patient with complete STAT1 deficiency), or **(E)** EBV-B cells (from a CTL, P1, P2, and from a patient with complete STAT1 deficiency). **(F)** IRF1 and STAT1 expression in SV40-fibroblasts from three CTLs, P1, and a patient with complete IFN-γR1 deficiency, with and without 16 hours of stimulation with 10^3^ IU/mL IFN-γ, or in HEK293T cells transfected with p.R129-DDK *IRF1* cDNA. Data representative of three independent experiments are shown.

Figure S3 **Figure S3 – Leukocyte development in IRF1-deficient patients and in IRF1-deficient mice, Related to** Figure 3. **(A)** Monitoring of counts of leukocytes, monocytes and lymphocytes, polymorphonuclear neutrophils (PMN), polymorphonuclear eosinophils (PME), and polymorphonuclear basophils (PMB) in the blood of the patients, in red. **(B)** Mass or flow cytometry analysis of cryopreserved PBMC subsets from CTLs, P1, and P2. **(C)** Flow cytometry analysis of dendritic cell (DC) subsets in fresh PBMCs from a CTL, P1, and P2. **(D)** Flow cytometry immunophenotyping of live CD45^+^ lymphocytes from PBMCs, for ILCP (CD117^+^CRTh2^−^) and ILC2 (CRTh2^+^), in CTLs, P1, and P2. **(E)** Conventional flow cytometry counts of cDC1, cDC2, NK cells, ILC2, CD4^+^ T cells, Treg and CD8^+^ T cells, in the lungs of WT mice (*n*=5), and IRF1-deficient mice (*n*=5). **(F)** Conventional flow cytometry counts of Ly6C^+^ monocytes, cDC1, cDC2, pDCs, NK cells, CD4^+^ T cells, Treg and CD8^+^ T cells in the spleen of WT mice (*n*=5), and IRF1-deficient mice (*n*=5). **(G)** Conventional flow cytometry counts of CLP, CHILP, and ILC2P in the bone marrow of WT mice (*n*=5), and IRF1-deficient mice (*n*=5). Unpaired two-tailed *t*-tests were used for the statistical analysis. *p < 0.05; ***p < 0.001.

Figure S4 **Figure S4 – IFN-γ production pathway in P1 and P2, Related to** Figure 4. **(A)** Western blot analysis of ISG15 and MX1 induction in the SV40-fibroblasts of three CTLs, P1, P2, one patient with complete ISG15-deficiency, and one patient with complete IFN-αR1 deficiency. **(B)** pSTAT4 levels in HVS-T cells from two controls (CTLs), P1, and a patient with complete IL-12Rβ1 deficiency without stimulation (gray), and after 15 minutes of stimulation with 50 ng/mL IL-12 (solid black line), or 10^5^ IU/mL IFN-α2b (dotted black line). Data representative of two independent experiments are shown. **(C)** Production of IFN-γ by the HVS-T cells of controls (CTLs), P1, and a patient with complete IFN-γ deficiency with and without 2 hours of stimulation with brefeldin, anti-CD3/CD2/CD28 mAb-coated beads, or with PMA-ionomycin. Bars represent the mean and SD. Data from one experiment are shown. **(D)** Analysis of IFN-γ^+^ cells on intracellular flow cytometry for various PBMC subsets, following 48 hours of stimulation with IL-12, IL-23, or BCG. Bars represent the mean and SD. Technical duplicates of the same experiment are shown for P1 and P2. **(E)** Intracellular flow cytometry analysis of IFN-γ, TNF, IL-9, IL-13, IL-4, IL-17A, IL-17F, IL-2, and IL-21 levels in naïve CD4^+^ T cells from P1 and controls after expansion for 5 days with anti-CD2/CD3/CD28 mAb-coated beads and IL-2, followed by culture for 7 days in polarizing conditions, and stimulation with PMA-ionomycin in the presence of brefeldin for 6 hours.

Figure S5 **Figure S5 – Response to IFN-γ in non-hematopoietic cells from P1 and P2, Related to** Figure 5. *IRF1* RT-qPCR **(A)** and western blot for IRF1, pSTAT1, STAT1 and vinculin **(B)** on SV40-fibroblasts with and without stimulation with 10^3^ IU/mL IFN-γ. Data from one experiment are shown. **(C)** Confocal microscopy of IRF1 in SV40-fibroblasts of a CTL, P1, and a STAT1-deficient patient stimulated for the indicated times. Phosphorylation of STAT1, analyzed by staining and flow cytometry after 20 minutes of incubation with or without 10^5^ IU/mL IFN-γ, in EBV-B cells **(D)**, or with or without 10^3^ IU/mL IFN-γ in SV40-fibroblasts **(E)** from controls (CTLs), the patients (P1 and P2), or patients with complete IFN-γR1 or STAT1 deficiency. Data representative of two independent experiments are shown. Heatmaps of genes differentially expressed (log_2_FC) in primary fibroblasts from controls or patients, after **(F)** 2 or **(G)** 8 hours of stimulation with 10^3^ IU/mL IFN-γ. We show the genes differentially expressed in patients relative to the control group at both time points (2 or 8 hours), for the genes differentially expressed at both time points relative to non-stimulated fibroblasts in the control group, *i.e*., with a |log_2_(FC)| > 1 and adj. *p*-value < 0.05 in controls, and with a |log_2_(FC)| > 1 and adj. *p*-value < 0.05 in patients relative to controls at the corresponding time point. We used the IFN-γR1^−/−^ patient as a negative control. **(H)** Amount of mRNA, assessed by RNA sequencing for IFN-α2b-inducible genes (logFC>2 in controls) in primary fibroblasts from the indicated individuals with and without 30 minutes, 2 hours, and 8 hours of stimulation with IFN-γ. **(I)** and **(J)** Flow cytometry analysis of CD274 (PD-L1) expression with and without stimulation for 48 hours with 10^2^, 10^3^, or 10^4^ IU/mL IFN-γ in SV40-fibroblasts with and without stable retrotransduction with EV or WT *IRF1* cDNA. **(K)** Flow cytometry analysis of the CD274 expression of primary fibroblasts with and without 48 hours of stimulation with 10^2^ or 10^3^ IU/mL IFN-γ.

Figure S6 **Figure S6 – Response to IFN-γ in myeloid cells from P1 and P2, Related to** Figure 6. Induction of IL-12p40 **(A)** and IL-12p70 **(B)** secretion in whole-blood assays for local and travel controls (CTLs), an IL-12p40-deficient patient, P1, and P2, following stimulation with live BCG alone or in combination with IFN-γ (5,000 IU/mL). Bars represent the mean. Heatmaps of genes differentially expressed (log_2_(FC)) in iPSC-derived macrophages (iPSC-MΦ), and monocytes-derived macrophages (MDMs) from controls (CTL) or patients, after **(C)** 2 or **(D)** 8 hours of stimulation with 10^3^ IU/mL IFN-γ. We show the genes differentially expressed in patients relative to the control group at both time points (2 or 8 hours), for genes differentially expressed at both time points relative to non-stimulated cells in the control group, *i.e*., with a |log_2_(FC)| > 1 and adj. *p*-value < 0.05 after Benjamini-Hochberg correction in controls, and with a |log_2_(FC)| > 1 and adj. *p*-value < 0.05 after correction in patients relative to controls at the corresponding time point. **(E)** Fold-enrichment in dysregulated genes, as determined by RNA-seq, for the myeloid cells of both patients relative to IRF1 ChIP-seq results for resting human monocytes^77^ and IFN-γ-activated murine bone marrow-derived macrophages^58^. **(F)** RT-qPCR for *APOL3* and *GBP4* in iPSC-MΦ or MDMs with and without stimulation for 2 and 8 hours with 10^3^ IU/mL IFN-γ. The bars represent the mean. **(G)** Examples of flow cytometry dot plots obtained for gentamicin protection assay performed on PMA-differentiated WT or IRF1^KO^, IFN-γR1^KO^, or STAT1^KO^ THP1-derived macrophages (THP1-Ф) with and without 24 hours of pretreatment with 10^3^ IU/mL IFN-γ and infection with *Salmonella* Typhimurium-GFP (Stm-GFP) at a MOI of 6 for 8 hours. The percent *Salmonella* Typhimurium^+^ cells is displayed in black and the percent *Salmonella* Typhimurium^high^ cells is displayed in red. **(H)** Examples of flow cytometry dot plots obtained for Figure 6H.

Figure S7 **Figure S7 – Antiviral IFN-α/β and IFN-γ cell-intrinsic immunity of IRF1-deficient fibroblasts, Related to** Figure 7. **(A)** RT-qPCR for *IRF1* and *MX1* in SV40-fibroblasts from the indicated controls or patients after 2 hours of stimulation with 10^3^ IU/mL IFN-α2a or IFN-γ. **(B)** *IRF1* levels determined by RNA sequencing, in primary fibroblasts stimulated for 30 minutes, 2 hours, or 8 hours with IFN-α2b or IFN-γ. Results are normalized against non-stimulated conditions. **(C)** Western blot of IRF1, MX1, STAT1, STAT2, IRF9, and vinculin in SV40-fibroblasts from controls (CTLs), the patients (P1, P2), or patients with complete STAT1, STAT2, IRF9, IFN-αR1, or IFN-γR1 deficiencies stimulated for 4 hours with IFN-γ or IFN-α2β. **(D)** Phosphorylation of STAT1, as assessed by staining and flow cytometry, after 20 minutes of incubation with or without 10^3^ IU/mL IFN-α2b or 10 ng/mL IFN-β, in SV40-fibroblasts or EBV-B cells from two controls (CTLs), the patients (P1, P2), and patients with complete IFN-αR1 or STAT1 deficiency. Data representative of two to three experiments are shown. **(E)** Venn diagram of genes positively induced in primary fibroblasts of CTLs (log2FC>1) but less strongly induced (log2FC<−1) in IRF1-, STAT1-, or IRF9-deficient primary fibroblasts than in controls after 2 or 8 hours of stimulation with IFN-α2b. **(F)** Hepatitis A virus (HAV), **(G)** human immunodeficiency virus-1 (HIV-1), and **(H)** yellow fever vaccine virus-venus (YF17D-venus) replication in SV40-fibroblasts for the indicated controls or patients (P1 and P2), after pretreatment for 16 hours with the indicated doses of IFN-α2b or IFN-γ. All viral infections were performed in two or three independent experiments.

Supplementary References

Supplementary Tables

Tables S6-S7 **Table S6 –CITE-seq data, Related to** Figure 3. Differential gene expression analysis for each scRNA-seq lymphoid cell cluster and enrichment analysis for genes significantly up- and downregulated in scRNA-seq analyses of patient cells. Log_2_FC and adjusted *p* values for the comparison of gene expression of P1.2 and P2 cells with healthy controls cells; an absolute FC ≥1.5 and adj. *p* value ≤0.001 were considered significant. Gene ontology (biological process), pathway and Transfac motif-enriched genes are analyzed.
**Table S7 –RNA sequencing and mass-spectrometry data, Related to** Figures 5, 6, and 7. – **(Sheet 1)** log_2_FC and adj. *p*-value for RNA-seq on primary fibroblasts from three controls, both IRF1-deficient patients (P1 and P2), and patients with autosomal recessive deficiencies of IRF9, STAT1, IFN-αR1, IFN-γR1, or IFN-γR2, with and without stimulation for 30 minutes, 2 hours, or 8 hours with 10^3^ IU/mL IFN-γ or IFN-α2b. **(Sheet 2)** – log_2_FC and adj. *p*-value for RNA-seq on iPSC-MΦ from two clones from controls (#11 and #16), P1 (#10), and a patient with autosomal recessive STAT1 deficiency, with and without stimulation for 2 or 8 hours with 10^3^ IU/mL IFN-γ. **(Sheet 3) –** log_2_FC and adj. *p*-value for RNA-seq on MDMs from three controls and P2, with and without stimulation for 2 or 8 hours with 10^3^ IU/mL IFN-γ. **(Sheet 4)** – Mass spectrometry quantification of protein extracted from primary fibroblasts from the indicated individuals with and without prior stimulation for 24 hours. Values were log_2_-transformed and normalized against the median.

## Figures and Tables

**Table:** KEY RESOURCES TABLE

REAGENT or RESOURCE	SOURCE	IDENTIFIER
**Antibodies**		
Human IRF1 (clone D5E4)	Cell Signaling	Cat# 8478, RRID:AB_10949108
Human IRF1 (rabbit polyclonal)	Proteintech	Cat# 11335-1-AP, RRID:AB_2877759
Human IRF8 (goat polyclonal)	Santa Cruz	Cat# sc-6058, RRID:AB_649510
Human IRF8 (clone D20D8)	Cell signaling	Cat# 5628, RRID:AB_10828231
Human IRF9 (rabbit polyclonal)	Santa Cruz	Cat# sc-496, RRID:AB_2127709
Human IRF9 (rabbit polyclonal)	Proteintech	Cat# 14167-1-AP, RRID:AB_2296227
Human IRF3 (clone D9J5Q)	Cell Signaling	Cat# 10949, RRID:AB_2797733
Human STAT1 (clone 1)	Beckton-Dickinson	Cat# 610115, RRID:AB_397521
Human pSTAT1 (clone 4a)	Beckton-Dickinson	Cat# 612232, RRID:AB_399555
Human STAT2 (clone B-3)	Santa Cruz	Cat# sc-514193, RRID:AB_2810271
Human MX1 (polyclonal)	ProteinTech	Cat# 13750-1-AP, RRID:AB_2266768
Human ISG15 (clone F-9)	Santa-Cruz	Cat# sc-166755, RRID:AB_2126308
Human vinculin (clone EPR8185)	Abcam	Cat# ab129002, RRID:AB_11144129
Human vinculin (clone 7F9)	Santa Cruz	Cat# sc-376248-HRP, RRID:AB_10991536
Anti-mouse IgG (H + L)-HRP-conjugated	Bio-Rad	Cat# 170-6516, RRID:AB_11125547
Anti-rabbit IgG (H + L)-HRP-conjugated	Bio-Rad	Cat# 170-6515, RRID:AB_11125142
Human DDK-tag (clone M2)	Sigma-Aldrich	Cat# A8592, RRID:AB_439702
Human DDK (clone M2)	Cell Signaling	Cat# 14793, RRID:AB_2572291
Human Isotype rabbit	Cell Signaling	Cat# 2729, RRID:AB_1031062)
Human GBP1 (clone 1B1)	Santa-Cruz	Cat# sc-53857, RRID:AB_2109333
Human APOL3 (clone EPR8238)	Abcam	Cat# ab154869
Human RARRES3 (rabbit polyclonal)	ProteinTech	Cat# 12065-1-AP, RRID:AB_2175704
anti-rabbit IgG Alexa Fluor 555	ThermoFischer Scientific	Cat# A-21429, RRID:AB_2535850
PE-Dazzle-594- Human PD-L1 (CD274) antibody (clone 29E.2A3)	BioLegend	Cat# 329732, RRID:AB_2616889
PE-Dazzle-594-Mouse IgG2b, κ Isotype Ctrl Antibody	BioLegend	Cat# 400358
PE-Human STAT1 (clone 1)	Beckton-Dickinson	Cat# 558537, RRID:AB_647231
PE-Human pSTAT1 (clone 4a)	Beckton-Dickinson	Cat# 612564, RRID:AB_399855
AF647-Human pSTAT1 (clone 4a)	Beckton-Dickinson	Cat# 612597, RRID:AB_399880)
PE Mouse IgG1, κ Isotype Control	Beckton-Dickinson	Cat# 554680, RRID:AB_395506
AF647 Mouse IgG1, κ Isotype Control	Beckton-Dickinson	Cat# 565363, RRID:AB_2869665
PE-conjugated goat anti-rabbit	Thermo Fisher Scientific	Cat# A10542, RRID:AB_2534042
Human IFN-γ-BUV737 clone 4S.B3	BD Horizon	Cat# 564620, RRID:AB_2869591
Human TNF-PerCP clone Mab11	BioLegend	Cat# 502924, RRID:AB_2561288
Human IL-9-PE clone MH9A3	BD Pharmingen	Cat# 560807, RRID:AB_2033985
Human IL-13-BV421 clone JES10-5A2	BD Horizon	Cat# 563580, RRID:AB_2738290
Human IL-4-AF488 clone 8D4-8	BioLegend	Cat# 500710, RRID:AB_1877131
Human IL-17A-BV510 clone BL168	BioLegend	Cat# 512330, RRID:AB_2562745
Human IL-17F-BV650 clone O33-782	BD Horizon	Cat# 564264, RRID:AB_2869555
Human IL-2-BV750 clone MQ1-17H12	BD Horizon	Cat# 566361, RRID:AB_2739710
Human IL-21-eF660 clone eBio3A3-N2	Thermo Fisher Scientific	Cat# 50-7219-42, RRID:AB_10598202
Human aCD3-Alexa532 (Clone UCHT1)	Thermo Fisher Scientific	Cat# 58-0038-42, RRID:AB_11218675
Human γδTCR-FITC (clone)	Thermo Fisher Scientific	Cat# 11-9959-42, RRID:AB_10669049
Human Vδ2-APC-Fire750)	BioLegend	Cat# 331420, RRID:AB_2687326
Human CD56-BV605 (clone 5.1H11)	BioLegend	Cat# 362538, RRID:AB_2565856
Human CD4-BV750	BD Biosciences	Cat# 566356, RRID:AB_2744426
Human CD8a-BV510 (clone RPA-T8)	BioLegend	Cat# 301047, RRID:AB_2561378
Human Vα7.2-BV711 (clone 3C10)	BioLegend	Cat# 351731, RRID:AB_2629679
Human Vα24-Jα18-PE-Cy7 (clone 6B11)	BioLegend	Cat# 342912, RRID:AB_2562230
Human Vδ1-Vioblue	Miltenyi Biotec	Cat# 30-100-555
Human CD161-PE (clone HP-3G10)	BioLegend	Cat# 339938, RRID:AB_2564141
Human Vβ11-APC (Miltenyi Biotec)	Miltenyi Biotec	Cat# 58-0038-42, RRID:AB_11218675
Human CD1a-biotin (clone HI149)	BioLegend	Cat# 300112, RRID:AB_389344
Human CD14-biotin (clone 61D3)	Invitrogen	Cat# 13-0149-82, RRID:AB_466373
Human CD34-biotin (clone 4H11)	BioLegend	Cat# 316404
Human CD123-biotin (clone 6H6)	BioLegend	Cat# 306004, RRID:AB_314578
Human CD203c-biotin (clone FR316A11)	Miltenyi Biotec	Cat# 130-092-345, RRID:AB_615067
Human CD303-biotin (clone AC144)	Miltenyi Biotec	Cat# 130-090-691, RRID:AB_244166
Human FcεRIα-biotin (clone AER-37 CRA-1)	BioLegend	Cat# 334606, RRID:AB_2571885
Human TCRαβ-biotin (clone IP26)	BioLegend	Cat# 306704, RRID:AB_314632
Human TCRγδ-biotin (clone B1)	BD Biosciences	Cat# 555716, RRID:AB_396060
Human CD4 FITC (clone OKT4)	BioLegend	Cat# 317408, RRID:AB_571951
Human CD336 PerCP-eFluor710 (clone 44.189)	Thermo Fisher Scientific	Cat# 46-3369-42, RRID:AB_2573749
Human EOMES PE (clone WD1928)	Thermo Fisher Scientific	Cat# 12-4877-42, RRID:AB_2572615
Human CD8a PE-CF594 (clone RPA-T8)	BD Biosciences	Cat# 562282, RRID:AB_11154052
Human CD127 PE-Cy7 (clone eBioRDR5)	Thermo Fisher Scientific	Cat# 25-1278-42, RRID:AB_1659672
Human CD294 AF647 (clone BM16)	BD Biosciences	Cat# 558042, RRID:AB_2112699
Human CD161 AF700 (clone HP-3G10)	BioLegend	Cat# 339942, RRID:AB_2565870
Human CD94 APC-Fire750 (clone DX22)	BioLegend	Cat# A305-518A, RRID:AB_2773751
Human CD335 BV421 (clone 9E2/NKp46)	BD Biosciences	Cat# 564065, RRID:AB_2738572
Human CD45RA BV570 (clone HI100)	BioLegend	Cat# 304132, RRID:AB_2563813
Human CD117 BV605 (clone 104D2)	BioLegend	Cat# 313218, RRID:AB_2562025
Human CD3 BV650 (clone UCHT1)	BD Biosciences	Cat# 563852
Human CD7 BV711 (clone M-T701)	BD Biosciences	Cat# 564018, RRID:AB_2738544
Human T-bet BV786 (clone O4-46)	BD Biosciences	Cat# 564141, RRID:AB_2738615
Human CD19 BUV395 (clone SJ25C1)	BD Biosciences	Cat# 563549, RRID:AB_2738272
Human CD16 BUV496 (clone 3G8)	BD Biosciences	Cat# 564653, RRID:AB_2744294
Human CD25 BUV563 (clone 2A3)	BD Biosciences	Cat# 565699, RRID:AB_2744341
Human CD56 BUV737 (clone NCAM16.2)	BD Biosciences	Cat# 564447, RRID:AB_2744432
Human CD45 BUV805 (clone HI30)	BD Biosciences	Cat# 612891, RRID:AB_2870179
Human CD11c (S-HCL-3)	BioLegend	Cat# 371523, RRID:AB_2814332
Human CD141 (M80)	BioLegend	Cat# 344127, RRID:AB_2832671
Human CD161 (HP-3G10)	BioLegend	Cat# 339949, RRID:AB_2832665
Human CD14 (Me5E2)	BioLegend	Cat# 301857, RRID:AB_2800735
Human CD16 (3G8)	BioLegend	Cat# 302063, RRID:AB_2800737
Human CD19 (HIB19)	BioLegend	Cat# 302263, RRID:AB_2800740
Human CD1c (L161)	BioLegend	Cat# 331549, RRID:AB_2832653
Human CD28 (CD28.2)	BioLegend	Cat# 302961, RRID:AB_2800750
Human CD370 (CLEC9A) (8F9)	BioLegend	Cat# 353811, RRID:AB_2876671
Human CD38 (HB-7)	BioLegend	Cat# 356639, RRID:AB_2814303
Human CD3e (UCHT1)	BioLegend	Cat# 300477, RRID:AB_2800722
Human CD4 (RPA-T4)	BioLegend	Cat# 300565, RRID:AB_2800724
Human CD45RA (HI100)	BioLegend	Cat# 304161, RRID:AB_2800763
Human CD45RO (UCHL1)	BioLegend	Cat# 304259, RRID:AB_2800766
Human CD56 (NCAM) (5.1H11)	BioLegend	Cat# 362561, RRID:AB_2814309
Human CD66b (6/40c)	BioLegend	Cat# 392913, RRID:AB_2832738
Human CD69 (FN50)	BioLegend	Cat# 310949, RRID:AB_2800809
Human CD8a (RPA-T8)	BioLegend	Cat# 301069, RRID:AB_2800729
Human TCR gd (B1)	BioLegend	Cat# 331233, RRID:AB_2814200
Human TCR Va7.2 (3C10)	BioLegend	Cat# 351737, RRID:AB_2819993
IgG1 k Isotype Ctl (MOPC-21).	BioLegend	Cat# 400185
89 Y Human CD45 (clone HI30) PBMC panel 1	Fluidigm	Cat# 3089003B, RRID:AB_2661851
113 In Human CD57 (clone HCD57) PBMC panel 1	BioLegend	Cat# 322302, RRID:AB_535988
115 In Human CD11c (clone Bu15) PBMC panel 1	BioLegend	Cat# 337202, RRID:AB_1236381
141 Pr Human CD33 (clone WM53) PBMC panel 1	BioLegend	Cat# 303410, RRID:AB_2074243
142 Nd Human CD19 (clone HIB19) PBMC panel 1	BioLegend	Cat# 302202, RRID:AB_314232
143 Nd Human CD45RA (clone HI100) PBMC panel 1	BioLegend	Cat# 304102, RRID:AB_314406
144 Nd Human CD141 (clone M80) PBMC panel 1	BioLegend	Cat# 344102, RRID:AB_2201808
145 Nd Human CD4 (clone RPA-T4) PBMC panel 1	BioLegend	Cat# 300502, RRID:AB_314070
146 Nd Human CD8 (clone RPA-T8) PBMC panel 1	BioLegend	Cat# 301002, RRID:AB_314120
147 Sm Human CD20 (clone 2H7) PBMC panel 1	BioLegend	Cat# 302302, RRID:AB_314250
148 Nd Human CD16 (clone 3G8) PBMC panel 1	BioLegend	Cat# 302014, RRID:AB_314214
149 Sm Human CD127 (clone A019D5) PBMC panel 1	Fluidigm	Cat# 3149011B, RRID:AB_2661792
150 Nd Human CD1c (clone L161) PBMC panel 1	BioLegend	Cat# 331502, RRID:AB_1088995
151 Eu Human CD123 (clone 6H6) PBMC panel 1	BioLegend	Cat# 306002, RRID:AB_314576
152 Sm Human CD66b (clone G10F5) PBMC panel 1	BioLegend	Cat# 305102, RRID:AB_314494
153 Eu Human PD-1 (clone EH12.2H7) PBMC panel 1	BioLegend	Cat# 329926, RRID:AB_11147365
154 Sm Human CD86 (clone IT2.2) PBMC panel 1	BioLegend	Cat# 305410, RRID:AB_314530
155 Gd Human CD27 (clone O323) PBMC panel 1	BioLegend	Cat# 302802, RRID:AB_314294
156 Gd Human CCR5 (clone J418F1) PBMC panel 1	BioLegend	Cat# 359102, RRID:AB_2562457
158 Gd Human CD117 (clone 104D2) PBMC panel 1	BioLegend	Cat# 313202, RRID:AB_314981
159 Tb Human CD24 (clone ML5) PBMC panel 1	BioLegend	Cat# 311102, RRID:AB_314851
160 Gd Human CD14 (clone M5E2) PBMC panel 1	BioLegend	Cat# 301810, RRID:AB_314192
161 Dy Human CD56 (clone B159) PBMC panel 1	BD Biosciences	Cat# 555513, RRID:AB_395903
162 Dy Human gdTCR (clone REA591) PBMC panel 1	Miltenyi	Cat# 130-122-291; RRID: AB_2801872
163 Dy Human CRTh2 (clone REA598) PBMC panel 1	Milentyi	Cat# 130-122-305, RRID:AB_2801886
164 Dy Human CLEC12A (clone 50C1) PBMC panel 1	BioLegend	Cat# 353602, RRID:AB_10962440
165 Ho Human CCR6 (clone G034E3) PBMC panel 1	BioLegend	Cat# 353402, RRID:AB_10918625
166 Er Human CD25 (clone M-A251) PBMC panel 1	BioLegend	Cat# 356102, RRID:AB_2561752
167 Er Human CCR7 (clone G043H7) PBMC panel 1	BioLegend	Cat# 353256, RRID:AB_2814291
168 Er Human CD3 (clone UCHT1) PBMC panel 1	BioLegend	Cat# 300402, RRID:AB_314056
169 Tm Human CX3CR1 (clone 2A9-1) PBMC panel 1	BioLegend	Cat# 341602, RRID:AB_1595422
170 Er Human CD38 (clone HB-7) PBMC panel 1	BioLegend	Cat# 356602, RRID:AB_2561794
171 Yb Human CD161 (clone HP-3G10) PBMC panel 1	BioLegend	Cat# 339902, RRID:AB_1501090
172 Yb Human CD209 (clone 9E9A8) PBMC panel 1	BioLegend	Cat# 330102, RRID:AB_1134253
173 Yb Human CXCR3 (clone REA232) PBMC panel 1	Miltenyi	Cat# 130-108-022, RRID:AB_2655743
174 Yb Human HLADR (clone L243) PBMC panel 1	BioLegend	Cat# 307602, RRID:AB_314680
176 Yb Human CCR4 (clone 205410) PBMC panel 1	R&DSystems	Cat# MAB1567, RRID:AB_2074395
209 Bi Human CD11b (clone ICRF44) PBMC panel 1	Fluidigm	Cat# 3209003B, RRID:AB_2687654
89 Y Human CD45 (clone HI30) PBMC panel 2	Fluidigm	Cat# 3089003B, RRID:AB_2661851
113 In Human HLA-ABC (clone W6/32) PBMC panel 2	BioLegend	Cat# 311402, RRID:AB_314871
115 In Human CD11c (clone Bu15) PBMC panel 2	BioLegend	Cat# 337202, RRID:AB_1236381
141 Pr Human CD33 (clone WM53) PBMC panel 2	BioLegend	Cat# 303410, RRID:AB_2074243
142 Nd Human CD19 (clone HIB19) PBMC panel 2	BioLegend	Cat# 302202, RRID:AB_314232
143 Nd Human CD45RA (clone HI100) PBMC panel 2	BioLegend	Cat# 304102, RRID:AB_314406
144 Nd Human CD141 (clone M80) PBMC panel 2	BioLegend	Cat# 344102, RRID:AB_2201808
145 Nd Human CD4 (clone RPA-T4) PBMC panel 2	BioLegend	Cat# 300502, RRID:AB_314070
146 Nd Human CD8 (clone RPA-T8) PBMC panel 2	BioLegend	Cat# 301002, RRID:AB_314120
147 Sm Human CLEC9A (clone 8F9) PBMC panel 2	BioLegend	Cat# 353802, RRID:AB_10983070
148 Nd Human CD16 (clone 3G8) PBMC panel 2	BioLegend	Cat# 302014, RRID:AB_314214
149 Sm Human FceRIa (clone AER-37) PBMC panel 2	BioLegend	Cat# 334602, RRID:AB_1227649
150 Nd Human CD1c (clone L161) PBMC panel 2	BioLegend	Cat# 331502, RRID:AB_1088995
151 Eu Human CD123 (clone 6H6) PBMC panel 2	BioLegend	Cat# 306002, RRID:AB_314576
152 Sm Human CD66b (clone G10F5) PBMC panel 2	BioLegend	Cat# 305102, RRID:AB_314494
153 Eu Human CD83 (clone HB15e) PBMC panel 2	BioLegend	Cat# 305302, RRID:AB_314510
154 Sm Human CD86 (clone IT2.2) PBMC panel 2	BioLegend	Cat# 305410, RRID:AB_314530
155 Gd Human CD27 (clone O323) PBMC panel 2	BioLegend	Cat# 302802, RRID:AB_314294
156 Gd Human PD-L1 (clone 29E.2A3) PBMC panel 2	BioLegend	Cat# 329711, RRID:AB_2228868
158 Gd Human CD163 (clone REA812) PBMC panel 2	Miltenyi	Cat# 130-122-293, RRID:AB_2801874
159 Tb Human CD103 (clone Ber-Act8) PBMC panel 2	BioLegend	Cat# 350202, RRID:AB_10639864
160 Gd Human CD14 (clone M5E2) PBMC panel 2	BioLegend	Cat# 301810, RRID:AB_314192
161 Dy Human CD56 (clone B159) PBMC panel 2	BD Biosciences	Cat# 555513, RRID:AB_395903
162 Dy Human CD64 (clone 10,1) PBMC panel 2	BioLegend	Cat# 305016, RRID:AB_2103461
163 Dy Human CD172a/b (clone SE5A5) PBMC panel 2	Fluidigm	Cat# 3163017B, RRID:AB_2864730
164 Dy Human CD40 (clone HB14) PBMC panel 2	BioLegend	Cat# 334302, RRID:AB_1236384
166 Er Human CD169 (clone 7-239) PBMC panel 2	BioLegend	Cat# 346002, RRID:AB_2189031
167 Er Human CD117 (clone 104D2) PBMC panel 2	BioLegend	Cat# 313202, RRID:AB_314981
168 Er Human CD3 (clone UCHT1) PBMC panel 2	BioLegend	Cat# 300402, RRID:AB_314056
169 Tm Human CX3CR1 (clone 2A9-1) PBMC panel 2	BioLegend	Cat# 341602, RRID:AB_1595422
170 Er Human CD38 (clone HB-7) PBMC panel 2	BioLegend	Cat# 356602, RRID:AB_2561794
171 Yb Human CD207 (clone 1000) PBMC panel 2	BioLegend	Cat# 352202, RRID:AB_10898115
172 Yb Human CD206 (clone 44607) PBMC panel 2	BioLegend	Cat# 321112, RRID:AB_571921
174 Yb Human HLADR (clone L243) PBMC panel 2	BioLegend	Cat# 307602, RRID:AB_314680
175 Lu Human Axl (clone 108724) PBMC panel 2	R&DSystems	Cat# MAB154, RRID:AB_2062558
176 Yb Human CD209 (clone 9E9A8) PBMC panel 2	BioLegend	Cat# 330102, RRID:AB_1134253
209 Bi Human CD11b (clone ICRF44) PBMC panel 2	Fluidigm	Cat# 3209003B, RRID:AB_2687654
163Dy Human CXCR3 (clone G025H7) Whole blood custom panel	Fluidigm	Cat# 3163004B, RRID:AB_2810969
152Sm Human TCRgd (clone 11F2) Whole blood custom panel	Fluidigm	Cat# 3152008B, RRID:AB_2687643
142Nd Human CD19 (clone HIB19) Whole blood custom panel	Fluidigm	Cat# 3142001B, RRID:AB_2651155
144Nd Human CD38 (clone HIT2) Whole blood custom panel	Fluidigm	Cat# 3144014B, RRID:AB_2687640
151Eu Human CD123 (clone 6H6) Whole blood custom panel	Fluidigm	Cat# 3151001B, RRID:AB_2661794
153Eu Human Va7.2 (clone 3C10) Whole blood custom panel	Fluidigm	Cat# 3153024B, RRID:AB_2891190
154Sm Human CD3 (clone UCHT1) Whole blood custom panel	Fluidigm	Cat# 3154003B, RRID:AB_2811086
155Gd Human CD45RA (clone HI100) Whole blood custom panel	Fluidigm	Cat# 3155011B, RRID:AB_2810246
158Gd Human CD27 (clone L128) Whole blood custom panel	Fluidigm	Cat# 3158010B, RRID:AB_2858231
159Tb Human CD1c (clone L161) Whole blood custom panel	Biolegend	Cat# 331502, RRID:AB_1088995
161Dy Human CLEC9A (clone 8F9) Whole blood custom panel	Fluidigm	Cat# 3161018B, RRID:AB_2810252
164Dy Human CD161 (clone HP-3G10) Whole blood custom panel	Fluidigm	Cat# 3164009B, RRID:AB_2687651
168Er Human CD8 (clone SK1) Whole blood custom panel	Fluidigm	Cat# 3168002B, RRID:AB_2892771
170Er Human iNKT (clone 6B11) Whole blood custom panel	Fluidigm	Cat# 3170015B
175Lu Human CCR4 (clone L291H4) Whole blood custom panel	Fluidigm	Cat# 3175035A, RRID:AB_2921320
174Yb Human CD4 (clone RPA-T4) Whole blood custom panel	Biolegend	Cat# 300502, RRID:AB_314070
162Dy Human CD21 (clone REA940) Whole blood custom panel	Miltenyi Biotec Inc.	Cat# 130-124-315, RRID:AB_2811646
165Ho Human NKG2C (clone REA205) Whole blood custom panel	Miltenyi Biotec Inc.	Cat# 130-122-278, RRID:AB_2801859
148Nd Human CD20 (clone 2H7) Whole blood custom panel	Biolegend	Cat# 302302, RRID:AB_314250
173Yb Human HLA-DR (clone L243) Whole blood custom panel	Fluidigm	Cat# 3173005B, RRID:AB_2810248
156Gd Human CCR10 (clone REA326) Whole blood custom panel	Miltenyi Biotec Inc.	Cat# 130-122-317, RRID:AB_2801898
089Y Human CD45 (clone HI30) Whole blood custom panel	Fluidigm	Cat# 3089003B, RRID:AB_2661851
116Cd Human CD66b (clone QA17A51) Whole blood custom panel	Biolegend	Cat# 396902, RRID:AB_2814367
141Pr Human CCR6 (clone G034E3) Whole blood custom panel	Fluidigm	Cat# 3141003A, RRID:AB_2687639
143Nd Human CD127 (clone A019D5) Whole blood custom panel	Fluidigm	Cat# 3143012B, RRID:AB_2810240
147Sm Human CD11c (clone Bu15) Whole blood custom panel	Fluidigm	Cat# 3147008B, RRID:AB_2687850
149Sm Human CD25 (clone 2A3) Whole blood custom panel	Fluidigm	Cat# 3149010B, RRID:AB_2756416
150Nd Human NKVFS1 (clone NKVFS1) Whole blood custom panel	Bio-Rad	Cat# MCA2243GA, RRID:AB_323743
167Er Human CCR7 (clone G043H7) Whole blood custom panel	Fluidigm	Cat# 3167009A, RRID:AB_2858236
169Tm Human NKG2A (clone Z199) Whole blood custom panel	Fluidigm	Cat# 3169013B, RRID:AB_2756426
171Yb Human CXCR5 (clone RF8B2) Whole blood custom panel	Fluidigm	Cat# 3171014B, RRID:AB_2858239
166Er Human CD24 (clone ML5) Whole blood custom panel	Fluidigm	Cat# 3166007B, RRID:AB_2661803
145Nd Human CD31 (clone WM59) Whole blood custom panel	Fluidigm	Cat# 3145004B, RRID:AB_2737262
160Gd Human CD14 (clone M5E2) Whole blood custom panel	Fluidigm	Cat# 3160001B, RRID:AB_2687634
176Yb Human CD56 (clone NCAM16.2) Whole blood custom panel	Fluidigm	Cat# 3176008B, RRID:AB_2661813
172Yb Human CD57 (clone HNK-1) Whole blood custom panel	Biolegend	Cat# 359602, RRID:AB_2562403
150Nd Human KIR3DL1L2 (clone REA970) Whole blood custom panel	Miltenyi Biotec Inc.	Cat# 130-126-489, RRID:AB_2889458
146Nd Human IgD (clone IA6-2) Whole blood custom panel	Fluidigm	Cat# 3146005B, RRID:AB_2811082
209Bi Human CD16 (clone 3G8) Whole blood custom panel	Fluidigm	Cat# 3209002B, RRID:AB_2756431
eFluor 450 Murine CD3ε (clone 1452C11)	Thermo Fisher Scientific	Cat# 48-0031-82, RRID:A14714
BUV395 Murine CD4 (clone GK1.5)	BD Bioscience	Cat# 563790, RRID:AB_2738426
PE Murine CD5 (clone 53-7.3)	Thermo Fisher Scientific	Cat# 12-0051-82, RRID:AB_465523
eFluor 615 Murine CD8α (clone 53-6.7)	Thermo Fisher Scientific	Cat# 47-0081-82, RRID:AB_1272185
Brilliant Violet 650 Murine CD11b (clone M1/70)	Thermo Fisher Scientific	Cat# 416-0112-82
PE Murine CD11b (clone M1/70)	Thermo Fisher Scientific	Cat# 12-0112-82, RRID:AB_2734869
eFluor 450 Murine CD11b (clone M1/70)	Thermo Fisher Scientific	Cat# 48-0112-82, RRID:AB_1582236
FITC Murine CD11c (clone N418)	Thermo Fisher Scientific	Cat# 11-0114-82, RRID:AB_464940
PE Murine CD11c (clone N418)	Thermo Fisher Scientific	Cat# 12-0114-82, RRID:AB_465552
eFluor 450 Murine CD11c (clone N418)	Thermo Fisher Scientific	Cat# 48-0114-82, RRID:AB_1548654
PE Murine CD19 (clone eBio1D3)	Thermo Fisher Scientific	Cat# 12-0193-82, RRID:AB_657659
Alexa Fluor 660 Murine CD19 (clone eBio1D3)	Thermo Fisher Scientific	Cat# 606-0193-82, RRID:AB_2896251
eFluor 450 Murine CD19 (clone eBio1D3)	Thermo Fisher Scientific	Cat# 48-0193-82, RRID:AB_2734905
Alexa Fluor 488 Murine CD25 (clone PC61.5)	Thermo Fisher Scientific	Cat# 53-0251-82, RRID:AB_763472
Brilliant Violet 711 Murine CD25 (clone PC61.5)	Thermo Fisher Scientific	Cat# 407-0251-82
PerCP-Cyanine5.5 Murine CD26 (clone H194-112)	Thermo Fisher Scientific	Cat# 45-0261-82, RRID:AB_1548738
Brilliant Violet 785 Murine CD44 (clone IM7)	BioLegend	Cat# 103059, RRID:AB_2571953
Brilliant Violet 785 Murine CD45 (clone 30-F11)	BioLegend	Cat# 103149, RRID:AB_2564590
PE/Cyanine7 Murine CD45 (clone 30-F11)	BioLegend	Cat# 103114, RRID:AB_312979
Brilliant Violet 711 Murine CD45 (clone 30-F11)	BioLegend	Cat# 103147, RRID:AB_2564383
Brilliant Violet 650 Murine CD45 (clone 30-F11)	BioLegend	Cat# 103151, RRID:AB_2565884
PE Murine CD45R (B220) (clone RA3-6B2)	Thermo Fisher Scientific	Cat# 12-0452-82, RRID:AB_465671
Brilliant Ultra Violet 395 Murine CD45R (B220) (clone RA3-6B2)	Thermo Fisher Scientific	Cat# 363-0452-82
eFluor 450 Murine CD45R (B220) (clone RA3-6B2)	Thermo Fisher Scientific	Cat# 48-0452-82, RRID:AB_1548761
eFluor 450 Murine CD49b (clone DX5)	Thermo Fisher Scientific	Cat# 48-5971-82, RRID:AB_10671541
PE/Dazzle 594 Murine CD62L (clone MEL-14)	BioLegend	Cat# 104448, RRID:AB_2566163
BV786 Murine CD64 (clone X54-5/7.1)	BD Biosciences	Cat# 741024; RRID:AB_2740644
APC Murine CD86 (clone GL-1)	BioLegend	Cat# 105012, RRID:AB_493342
Brilliant Violet 510 Murine CD90.2 (Thy-1.2) (clone 53-2.1)	BioLegend	Cat# 140319, RRID:AB_2561395
APC Murine CD117 (c-Kit) (clone 2B8)	Thermo Fisher Scientific	Cat# 17-1171-82, RRID:AB_469430
Brilliant Violet 711 Murine CD127 (clone A7R34)	BioLegend	Cat# 135035, RRID:AB_2564577
Brilliant Violet 650 Murine CD127 (clone A7R34)	BioLegend	Cat# 135043, RRID:AB_2629681
PE Murine CD135 (Flt3) (clone A2F10)	BioLegend	Cat# 135306, RRID:AB_1877217
APC Murine CD172a (SIRPa) (clone P84)	Thermo Fisher Scientific	Cat# 17-1721-82, RRID:AB_10733158
PE/Cyanine7 Murine CD317 (BST2, PDCA-1) (clone 927)	BioLegend	Cat# 348416, RRID:AB_2716221
PE/Dazzle 594 Murine F4/80 (clone BM8)	BioLegend	Cat# 123146, RRID:AB_2564133
PE Murine FcεR1α (clone 36951)	Thermo Fisher Scientific	Cat# 12-5898-82, RRID:AB_466028
eFluor 450 Murine FcεR1α (clone 36951)	Thermo Fisher Scientific	Cat# 48-5898-82, RRID:AB_2574086
FITC Murine FOXP3 (clone FJK-16s)	Thermo Fisher Scientific	Cat# 11-5773-82, RRID:AB_465243
eFluor 660 Murine GATA3 (clone TWAJ)	Thermo Fisher Scientific	Cat# 50-9966-42, RRID:AB_10596663
PE-Cyanine7 Murine KLRG1 (clone 2F1)	Thermo Fisher Scientific	Cat# 25-5893-82, RRID:AB_1518768
Biotin Murine Integrin α4β7 (clone DATK32)	BioLegend	Cat# 120612, RRID:AB_11203892
Alexa Fluor 488 Murine Ly-6A/E (Sca-1) (clone E13-161.7)	BioLegend	Cat# 122516, RRID:AB_756201
Murine Ly6C (clone AL-21)	BD Biosciences	Cat# 553104, RRID:AB_394628
PE Murine Ly6G/Ly6C (Gr-1) (clone RB6-8C5)	Thermo Fisher Scientific	Cat# 12-5931-82, RRID:AB_466045
eFluor 450 Murine Ly6G/Ly6C (Gr-1) (clone RB6-8C5)	Thermo Fisher Scientific	Cat# 48-5931-82, RRID:AB_1548788
PerCP-Cy5.5 Murine Ly6G (clone 1A8)	BD Biosciences	Cat# 560602; RRID:AB_1727563
BUV805 Murine MHC Class II (I-A/I-E) (clone M5/114)	BD Biosciences	Cat# 748844; RRID:AB_2873247
PE-Cyanine7 Murine NK1.1 (clone PK136)	Thermo Fisher Scientific	Cat# 25-5941-82, RRID:AB_469665
eFluor 450 Murine NK1.1 (clone PK136)	Thermo Fisher Scientific	Cat# 48-5941-82, RRID:AB_2043877
PerCP-Cyanine5.5 Murine NK1.1 (clone PK136)	Thermo Fisher Scientific	Cat# 45-5941-82, RRID:AB_914361
PE Murine Siglec-F (clone E50-2440)	BD Biosciences	Cat# 562068; RRID:AB_394341
PerCP-eFluor 710 Murine IL-33R (ST2) (clone RMST2-2)	Thermo Fisher Scientific	Cat# 46-9335-82, RRID:AB_2573883
eFluor 450 Murine TCRβ (clone H57-597)	Thermo Fisher Scientific	Cat# 48-5961-82, RRID:AB_11039532
PE Murine TCRβ (clone H57-597)	Thermo Fisher Scientific	Cat# 12-5961-82, RRID:AB_466066
eFluor 450 Murine TCR γ/δ (clone eBioGL3)	Thermo Fisher Scientific	Cat# 48-5711-82, RRID:AB_2574071
PE Murine TCR γ/δ (clone eBioGL3)	Thermo Fisher Scientific	Cat# 12-5711-82, RRID:AB_465934
eFluor 450 Murine TER-119 (clone TER-119)	Thermo Fisher Scientific	Cat# 48-5921-82, RRID:AB_1518808
PE-Cyanine7 Murine TER-119 (clone TER-119)	Thermo Fisher Scientific	Cat# 25-5921-82, RRID:AB_469661
Murine XCR-1 (clone ZET)	Biolegend	Cat# 148204, RRID:AB_2563843
**Bacterial and virus strains**		
*Salmonella enterica* subsp. *enterica* serovar Typhimurium GFP	ATCC	14028GFP
*Mycobacterium abscessus* sensu stricto, strain CIP104536T, smooth with pTEC27	Bernut *et al*., 2014^212^	N/A
expanded T7 Virscan phage library	S. Elledge (Brigham and Women’s Hospital and Harvard University Medical School, Boston, MA, USA)	VirScan Phage Library, version 3
Vesicular stomatitis virus Indiana (VSV)	Bastard *et al*., 2021^143^	N/A
Human immunodeficiency viruses-1 (HIV-1) reporter virus (NL4-3 ΔenvΔnef encoding GFP in nef)	Bhargava *et al*., 2021^171^	N/A
Human immunodeficiency viruses-2 (HIV2) reporter virus (ROD9 ΔenvΔnef encoding GFP in nef)	Manel *et al*., 2010^173^	N/A
Severe acute respiratory syndrome coronavirus 2 (SARS-CoV-2) NYC isolate	Zhang *et al*., 2020^138^	GenBank OM345241
Yellow-fever vaccine virus-venus (YF17D-venus) reporter	Yi *et al*., 2011^179^	N/A
Influenza A virus A/California/4/2009 (IAV)	Manicassamy *et al*., 2010^213^	N/A
Herpes simplex virus 1 (HSV-1)	ATCC	VR-1493
Hepatitis A virus (HAV) reporter virus (HM175/18f-NLuc)	Yamane *et al*., 2019^52^	N/A
Encephalomyocarditis virus (EMCV)	Gao *et al*., 2021^187^	N/A
**Biological samples**		
Peripheral blood mononuclear cells from indicated individuals	This manuscript	N/A
Plasma from indicated individuals	This manuscript	N/A
Biopsies from indicated individuals	This manuscript	N/A
**Chemicals, peptides, and recombinant proteins**		
Protamine sulfate	Merck	Cat# P3369-10G
Recombinant interferon gamma-1b (Imukin)	Clinigen Healthcare France	Cat# 3400955776789
Recombinant interferon alpha-2b (Introna)	MSD France	Cat# 3400934956287
Aldesleukin (Proleukin)	Novartis	Cat# 3400956215867
Recombinant Human IFN-beta 1a (Mammalian) Protein	Bio-Techne	Cat# 11410-2
Collagenase IV	Gibco	Cat# 17104-019
DpnI	New England Biolab	Cat# R0176L
M-CSF	Peprotech	Cat# 300-25
IL-3	Peprotech	Cat# 200-03
M-CSF	# 204-IL-010	Cat# 216-MC-010
IL-4	R&D Systems	Cat# 204-IL-010
ProLong Gold with DAPI	Thermo Fisher Scientific	Cat #P36931
Aqua Dead Cell Stain kit	Thermo Fisher Scientific	Cat# L34957
**Critical commercial assays**		
SureSelect Human All Exon V6	Agilent	Cat# 5190-8864
Human SNP Array 6.0	Agilent	Cat# 901153
GoTaq DNA Polymerase	Promega	Cat# M3005
RNeasy Plus Mini Kit	Qiagen	Cat# 74136
Quick-RNA MicroPrep Kit	Zymo	Cat# R1051
Universal PCR Master Mix (2X), no AmpErase UNG	Thermo Fisher Scientific	Cat# 4352042
RiboZero TruSeq Stranded Total RNA Library Prep Kit	Illumina	Cat# 20020596
ELISA IL-12p40	R&D Systems	Cat# DP400
ELISA IL-12p70 HS	R&D Systems	Cat# HS120
NE-PER nuclear and cytoplasmic extraction reagents	Thermo Fisher Scientific	Cat #78835
RNeasy Plus Mini Kit	Qiagen	Cat# 74136
Quick-RNA MicroPrep Kit	Zymo	Cat# R1051
SuperScript II Reverse Transcriptase	Thermo Fisher Scientific	Cat# 18064014
High-Capacity RNA-to-cDNA Kit	Applied Biosystems	Cat# 4387406
TaqMan Fast Universal PCR Master Mix (2X), no AmpErase UNG	Thermo Fisher Scientific	Cat# 4352042,
**Deposited data**		
RNA sequencing on primary fibroblasts	This manuscript	gene expression omnibus (GEO: GSE218033)
RNA sequencing on iPSC-derived macrophages	This manuscript	gene expression omnibus (GEO: GSE218033)
RNA sequencing on monocyte-derived macrophages	This manuscript	gene expression omnibus (GEO: GSE218033)
Mass-spectrometry on primary fibroblasts	This manuscript	ProteomeXchange (PXD037759)
scRNAseq on cryopreserved PBMCs	This manuscript	gene expression omnibus (GEO: GSE216489)
**Experimental models: Cell lines**		
HEK293T cells	ATCC	Cat# CRL-11268, RRID:CVCL_1926
THP1 WT	Song *et al*., 2021^78^	ATCC Cat# TIB-202, RRID:CVCL_0006
THP1 IRF1^KO^	Song *et al*., 2021^78^	N/A
THP1 IFN-γR1^KO^ clone 30	This manuscript	N/A
THP1 STAT1^KO^ clone 13	This manuscript	N/A
iPSC healthy control clone 11	Ackermann *et al*., 2014^191^	hCD34-iPSC11
iPSC healthy control clone 16	Lachmann *et al*., 2014^189^	hCD34-iPSC16
iPSC P1 clone 10	This manuscript	N/A
iPSC P2 clone 3	This manuscript	N/A
iPSC STAT1	Haake *et al*., 2020^50^	iSTAT1_compl
**Experimental models: Organisms/strains**		
C57BL/6 (B6) WT	This manuscript	In-house colony established from Jax # 000664
C57BL/6 (B6) IRF1−/−	This manuscript	Jax # 002762 backcrossed to a B6 background
**Oligonucleotides**		
*IRF1* genomic exon 3F	ThermoFischer Scientific	TGGTCTGTTTAAGCCAGCCTC
*IRF1* genomic exon 3R	ThermoFischer Scientific	CAGAAACACAAGTCTGCCACC
*IRF1* genomic exon 5F	ThermoFischer Scientific	TTCCACCTCTCACCAAGAACC
*IRF1* genomic exon 5R	ThermoFischer Scientific	CAGAGAAGGTATCAGGGCTGG
IRD700-conjugated ISRE probe-F	Metabion	GATCGGGAAAGGGAAACCGAAACTGAA
IRD700-conjugated ISRE probe-R	Metabion	TCAGTTTCGGTTTCCCTTTCCCGATC
oligo(dT)_12–18_	Thermo Fisher Scientific	Cat# 18418012
*IRF1* exons 3–4 qPCR probe	ThermoFischer Scientific	Cat# Hs00971960_m1
*IRF1* exons 8–9	ThermoFischer Scientific	Cat# Hs00971965_m1
*GBP4*	ThermoFischer Scientific	Cat# Hs00364728_m1
*APOL3*	ThermoFischer Scientific	Cat# Hs00758274_m1
*GUSB*	ThermoFischer Scientific	Cat# 1702016
IRF1-p.M85A-DDK-F	Eurofins	CAACTTTCGCTGTGCCGCGAACTCCCT GCCAGAT
IRF1-p.M85A-DDK-R	Eurofins	ATCTGGCAGGGAGTTCGCGGCACAGC GAAAGTTG
IRF1-p.M111A-DDK-F	Eurofins	GCGAGTGTACCGGGCGCTTCCACCTCTC
IRF1-p.M111A-DDK-R	Eurofins	GAGAGGTGGAAGCGCCCGGTACACTCGC
IRF1-p.Q35*-F	Eurofins	GAGGAGATGATCTTCTAGATCCCATGG AAGC
IRF1-p.Q35*-R	Eurofins	GCTTCCATGGGATCTAGAAGATCATCT CCTC
IRF1-p.R129*-F	ThermoFischer Scientific	GTCGAAGTCCAGCTGAGATGCTAAG
IRF1-p.R129*-R	ThermoFischer Scientific	CTTAGCATCTCAGCTGGACTTCGAC
IRF1-p.R129-DDK-F	ThermoFischer Scientific	GCTGGACTTCGACTTTCTTTCTTTTCTCTG
IRF1-p.R129-DDK-R	ThermoFischer Scientific	ACGCGTACGCGGCCGCTCGA
IRF1-p.W11R-DDK-F	ThermoFischer Scientific	GCATGAGACCCCGGCTAGAGATG
IRF1-p.W11R-DDK-R	ThermoFischer Scientific	CATCTCTAGCCGGGGTCTCATGC
IRF1-Δ7-8-F	ThermoFischer Scientific	CTGGAGTCAGGGCCTGCTCC
IRF1-Δ7-8-R	ThermoFischer Scientific	CTCTTGGAGCAGTCGGAGTGGC
IRF1-Δ7-8-DDK-F	ThermoFischer Scientific	TTGAGTAGGTACCCCTTCCCATCCACGTTTG
IRF1-Δ7-8-DDK-R	ThermoFischer Scientific	ACGCGTACGCGGCCGCTCGA
IRF1-p.A67P-DDK-F	ThermoFischer Scientific	GCCGATACAAACCAGGGGAAAAG
IRF1-p.A67P-DDK-R	ThermoFischer Scientific	CTTTTCCCCTGGTTTGTATCGGC
IRF1-p.M1_A84del-F	Eurofins	ATGAACTCCCTGCCAGATAT
IRF1-p.M1_A84del-R	Eurofins	GGCGATCGCGGCGGCAGATC
IRF1 retrovirus F	ThermoFischer Scientific	GATCCATTTAAATTCGAATTCATGCCCA TCACTCGGATGCGC
IRF1 retrovirus R	ThermoFischer Scientific	ATCGATACCGTCGACCTCGAGTTAAAC CTTATCGTCGTCATC
sgRNA IFNGR1 exon 3F	Eurofins	CACCGACACATTCTACTCACCATCT
sgRNA IFNGR1 exon 3R	Eurofins	AAACAGATGGTGAGTAGAATGTGTA
sgRNA STAT1 exon 3F	Eurofins	CACCGTATTTGCAGCTCGTTTGTGG
sgRNA STAT1 exon 3R	Eurofins	AAACCCACAAACGAGCTGCAAATAA
Recombinant DNA		
pCMV6-EV	Origene	Cat# RCPS100001
pCMV6-IRF1-WT-DDK	Origene	Cat# RC203500
pCMV6-IRF1-p.M85A-DDK	This manuscript	N/A
pCMV6-IRF1-p.M111A-DDK	This manuscript	N/A
pCMV6-IRF1-M85A/M111A-DDK	This manuscript	N/A
pCMV6-IRF1-p.Q35*	This manuscript	N/A
pCMV6-IRF1-p.Q35*/M85A-DDK	This manuscript	N/A
pCMV6-IRF1-p.Q35*/M111A-DDK	This manuscript	N/A
pCMV6-IRF1-p.Q35*/M85A/M111A-DDK	This manuscript	N/A
pCMV6-IRF1-p.R129*	This manuscript	N/A
pCMV6-IRF1-p.R129-DDK	This manuscript	N/A
pCMV6-IRF1-p.W11R-DDK	This manuscript	N/A
pCMV6-IRF1-Δ7-8	This manuscript	N/A
pCMV6-IRF1-Δ7-8-DDK	This manuscript	N/A
pCMV6-IRF1-p.A67P-DDK	This manuscript	N/A
pCMV6-IRF1-p.M1_A84del	This manuscript	N/A
pGL4.10[luc2] backbone with three GGAAAGGGAAACCGAAACTGAA repeats	Guerin *et al*., 2018^158^	Cat# E6651
pGL4.10[luc2] backbon with five GGGAAAGTGAAACTA repeats	Hernandez *et al*., 2018^183^	N/A
pRL-SV40	Promega	Cat# E2231
lentiCRISPR v2	Addgene	Cat# 52961
psPAX2	Addgene	Cat# 12260
pCMV-VSV-G	Addgene	Cat# 8454
pHXB2-Env	NIH-AIDS Reagent Program	Cat# 1069
**Software and algorithms**		
phip-stat	Larman *et al*., 2011^214^	https://github.com/lasersonlab/phip-stat
Bowtie for alignment of PhIP-Seq raw reads	Larman *et al*., 2011^214^	http://bowtie-bio.sourceforge.net/index.shtml
R	The R Project for Statistical Computing	https://www.r-project.org
DESeq2	Love *et al*., 2014^201^	https://bioconductor.org/packages/release/bioc/html/DESeq2.html
Uniform Manifold Approximation and Projection (UMAP)	Becht *et al*., 2018^208^	v.0.3.5
BVAtools	https://bitbucket.org/mugqic/bvatools	https://bitbucket.org/mugqic/bvatools
Cell Ranger	10X Genomics	v3.0.1 for scRNA and v6.0.1 for CITE-seq
DoubletFinder package	McGinnis *et al*., 2019^206^	v2.0.3
Seurat R package	Stuart *et al*., 2019^207^	v4.0.2
MAST	Finak *et al*., 2015^209^	https://github.com/RGLab/MAST
gProfiler R package	Raudvere *et al*., 2019^210^	https://biit.cs.ut.ee/gprofiler/gost
FastQC	https://www.bioinformatics.babraham.ac.uk/projects/fastqc/	https://www.bioinformatics.babraham.ac.uk/projects/fastqc/
STAR	Dobin *et al*., 2013^197^	v2.7.3a
RSeQC	Wang *et al*., 2012^198^	v0.11.2
HTSeq-count	Anders *et al*., 2015^199^	v3.26.8
edgeR package	McCarthy *et al*., 2012^200^	N/A
HOMER	Heinz *et al*., 2010^202^	v4.11

## Highlights

- Inherited complete human IRF1 deficiency underlies severe mycobacterial disease.
- Human IRF1 is essential for IFN-γ-dependent macrophagic immunity to mycobacteria.
- Human IRF1 is essential for IFN-γ- and STAT1-dependent immunity to mycobacteria.
- Human IRF1 is largely redundant for IFN-α/β-dependent antiviral intrinsic immunity.
